# Dissipation in the broadband and ultrastrong coupling regimes of cavity quantum electrodynamics: an ab initio quantized quasinormal mode approach

**DOI:** 10.1038/s41377-026-02406-2

**Published:** 2026-08-26

**Authors:** Chris Gustin, Juanjuan Ren, Sebastian Franke, Stephen Hughes

**Affiliations:** 1 E. L. Ginzton Laboratory, Stanford University, Stanford, CA USA; 2 Department of Physics, Engineering Physics, and Astronomy, Queen’s University, Kingston, ON Canada; 3 Technische Universität Berlin, Institut für Theoretische Physik, Nichtlineare Optik und Quantenelektronik, Berlin, Germany; 4 Present Address: Center for Hybrid Quantum Networks (Hy-Q), Niels Bohr Institute, University of Copenhagen, Copenhagen, Denmark

**Keywords:** Quantum optics, Quantum optics

## Abstract

Phenomenological approaches to photon loss have long been the workhorse of cavity-QED, but prove inadequate in the presence of sufficiently broadband light-matter interactions. We present a rigorous and ab initio derivation of a quantum master equation for a quantized optical cavity mode coupled to a dipole, using a quasinormal mode (QNM) quantization procedure for plasmonic and dielectric open-system cavity-QED, which is valid in broadband light-matter interaction regimes, including ultrastrong coupling (USC). The theory supports general three-dimensional resonators with arbitrary dispersion and loss, and thus can be applied to a wide range of realistic open cavities. Our ab initio and gauge-invariant approach fully recovers a recent result for the spectral density of a quantized cavity with a single dipole and reveals important departures from previous heuristic assumptions about system-reservoir coupling. We identify a new criterion for what we term the “broadband dissipative” regime of cavity-QED, where phenomenological models require corrections in accordance with the intrinsic and spatially-dependent complex phase of the QNM, and also shed light on *fundamental limits* to single-mode models in extreme coupling regimes. Using experimentally motivated plasmonic and dielectric cavity examples, we show validity ranges of our QNM master equation and spectral USC calculations, and discuss prospects for near-term experimental observation of the broadband dissipative effects.

## Abstract (web summary)

By advancing the theory of dissipation from quantized open cavities, both ultrastrong coupling and an emerging "broadband dissipative" regime of cavity quantum electrodynamics can be efficiently characterized and modeled.

## Graphical abstract

**Figure:**
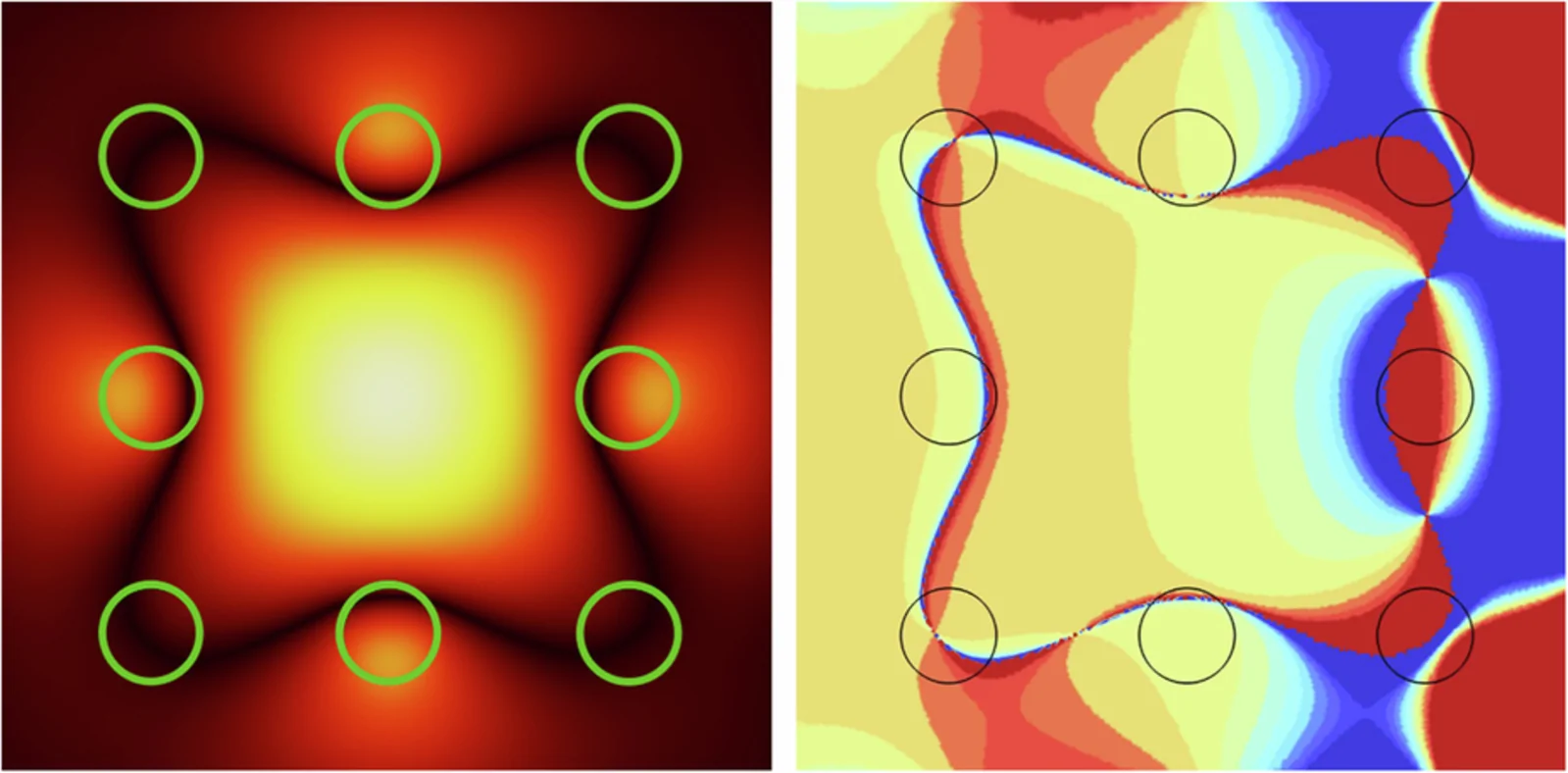

## Introduction

Cavity quantum electrodynamics (cavity-QED) is a ubiquitous platform both for fundamental research and quantum information processing technologies^1–4^. Photons are ideal carriers of quantum information, which travel rapidly with minimal decoherence at room temperature, and are naturally transmittable along classical channels such as waveguides and fibers. Interfacing with atoms—artificial or real—allows for the implementation of nonlinear and often deterministic quantum operations, while engineered photonic structures can mold the flow of light and resonances to enable both a greater degree of unitary control over the system, as well as system-reservoir engineering^5–7^.

Over the decades, canonical theoretical tools have been built up alongside experimental and technological progress in cavity-QED, which enable intuitive, computationally inexpensive, and precise modeling of the quantum dynamics^8–11^. Such theoretical progress has undoubtedly contributed to the growth of the field, providing researchers with effective tools with which to predict new physical effects, develop new quantum protocols, and understand experimental results. Milestones along this path range from the Jaynes-Cummings^12^ and Dicke^13,14^ models (unitary) to system-reservoir and input-output theory^8,15,16^ (dissipative).

In particular, open quantum system theory has allowed for realistic and concise treatment of photon loss, intrinsic in any physical cavity-QED system. A particular powerful approach has been the Lindblad master equation (ME) (and related techniques—e.g., the stochastic Schrödinger equation), which, for example, can describe Markovian photon loss from quantized cavity modes into a photon reservoir without the need to include a continuum Hilbert space in final calculations, and while retaining the important physical information associated with the present discrete electromagnetic (EM) resonances^11^.

Such *system-reservoir* theories have proven extremely effective in connecting unitary quantum dynamics to the dissipative behavior seen in reality. However, while the theory of open quantum systems can generically treat couplings between a system and its environment by means of a *spectral density* function Λ^2^(*ω*) ^11^, when it comes to photon loss, such system-reservoir theories have almost invariably to date relied on *phenomenological* assumptions on the form of the interaction Hamiltonian which couples an EM system (e.g., modes of a quantized cavity or waveguide) to its reservoir. For a long time, such an approach proved sufficient, as the highly Markovian nature of photon decay (occurring over timescales many orders of magnitude larger than the photon period) meant that the only relevant parameter to the process (aside from photonic Lamb shifts which can be absorbed into the observed system frequencies) was a single value Λ^2^(*ω*_*c*_), where *ω*_*c*_ is the frequency of the photonic resonance, which is proportional to its linewidth^17^.

Such phenomenological models are now being challenged in a variety of emerging regimes of light-matter interaction, especially with nanophotonic systems, where increased strength of the system coupling parameters and tightly confined (effective) mode volumes probe system-environment interactions over a large spectral range, which we refer to as *broadband* dissipative cavity-QED.

One striking example of broadband dissipative cavity-QED is the case of ultrastrong coupling (USC)^18,19^. The USC regime of dipole-cavity coupling occurs when a rotating-wave approximation (RWA) is no longer valid, occurring roughly when the light-matter coupling strength is within an order of magnitude of the bare system frequencies, and the ground state of the hybridized system becomes an entangled state of photons and matter. Studies of USC are not only driven by fundamental considerations, but by emerging experiments, including intersubband polaritons^20–22^, circuit-QED ^18,19,23^, Landau polaritons^24–27^, and plasmonic resonators^28–31^. In such extreme light-matter coupling regimes, beyond the breakdown of the RWA (which can be addressed by generalizing the Jaynes-Cummings model to the Quantum Rabi Model), another issue is that naive truncation of the matter degrees of freedom to a two-level system (TLS), as is commonly done, can introduce spurious gauge dependence and thus incorrect predictions. This problem has been addressed by introducing a generalized minimal coupling replacement^32,33^.

While much progress has been made in the generalization of light-matter models on the *system* level, a rigorous and efficient few-mode treatment of loss and dissipation in such coupling regimes is still an open question. Previous works have typically employed a *normal mode* expansion with real eigenfrequencies, where photon decay is added phenomenologically as a Lindblad dissipator or by coupling the normal modes to supplementary photon reservoirs. While some progress has been made toward an ab initio approach in 1D^34–36^ and dispersionless^37–39^ systems, a tractable approach to realistic 3D cavity structures including potential material dispersion and absorption that remains valid in USC has not yet been found.

A critical problem lies in the fact that reliance on phenomenology has been shown recently to be insufficient to accurately predict spectral observables from systems in the USC regime^33,40,41^. The need for ab initio dissipative few-mode models has also been highlighted recently in other contexts in quantum optics^38,42^. We have recently made a significant advance in this problem by identifying, using a semi-phenomenological approach, the correct form of the spectral density for a single lossy quantized cavity mode^17^, which can be used to *accurately* calculate spectral observables in broadband coupling regimes, including USC, when the system is described by a sufficiently isolated single optical resonance.

The result in Ref. ^17^ employs a *quasinormal mode* (QNM) approach ^43–46^. The QNMs ${\tilde{{\bf{f}}}}_{\mu }({\bf{r}})$ are *complex* solutions to the Helmholtz equation,

1 $${\boldsymbol{\nabla }}\times {\boldsymbol{\nabla }}\times {\tilde{{\bf{f}}}}_{\mu }({\bf{r}})-\frac{{\tilde{\omega }}_{\mu }^{2}}{{c}^{2}}\epsilon ({\bf{r}},{\tilde{\omega }}_{\mu }){\tilde{{\bf{f}}}}_{\mu }({\bf{r}})=0$$

which is solved with suitable radiation conditions. As a consequence of the open boundary conditions and possibly also material absorption, the corresponding QNM eigenfrequencies ${\tilde{\omega }}_{\mu }={\omega }_{\mu }-i{\kappa }_{\mu }/2$ are also complex-valued. While the real part *ω*_*μ*_ describes the resonance of the QNMs, the (negative) imaginary part *κ*_*μ*_/2 reflects the width of the resonance (dissipation). Notably, Ref. ^17^ finds that the phase of the QNM at the dipole location in cavity-QED setups, $\,\mathrm{arg}\,\{\tilde{{\bf{f}}}\}$, is crucial to the underlying spectral density of the cavity-reservoir interaction. However, Ref. ^17^ employed an essentially phenomenological approach, and it remains an open question if this result can be deduced from a rigorous quantum formalism.

In this work, we derive a fully ab initio single-mode quantum theory of dissipative cavity-QED, using the formalism of quantized QNMs, which can be used to accurately model the USC and broadband dissipative regimes, and predict substantial corrections beyond existing phenomenological approaches. The theory of quantized QNMs allows one to construct proper QNM photon Fock states^47,48^ for open/absorptive cavities, and has found successful application in studying various quantum optical processes^47,49–57^

Our quantized QNM approach allows, for the first time to our knowledge, an ab initio quantum theory of dissipative cavity-QED which is capable of recovering these recent results for the spectral density of a lossy cavity mode^17^, while still retaining the full convenience of a single-mode ME approach which most effectively captures the physics associated with isolated discrete photonic resonances and allows for direct access to their observables. Our approach contrasts with other methods including pseudomode^41,58–60^ and hierarchical equations of motion (HEOM)^61–65^ approaches, which, while powerful and not relying on a Markov approximation, often require the introduction of many more modes than physically present in the system (or auxiliary density matrices in the HEOM method), and which to date often have been benchmarked with phenomenological cavity spectral densities. Access to photon observables can also be more challenging with these methods.

From our theoretical framework, we find that when the product of the cavity quality (Q) factor and the QNM phase at the dipole location becomes sufficiently large, the dissipative cavity-QED system enters a *broadband* dissipative regime, where phenomenological models break down, and our approach allows for accurate quantitative modeling of spectral observables. The broadband dissipative regime includes not only USC, but also weak and strong coupling regimes with sufficiently large detunings between the cavity and dipole^17^, and, surprisingly, even on resonance can often be reached in dielectric cavities with coupling strengths orders of magnitude below USC. We also find that the product of the QNM phase and Q-factor sets intrinsic limits of validity to single-mode models, and thus, our approach allows for important insights into the extent of validity of such widely-adopted models in broadband dissipative regimes, including but not limited to USC.

We furthermore advance previously existing theories of quantized QNMs by presenting them as a direct partitioning of the Hilbert space of the entire complex medium (resonator structure + environment)^66^, using the rigorous theory of gauge-invariant macroscopic QED^67^, and identify a new “spatially specified” quantization representation of the QNMs, which we find necessary to recover known results. This work *significantly generalizes previous single-mode quantum models in the USC regime for open cavity systems*, in that it allows one to access the coupling constants and cavity-environment spectral density from the QNM and the geometry properties of the scattering structure *without any phenomenological fitting parameters*. Our results are relevant to emerging experiments, and we predict they should in principle be observable in the near future.

The structure of the rest of the paper is as follows: in “Theoretical Approach”, we briefly describe the theoretical components of our ab initio approach to broadband dissipative cavity-QED, including the macroscopic QED framework for quantization of electromagnetic fields in arbitrary media, our projection approach to QNM quantization and its “spatially specified” representation in the single-mode regime, the gauge-invariant light-matter coupling formalism, and the resulting ab initio ME, valid in the broadband dissipative regime (including USC). We then use this ME theory to introduce and define the broadband dissipative regime of cavity-QED in terms of the dimensionless parameter, ${\tilde{\Omega }}_{{\rm{BB}}}$, which is the value that a system dynamical rate (e.g., the cavity-dipole coupling strength) divided by the cavity frequency must take to enter the regime where broadband dissipative corrections become significant.

In “Results and Simulations for Various Cavity Designs Under Weak-Coupling”, we perform detailed EM simulations for a variety of realistic dielectric and plasmonic cavity designs, verifying our quantized QNM theory for the weak dipole-cavity coupling regime where comparison with perturbative results is possible, showing how broadband dissipative corrections can occur even at times well below the USC threshold, and estimating limits to validity of single-mode dissipative models. In “Quantum ME Simulations in the USC Regime”, we perform quantum ME simulations in the USC regime, showing the strong impact of our ab initio theory’s corrections when compared with heuristic models. In “Experimental Prospects”, we connect to current and emerging experiments involving cavity designs with ultrasmall mode volumes, both dielectric and plasmonic, and discuss prospects for near-term experimental observations of the predicted broadband dissipative dynamics. Finally, in “Conclusions”, we conclude and summarize the main findings of the work. We also include a Supplementary Information (See Supplementary Information for more detailed information, where we also cite references^68–91^), which includes detailed derivations and generalizations of our ab initio ME and quantized QNM formalism, as well as additional resonator calculations.

## Results

### Theoretical approach

In this section, we present a brief overview of our theoretical approach; detailed derivations, a list of major approximations made, and further formalism can be found in the Supplementary Information.

#### Macroscopic QED and quantized QNM formalism

To describe the quantized EM fields in arbitrary dielectric 3D media, including potential dispersion and absorption, we use the theory of macroscopic QED^67,92–94^. The central object in our formalism is the transverse vector potential

2 $${\hat{{\bf{A}}}}_{\perp }({\bf{r}})=\frac{1}{{\epsilon }_{0}}\int\,\frac{d\omega }{{\omega }^{2}}\int{d}^{3}s\,{{\bf{G}}}^{\perp }({\bf{r}},{\bf{s}},\omega )\cdot {\hat{{\bf{j}}}}_{{\rm{N}}}({\bf{s}},\omega )$$

where ${\hat{{\bf{j}}}}_{{\rm{N}}}({\bf{s}},\omega )=\omega \sqrt{\frac{\hslash {\epsilon }_{0}{\epsilon }_{I}({\bf{r}},\omega )}{\pi }}\hat{{\bf{b}}}({\bf{r}},\omega )+\,\mathrm{H.c.}\,$ is a noise polarization defined in terms of the (possibly fictitious) imaginary component of the medium (cavity + environment) dielectric function *ϵ*_*I*_(**r**, *ω*), medium polariton operators which satisfy $[\hat{{\bf{b}}}({\bf{r}},\omega ),{\hat{{\bf{b}}}}^{\dagger }({{\bf{r}}}^{{\prime} },\omega {\prime} )]=\delta ({\bf{r}}-{{\bf{r}}}^{{\prime} })\delta (\omega -\omega {\prime} ){\bf{I}}$, and the medium transverse Green function ${{\bf{G}}}^{\perp }({\bf{r}},{{\bf{r}}}^{{\prime} },\omega )$, which satisfies a radiation boundary condition.

**Fig. 1:**
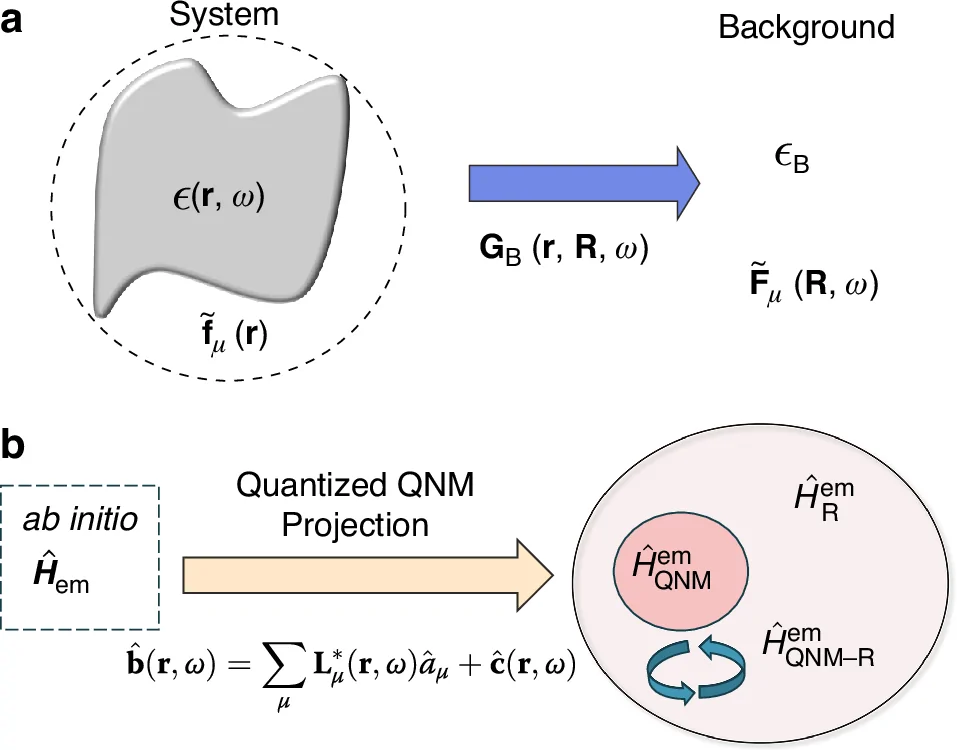
Schematic of ab initio quantized QNM formalism and system-reservoir construction. **a** Visualization of the field expansion in terms of QNMs and background contributions. The *system* region near the scattering geometry is represented by the discrete set of QNMs ${\tilde{{\bf{f}}}}_{\mu }({\bf{r}})$. The *background* region with Green function **G**_B_ and dielectric constant *ϵ*_B_ is described by regularized QNMs ${\tilde{{\bf{F}}}}_{\mu }({\bf{R}},\omega )$. The field operators, e.g., ${\hat{{\bf{A}}}}_{\perp }({\bf{r}})$, are formed by an integration of the total photon Green function together with the polariton noise operators over all real space. **b** Illustration of quantized QNM projection approach to ab initio system-reservoir theory

To exploit the discrete photonic resonances in cavity structures, we express the transverse Green function in terms of QNMs, defined by Eq. (1). In particular, at locations **r** near the cavity (the “system” region),

3 $${{\bf{G}}}^{\perp }({\bf{r}},{{\bf{r}}}^{{\prime} },\omega )=\sum _{\mu }{A}_{\mu }(\omega ){\tilde{{\bf{f}}}}_{\mu }({\bf{r}}){\tilde{{\bf{F}}}}_{\mu }^{{\prime} }({{\bf{r}}}^{{\prime} },\omega )$$

where ${A}_{\mu }(\omega )=\omega /(2({\tilde{\omega }}_{\mu }-\omega ))$ is a frequency-dependent QNM expansion coefficient, and ${{\bf{F}}}_{\mu }^{{\prime} }({{\bf{r}}}^{{\prime} },\omega )$ is equal to ${{\bf{f}}}_{\mu }({{\bf{r}}}^{{\prime} })$ when ${{\bf{r}}}^{{\prime} }$ is near the cavity geometry, and takes on a regularized form for far-field positions from the cavity, characterized by a (e.g.,) Dyson equation form capturing propagation from the cavity through the background medium Green function, illustrated by Fig. 1(a). For simplicity, we neglect background terms (which give rise to scattering into non-cavity channels when interfacing with matter, as well as cross-terms which can give rise to Fano-interference^95^) in this work, particularly as these would typically be very small in USC.

While the classical EM information of the cavity is incorporated through the QNM Green function expansion, the quantum oscillator nature of the cavity resonances can be captured using a recently proposed projection approach^66,67^. We write the polariton operators as

4 $$\hat{{\bf{b}}}({\bf{r}},\omega )=\sum _{\mu }{{\bf{L}}}_{\mu }^{* }({\bf{r}},\omega ){\hat{a}}_{\mu }+\hat{{\bf{c}}}({\bf{r}},\omega )$$

where ${\hat{a}}_{\mu }$ corresponds to quantized QNMs, and $\hat{{\bf{c}}}({\bf{r}},\omega )$ corresponds to a residual *reservoir* component. These reservoir operators have non-local commutation relations, but within the Markov approximation can be treated as local, which is shown in^66^ and the Supplementary Information. Through appropriate choice of the QNM projection functions **L**_*μ*_(**r**, *ω*), the transverse vector potential can be written as ${\hat{{\bf{A}}}}_{{\rm{QNM}}}^{\perp }({\bf{r}})={\sum }_{\mu }\sqrt{\frac{\hslash }{2{\epsilon }_{0}{\omega }_{\mu }}}{\tilde{{\bf{f}}}}_{\mu }^{{\rm{s}}}({\bf{r}}){\hat{a}}_{\mu }+\,\mathrm{H.c.}\,$, where ${\tilde{{\bf{f}}}}_{\mu }^{{\rm{s}}}({\bf{r}})={\sum }_{\nu }{\tilde{{\bf{f}}}}_{\nu }({\bf{r}}){\left[{S}^{\frac{1}{2}}\right]}_{\nu \mu }$ is a symmetrized QNM, defined in terms of the symmetrization matrix *S*_*μν*_, which can be calculated in in terms of integrals over the regularized QNMs, and also gives information regarding the relative proportion of radiative vs. non-radiative emission. We call this representation of the symmetrization matrix the “spatially unspecified” representation, as it does not pick out any specific value of position and contrasts with the spatially specified representation we introduce later. The fact that the vector potential can be expressed entirely in terms of the QNM subspace is a consequence of the choice of definition of the projection functions **L**_*μ*_(**r**, *ω*) in our formalism (which leads to slightly different definitions of the quantized QNMs than those used before^47^) and neglect of background radiation terms; see the Supplementary Information for details.

This formalism allows us to construct an ab initio system-reservoir theory for loss from the quantized cavity modes. By expressing the EM-polariton Hamiltonian ${\hat{H}}_{{\rm{em}}}=\int\,{d}^{3}r\int\,d\omega \hslash \omega {\hat{{\bf{b}}}}^{\dagger }({\bf{r}},\omega )\cdot \hat{{\bf{b}}}({\bf{r}},\omega )$ in terms of the projection in Eq. (4), we naturally build up a Hamiltonian with cavity system component ${\hat{H}}_{{\rm{QNM}}}^{{\rm{em}}}={\sum }_{\mu \nu }\hslash {\chi }_{\mu \nu }{\hat{a}}_{\mu }^{\dagger }{\hat{a}}_{\nu }$ (with *χ*_*μμ*_ ≈ *ω*_*μ*_), reservoir component ${\hat{H}}_{{\rm{R}}}^{{\rm{em}}}=\int\,{d}^{3}r\int\,d\omega \hslash \omega {\hat{{\bf{c}}}}^{\dagger }({\bf{r}},\omega )\cdot \hat{{\bf{c}}}({\bf{r}},\omega )$, and a term governing the coupling of the cavity to its photon reservoir, which gives rise to cavity loss:

5 $$\begin{array}{ll}{H}_{{\rm{QNM-R}}}^{{\rm{em}}}\,=\,\hslash \sum\limits_{\mu }\int_{0}^{\infty }d\omega \int\,{d}^{3}r\,{{\bf{g}}}_{\mu }({\bf{r}},\omega )\cdot \hat{{\bf{c}}}({\bf{r}},\omega ){\hat{a}}_{\mu }^{\dagger }+{\rm{H.c.}}\end{array}$$

where **g**_*μ*_(**r**, *ω*) and *χ*_*μν*_ are defined in terms of the QNM projection functions. Fig. 1b shows an illustration of the approach.

In the single-mode case (taking *μ* = *c*), we can also move to a “spatially specified” form of the *S*_*c*_ = *S*_*cc*_ quantization parameter. In this representation, we find (see Supplementary Information)

6 $${S}_{c}\to {S}_{c}({\bf{n}},{{\bf{r}}}_{0})\approx \cos (2{\phi }_{0})$$

where **r**_0_ and **n** are a position coordinate and unit vector, which we choose to coincide with the position of the dipole and its dipole moment orientation, for which we introduce coupling to in the following subsection. It is important to note that, in this representation, *S*_*c*_(**n**, **r**_0_) *depends explicitly on the position of the QNM that has been specified*, and as such, will generally give different results than the spatially unspecified form. The two forms are different ways one can choose to make a single-mode approximation, from the initial multimode *ab initio construction*. The spatially specified representation arises from imposing a relation that holds for general Greens functions to also hold within the approximation, where we assume the Greens function of the entire system can be accurately represented with just a single QNM. While the spatially unspecified representation has previously been used to explain many effects outside of the broadband dissipative regime^47,49–57^, the spatially specified representation is required to identify the correct frequency- and QNM phase- dependent decay rates in the USC regime^17^, as well as more generally at spatial positions where the QNM phase is substantial^96^, even with weak coupling.

#### Broadband light-matter coupling

In recent years, it has become well-established that the usual approach to truncate the minimal coupling Hamiltonian giving light-matter interactions can lead to inconsistent gauge-dependent predictions when in broadband regimes (including USC), and that gauge-invariance can be restored by means of an appropriate generalization of the minimal coupling procedure^32,33,67,97–100^. In this subsection, we introduce gauge-invariant light-matter coupling to a dipole TLS, following the formalism of Ref. ^67^. To keep our results general, we do not treat the matter degrees of freedom on a similar ab initio basis, given that this is a separate problem and of course depends on the specific material system of interest. We work here and throughout with the Coulomb gauge, though we also show results for the dipole gauge in the Supplementary Information.

Before introducing transverse light-matter coupling, we assume we have a Hamiltonian ${\hat{H}}_{{\rm{bare}}}={\hat{H}}_{0}+{\hat{H}}_{{\rm{em}}}$, where ${\hat{H}}_{{\rm{em}}}$ follows the decomposition in the previous subsection, and ${\hat{H}}_{0}$ is an effective matter Hamiltonian corresponding to an optically-active dipole which can be represented in a two-level basis ($\left\vert g\right\rangle ,\left\vert e\right\rangle$) as ${\hat{H}}_{0}=\frac{\hslash {\omega }_{0}}{2}{\hat{\sigma }}_{z}$, where *ℏω*_0_ is the two-level energy separation. For the Coulomb gauge, the total Hamiltonian is ${\hat{{\mathcal{H}}}}_{{\rm{C}}}={\hat{H}}_{{\rm{em}}}+\hat{{\mathcal{W}}}{\hat{{\mathcal{H}}}}_{0}{\hat{{\mathcal{W}}}}^{\dagger }$, where $\hat{{\mathcal{W}}}=\,{\mathrm{exp}}\,\left[\frac{i}{\hslash }{\bf{d}}\cdot {\hat{{\bf{A}}}}_{\perp }({\bf{0}}){\hat{\sigma }}_{x}\right]$, with **d** the (assumed real) transition dipole moment of the TLS, and we let the position of the dipole here and throughout to be at **r** = **0**. Here we consider only transverse interactions, but longitudinal field couplings can also be included^101^. As evidenced by our simulations in “Results and Simulations for Various Cavity Designs Under Weak-Coupling”, the cavities we study are dominated by the transverse mode contribution, which can also hold for ultra-small gap cavities, and even when non-local effects become important^102^. Expanding the exponential in $\hat{{\mathcal{W}}}$ and separating the Hamiltonian into system, reservoir, and system-reservoir terms, we obtain the total Hamiltonian for a single-mode system ${\hat{{\mathcal{H}}}}_{{\rm{C}}}={\hat{{\mathcal{H}}}}_{{\rm{C}}}^{{\rm{S}}}+{\hat{H}}_{{\rm{R}}}^{{\rm{em}}}+{\hat{H}}_{{\rm{QNM-R}}}^{{\rm{em}}}$, where the system Hamiltonian is

7 $${\hat{{\mathcal{H}}}}_{{\rm{C}}}^{{\rm{S}}}={\hat{H}}_{{\rm{QNM}}}^{{\rm{em}}}+\frac{\hslash {\omega }_{0}}{2}\left[\cos (\hat{\Phi }){\hat{\sigma }}_{z}+\sin (\hat{\Phi }){\hat{\sigma }}_{y}\right]$$

with ${\hat{H}}_{{\rm{QNM}}}^{{\rm{em}}}=\hslash {\omega }_{c}{\hat{a}}_{c}^{\dagger }{\hat{a}}_{c}$, and $\hat{\Phi }=2({\eta }_{c}{\hat{a}}_{c}+{\eta }_{c}^{* }{\hat{a}}_{c}^{\dagger })$, where

8 $${\eta }_{c}=\frac{{\bf{d}}\cdot {\tilde{{\bf{f}}}}_{c}^{{\rm{s}}}({\bf{0}})}{\sqrt{2\hslash \epsilon {\omega }_{c}}}$$

is a normalized cavity-dipole coupling constant, and ${\hat{H}}_{{\rm{R}}}^{{\rm{em}}}$ and ${\hat{H}}_{{\rm{QNM-R}}}^{{\rm{em}}}$ are the reservoir and QNM-reservoir coupling Hamiltonians defined in the previous subsection. From this ab initio system-reservoir Hamiltonian ${\hat{{\mathcal{H}}}}_{{\rm{C}}}$, an ME can be derived for the cavity photon loss, valid in the broadband dissipative and USC regimes.

#### Ab initio ME for broadband dissipative regime

From the system-reservoir Hamiltonian ${\hat{{\mathcal{H}}}}_{{\rm{C}}}$, we first introduce the dressed-state basis $\{\left\vert j\right\rangle \}$ in which the TLS-QNM system Hamiltonian is ${\hat{{\mathcal{H}}}}_{{\rm{C}}}^{{\rm{S}}}=\hslash {\sum }_{j}{\omega }_{j}\left\vert j\right\rangle \left\langle j\right\vert ,$ where *ℏω*_*j*_ are the associated eigenenergies of the dressed states. This diagonal form allows for the interaction picture transformation to be trivially performed, which is necessary to derive the second-order Born-Markov ME. Under this formalism^11^, with the standard assumptions^33^, the ME is (neglecting photonic Lamb shifts)

9 $$\dot{\rho }=-\frac{i}{\hslash }[{\hat{{\mathcal{H}}}}_{{\rm{C}}}^{{\rm{S}}},\rho ]+\sum _{\alpha }\frac{{\Gamma }_{\alpha }}{2}{\mathcal{L}}[{\hat{\sigma }}_{\alpha }]\rho$$

where we have introduced the notation where *α* = (*j*, *k*) indexes a TLS-QNM system eigenstate transition with *ℏω*_*α*_ = *ℏω*_*k*_ − *ℏω*_*j*_ > 0, and ${\hat{\sigma }}_{\alpha }=\left\vert j\right\rangle \left\langle k\right\vert$, and we have employed the secular approximation for simplicity (valid for strong coupling—i.e., well-separated spectral peaks—but not necessary, and we forgo this approximation in “Quantum ME Simulations in the USC Regime”), which allows the dissipator component to be expressed in terms of the Lindblad superoperator ${\mathcal{L}}[{\hat{\sigma }}_{\alpha }]\rho =2{\hat{\sigma }}_{\alpha }\rho {\hat{\sigma }}_{\alpha }^{\dagger }-\{{\hat{\sigma }}_{\alpha }^{\dagger }{\hat{\sigma }}_{\alpha },\rho \}$.

The decay rates Γ_*α*_ can be calculated directly from ${\hat{{\mathcal{H}}}}_{{\rm{C}}}$ in the Born-Markov ME formalism, but as ${\hat{{\mathcal{H}}}}_{{\rm{QNM-R}}}^{{\rm{em}}}$ has been written in the spatially unspecified representation, the resultant decay rates do not directly depend on the phase of the QNMs at the dipole location and thus do not reproduce known results^17^. To rectify this, we move to a spatially-specified representation, as in “Macroscopic QED and quantized QNM formalism” (and the Supplementary Information), where we find

10 $${\Gamma }_{\alpha }={\kappa }_{c}| {c}_{\alpha }^{c}{| }^{2}\frac{{\omega }_{c}}{{\omega }_{\alpha }}{\zeta }_{c}({\phi }_{0},{\omega }_{\alpha }),$$

where $${c}_{\alpha }^{c}=\left\langle j\right\vert {\hat{a}}_{c}\left\vert k\right\rangle$$, and

11 $${\zeta }_{c}({\phi }_{0},\omega )=1-2{Q}_{c}\tan (2{\phi }_{0})\left(\frac{\omega }{{\omega }_{c}}-1\right)$$

is a factor which depends on the position of the dipole through the projected QNM phase ${\phi }_{0}=\,\mathrm{arg}\,\{\frac{{\bf{d}}}{| {\bf{d}}| }\cdot {\tilde{{\bf{f}}}}_{c}({\bf{0}})\}$, and quantifies the deviation of the Lorentzian (modulated by a linear frequency factor) lineshape of TLS emission in the weak-coupling regime. The cavity quality factor is *Q*_*c*_ = *ω*_*c*_/*κ*_*c*_. This spectral density recovers previous results and confirms the gauge-dependence of the spectral density when the coupling between the cavity operators and their environment is expressed in the Coulomb gauge as having an overall scaling ~*ω*^−1^, which was previously found^17^. If the coupling between the cavity operators and their reservoir is expressed in the dipole gauge, this scaling is instead ~*ω*, which we confirm in the Supplementary Information by using a different definition of the quantized QNMs in terms of an electric field expansion (as opposed to the vector potential expansion here). To the best of our knowledge, this is the *first time* the correct form of the cavity system-reservoir coupling has been derived from an ab initio perspective valid for general 3D resonators.

To ensure physical results, a bound on the validity of single-mode models can be found by requiring *ζ*_*c*_(*ϕ*_0_, *ω*_*α*_) > 0; using (for one example) a simple on-resonance model where *ω*_0_ = *ω*_*c*_, and assuming *ω*_*α*_ ~ *ω*_*c*_(1 + ∣*η*_*c*_∣) (i.e., to leading order in ∣*η*_*c*_∣), we find, for the single-mode approximation to remain accurate, as an *upper bound*, $| {\eta }_{c}| < | {\eta }_{c}^{(1)}|$, where

12 $$| {\eta }_{c}^{(1)}| =\frac{1}{| 2{Q}_{c}\tan \left(2{\phi }_{0}\right)| }$$

This is only a heuristic expression, and becomes less quantitatively accurate when higher-order (in *η*_*c*_) contributions to the transition *ω*_*α*_ are significant, as well as when the cavity and TLS are detuned, but nonetheless provides a useful rough analytic heuristic to determine approximate rough bounds of single-modedness. It also highlights an intrinsic function of the QNM phase as a limiting factor in the approximation of single-mode models under broadband coupling regimes. Indeed, in “Results and Simulations for Various Cavity Designs Under Weak-Coupling”, we find that in practice the single-mode QNM theory breaks down (in terms of providing accurate corrections to phenomenological models) well below this threshold. We stress that this approximate bound on the normalized cavity-TLS coupling ∣*η*_*c*_∣ can easily be generalized to *any* dynamical rate, as the requirement for a positive spectral density is really a bound on *ω*_*α*_ − *ω*_*c*_; for example, with a detuning (and still to leading order in ∣*η*_*c*_∣), the bound would apply to $\sqrt{{({\omega }_{0}-{\omega }_{c})}^{2}/{\omega }_{c}^{2}+| {\eta }_{c}{| }^{2}}$. We also note that as our ME relies on a Markov approximation, it may break down for cavities with extremely small Q factors (≲5 ^41^), where the representation of the mode as a single quantum harmonic oscillator may become questionable.

#### Definition of broadband dissipative regime of cavity-QED

We can now posit a definition of the broadband regime of dissipative cavity-QED. Even *outside of the USC regime*, corrections to phenomenological lossy cavity ME models based on our ab initio approach will be highly perceptible for cavities operating in a regime with sufficiently large values of $2{Q}_{c}\tan 2{\phi }_{0}$.

For a system with a characteristic dynamical rate *Ω* which satisfies Ω/*ω*_*c*_ ≪ 1, this “broadband” regime emerges when Ω/*ω*_*c*_ becomes substantial relative to ${[2{Q}_{c}\tan 2{\phi }_{0}]}^{-1}$. To put this more precisely, we can use the analytical solution for the spectral linewidths of the dominant two peaks of a resonantly-coupled (*ω*_0_ = *ω*_*c*_) TLS-cavity system for weak ∣*η*_*c*_∣ (derived in the Supplementary Information):

13 $${\Gamma }_{\pm }=\frac{{\kappa }_{c}}{2}\left[1\pm \frac{| {\eta }_{c}| }{2}\left(1-4{Q}_{c}\tan (2{\phi }_{0})\right)\right]$$

where ‘ + ’ denotes the higher-energy (blue) peak and ‘ − ’ denotes the lower-energy (red) peak. In analogy with the typical definition of the USC regime as ∣*η*_*c*_∣ > 0.1, we thus define the “broadband” regime of (single-mode) lossy cavity-QED as $| {\eta }_{c}| \ge {\tilde{\Omega }}_{{\rm{BB}}}$, where ${\tilde{\Omega }}_{{\rm{BB}}}$ is defined as

14 $${\widetilde{{\rm\Omega }}}_{\mathrm{BB}}=0.1\times \,{\mathrm{min}}\,\{1,|1-4{Q}_{c}{\rm{tan }}(2{\phi }_{0}){|}^{-1}\}$$

In more general terms, this criterion should also hold whenever any characteristic dynamical rate of the system divided by *ω*_*c*_ approaches ${\tilde{\Omega }}_{{\rm{BB}}}$.

It should be noted that this definition is only a rough estimate for when broadband dissipative effects become substantial, and perceptible modifications to (e.g.,) cavity spectra can appear below this threshold as well, which we indeed show in “Quantum ME Simulations in the USC Regime”.

### Results and simulations for various cavity designs under weak-coupling

In this section, we show specific examples of both plasmonic and dielectric cavity designs. In addition to performing modal simulations to identify dominant (and higher-order) QNM contributions to the transverse coupling of the dipole, we also compare with full non-modal Maxwell simulations to verify the accuracy of our theory, which are in principle numerically exact. We also show additional resonator examples in the Supplementary Information.

As an observable to assess the validity of our approach, here we use the Purcell-enhanced decay rate of a *weakly-coupled* single dipole, similar to the approach of Ref. ^17^. This allows us to employ the perturbative result for the dipole decay rate

15 $$\gamma ({\omega }_{0})=\frac{2}{{\epsilon }_{0}}{\bf{d}}\cdot \,\mathrm{Im}\,\{{{\bf{G}}}_{\perp }({\bf{0}},{\bf{0}},{\omega }_{0})\}\cdot {\bf{d}}$$

expressed as a function of the TLS frequency. Under a single-QNM expansion for the transverse Green function, this rate becomes

16 $${\gamma }_{{\rm{QNM}}}({\omega }_{0})=\frac{4| {\tilde{g}}_{{\rm{c}}}^{{\rm{d}}}{| }^{2}}{{\kappa }_{c}}\frac{{\omega }_{0}}{{\omega }_{c}}\frac{{\kappa }_{c}^{2}/4}{{\kappa }_{c}^{2}/4+{({\omega }_{0}-{\omega }_{c})}^{2}}{\zeta }_{c}({\phi }_{0},{\omega }_{0})$$

expressed in terms of the dipole gauge TLS-cavity coupling ${\tilde{g}}_{c}^{{\rm{d}}}={\omega }_{c}{\eta }_{c}$, which is independent of dipole frequency. Equation (16) can also be derived from the quantized QNM theory we present in this work (without making the secular approximation, for this weak-coupling case). After confirming the validity of our quantized QNM theory in the weak-coupling regime, we can use it to perform quantum simulations in the USC regime, as we do in “Quantum ME Simulations in the USC Regime”.

To compare the result of Eq. (16) for a variety of cavity designs with the general expression in Eq. (15) with the full non-modal Green function found from solving Maxwell’s equations, it is useful to normalize our results to the Lorentzian function ${L}_{c}(\omega )={\gamma }_{{\rm{QNM}}}({\omega }_{c})\frac{{\kappa }_{c}^{2}/4}{{\kappa }_{c}^{2}/4+{(\omega -{\omega }_{c})}^{2}}$. The frequency dependence of *L*_*c*_(*ω*_0_) is precisely what is obtained by phenomenological models of loss in quantized cavity systems (i.e., with a simple Lindblad term with rate *κ*_*c*_ and collapse operator ${\hat{a}}_{c}$), and as such, substantial deviations in *γ*(*ω*_0_)/*L*_*c*_(*ω*_0_) from unity are indicative of the broadband dissipative regime of cavity-QED. For full dipole simulation results, we subtract off a slowly-varying background component, proportional to the free-space decay rate scaled by a constant fitting factor, to isolate the QNM contribution from the decay from the TLS directly into non-resonant modes, which has been neglected in this work for simplicity of presentation. Note that for some resonators (particularly those supporting sufficiently large Purcell factors) this subtraction is not needed to see good agreement due to the domination of the QNM decay over the residual background coupling (e.g., those in Ref. ^17^, where no subtraction was implemented).

We summarize the relevant QNM parameters for each of the dominant modes of the cavity designs in Table 1, where we also calculate the approximate criterion from Eq. (12) for an upper-bound to limits of the single-mode model, by means of the parameter $\left\vert {\eta }_{c}^{{\rm{(1)}}}| ={\left[2{Q}_{c}\tan 2{\phi }_{0}\right\vert }^{-1}\right.$. We also show the parameter ${\tilde{\Omega }}_{{\rm{BB}}}$ as an estimate of the value of ∣*η*_*c*_∣ above which broadband dissipative effects giving corrections beyond the usual phenomenological models become substantial, as discussed in “Definition of broadband dissipative regime of cavity-QED”. For all examples, we consider the resonator structure to be placed in free space (*n*_B_ = 1). We also, in some instances, investigate the contribution of higher modes by means of a multimode generalization of Eq. (16), where a sum runs over the QNM mode indices^17^. In this case, *μ* = *c* = 1 corresponds to the dominant QNM, with *μ* > 1 corresponding to subdominant modes.

**Table 1:** Parameters of dominant QNM for multiple cavity structures

	Ellipsoid Dimer	Bowtie	2D PC
$\hslash {\tilde{\omega }}_{c}$ (eV)	2.620−0.04996*i*	0.7987−0.002507*i*	1.957−6.319 × 10^−4^*i*
*Q*_*c*_	26.22	159.3	1549
${\tilde{f}}_{z}$	2.670 × 10^11^	2.235 × 10^9^	3.302 × 10^6^
	+ 2.553 × 10^9^*i* ${{\rm{m}}}^{-\frac{3}{2}}$	+ 7.915 × 10^6^*i* ${{\rm{m}}}^{-\frac{3}{2}}$	− 7.178 × 10^3^*i* m^−1^
$\tan (2{\phi }_{0})\approx 2{\phi }_{0}$	0.0191	0.00708	− 0.00435
$| {\eta }_{c}^{(1)}|$	1.0	0.44	0.074
${\tilde{\Omega }}_{{\rm{BB}}}$	0.1	0.028	0.0036

The QNM profile ${\tilde{f}}_{z}$ values are the dominant components of the vector ${\tilde{{\bf{f}}}}_{c}({\bf{0}})$

The results of the following subsections suggest five important findings which we posit as *potential* general rules-of-thumb for cavity structures which support a dominant mode in a relevant optical frequency window of interest: (i) for plasmonic resonators, the USC and broadband dissipative regimes typically coincide, and our ab initio dissipative single-mode QNM theory can accurately be used to model dissipative dynamics beyond the predictions of phenomenological models. (ii) For dielectric resonator designs (excluding 1D or quasi-1D cavities), the broadband dissipative regime is reached with dynamical rates often orders of magnitude below the USC threshold, and our approach can sometimes be used to identify single-mode corrections beyond phenomenological models, depending on the cavity design. (iii) For dielectric cavities, USC dynamics span a bandwidth far beyond the regime where the single-QNM calculation agrees with full Maxwell simulations, and thus we do not expect single-mode models to be fully accurate in this regime. (iv) In 1D or quasi-1D cavity designs, the quasi-harmonic distribution of longitudinal modes likely precludes the possibility of accurate single-mode calculations in the broadband dissipative regime. (v) In all cases, full Maxwell simulations begin to deviate with the QNM theory predictions at a bandwidth smaller than that suggested by $| {\eta }_{c}^{(1)}|.$

#### Plasmonic dimer resonator

**Fig. 2:**
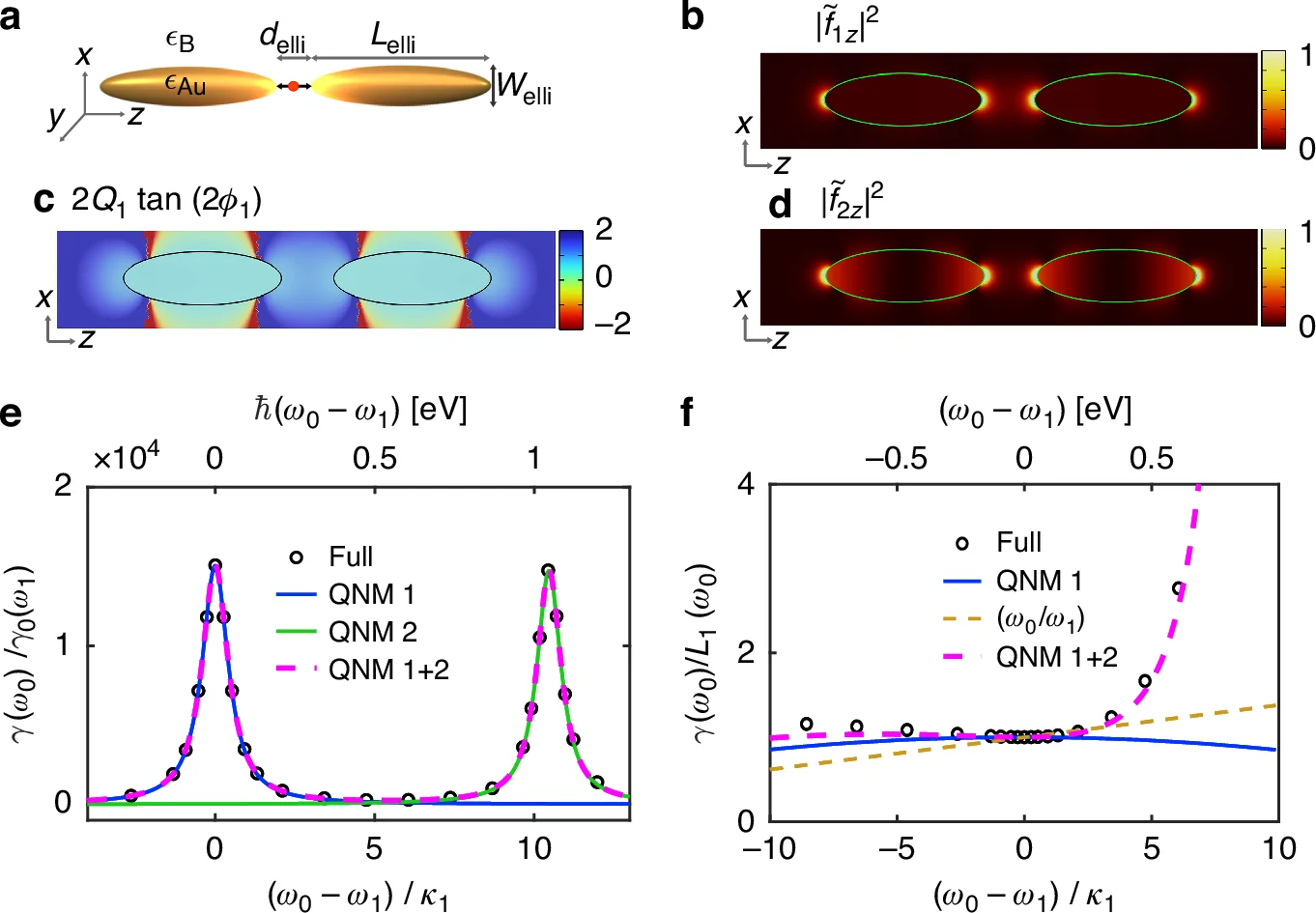
Results for gold dimer cavity. **a** Schematic of gold-like ellipsoid dimer in free space (*ϵ*_B_ = 1.0) with dimensions *W*_elli_ = 10 nm, *L*_elli_ = 30 nm, *d*_elli_ = 10 nm. QNM profile for: **b** dominant QNM (1) and **d** second QNM (2) (arb. units). **c** Phase distribution of $2{Q}_{{\rm{1}}}\tan (2{\phi }_{1})$ for dominant QNM, where the phase is defined from ${\tilde{f}}_{1z}({\bf{r}})=| {\tilde{f}}_{1z}({\bf{r}})| {e}^{i{\phi }_{1}({\bf{r}})}$. **e** Purcell factors for a *z* − polarized emitter at the gap center, normalized by the free-space rate *γ*_0_(*ω*) = ∣**d**∣^2^*ω*^3^/(3*πϵ*_0_*ℏc*^3^). **f** The decay rate normalized by Lorentzian function *L*_1_ = *L*_*c*_. Here, we plot the full dipole decay rate with the background *γ*_0_(*ω*_0_) subtracted out. The dominant QNM (1) parameters are found in Table 1, and QNM 2 has (complex) eigenfrequency $\hslash {\tilde{\omega }}_{2}=(3.667-0.04602i)$ eV and projected phase at the dipole location *ϕ*_2_(**r**_0_) = 0.00616

We first consider a gold-like dimer [Fig. 2a] consisting of two identical ellipsoidal nanorods^47,103,104^. A Drude model is used to describe the dielectric function of the gold nanorods, ${\epsilon }_{{\rm{Au}}}(\omega )=1-\frac{{\omega }_{{\rm{p}}}^{2}}{{\omega }^{2}+i\omega {\gamma }_{{\rm{p}}}},$ with *ℏω*_p_ = 8.2934 eV and *ℏγ*_p_ = 0.0928 eV. A dominant QNM is found when a potential *z* − polarized dipole is placed at the gap center, and we report its complex eigenfrequency and quality factor *Q*_*c*_ = *ω*_*c*_/*κ*_*c*_ in Table 1. The mode distribution ($| {\tilde{f}}_{z}{| }^{2}$) is shown in Fig. 2b. The QNM phase there is defined here by ${\tilde{f}}_{z}({\bf{r}})=| {\tilde{f}}_{z}({\bf{r}})| {e}^{i{\phi }_{0}({\bf{r}})}$ (dominant *z*-component), and we assume a dipole polarized in the *z*-direction such that the projected phase in Eq. (16) is equal to *ϕ*_0_. The distribution of $2{Q}_{{\rm{c}}}\tan (2{\phi }_{0})$ [which quantifies the deviation from the zero-phase spectral density as given by *ζ*_*c*_(*ϕ*_0_, *ω*)] is shown Fig. 2c. The value of the QNM at the dipole location as well as its phase is shown in Table 1. Figure 2e, f show the dipole decay rate under weak coupling [Eq. (15)] calculated with full Maxwell simulations (numerical Green function), as well as using the QNM expansion of Eq. (16). The agreement (overlap of the curves in the vicinity of the resonance) is quite good for a single mode and shows small improvements when a second mode with higher resonant frequency is included. We also plot in (f) and in all other similar figures in this section the line *ω*_0_/*ω*_*c*_, which is the QNM prediction with zero phase *ϕ*_0_ = 0, and in all cases *fails* to capture the correct trends^17^. The value ${\tilde{\Omega }}_{{\rm{BB}}}=0.1$ indicates that the broadband dissipative regime coincides with USC. The bandwidth of the system dynamics spans a number of bare cavity linewidths *κ*_*c*_ equal to $\sim {\tilde{\Omega }}_{{\rm{BB}}}{Q}_{c}\approx 2.6$, and comparing with Fig. 2f, the agreement with full Maxwell calculations remains accurate across this bandwidth, suggesting our ab initio approach can accurately be used in the single-mode dissipative USC regime to identify corrections beyond phenomenological models. Finally, we can use the spatially unspecified representation of our QNM quantization scheme to estimate the fraction of radiative vs. nonradiative dissipation for the bare (uncoupled) cavity mode; see the Supplementary Information and Ref. ^103^. We find a radiative proportion ${S}_{c}^{{\rm{rad}}}/{S}_{c}\approx 9.9$%, with ${S}_{c}={S}_{c}^{{\rm{rad}}}+{S}_{c}^{{\rm{nrad}}}\approx 1$.

In the Supplementary Information, we also consider a cylindrical gold dimer resonator, similar to the one studied in Ref. ^17^, which gives similar qualitative results, though with a much higher radiative photon loss fraction. Additionally, we discuss examples with smaller gaps (which can enable USC more easily through stronger field confinement) in “Experimental Prospects”.

#### Dielectric resonators

**Fig. 3:**
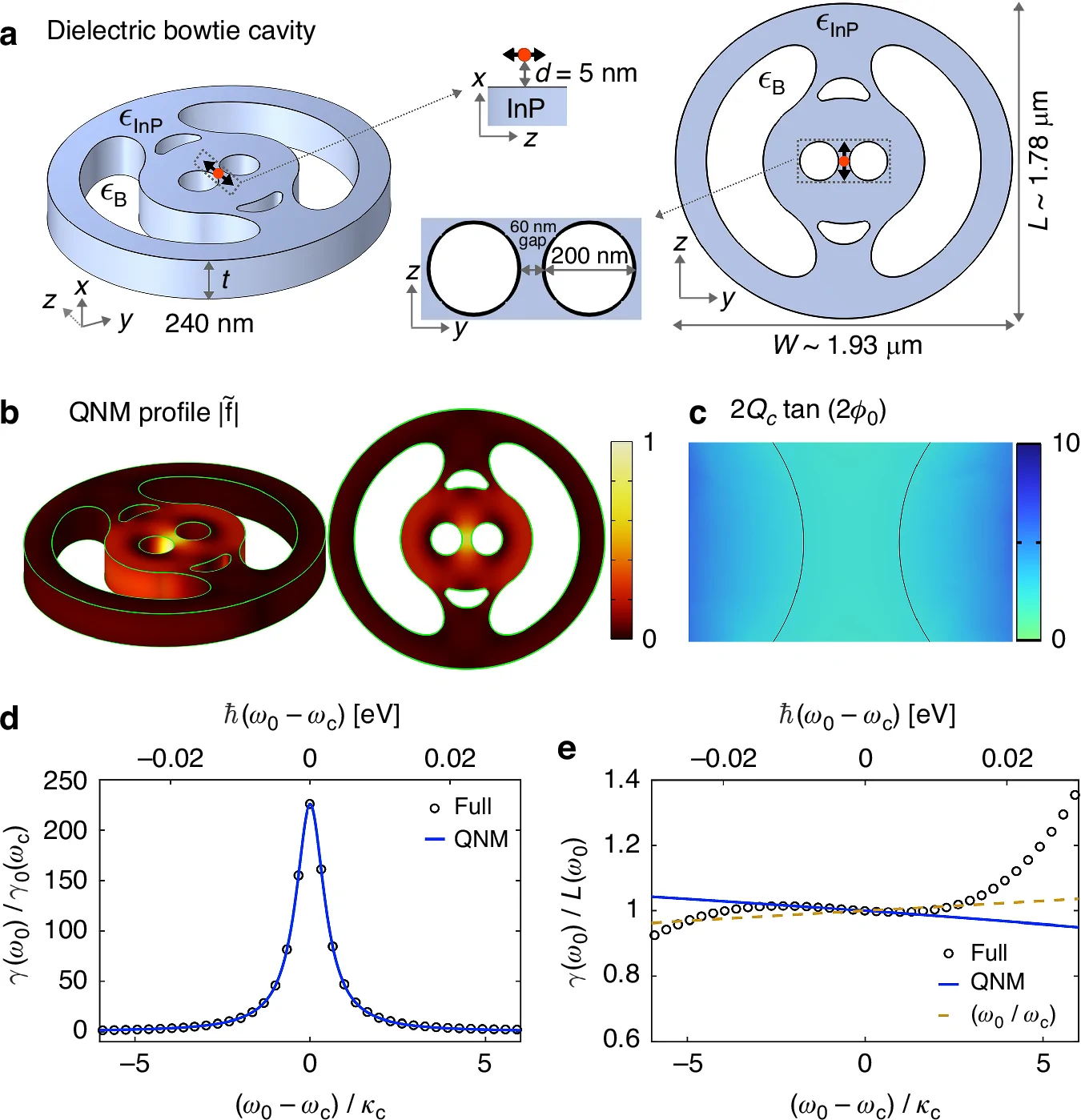
Results for dielectric bowtie cavity. **a** Schematic of 3D dielectric bowtie resonator in free space (*ϵ*_B_ = 1.0). **b** Mode profile of QNM $| \tilde{{\bf{f}}}|$ (including all components) (arb. units). **c** Phase distribution of $2{Q}_{{\rm{c}}}\tan (2{\phi }_{0})$ for *z* − component ${\tilde{f}}_{z}$, where the phase is defined from ${\tilde{f}}_{z}({\bf{r}})=| {\tilde{f}}_{z}({\bf{r}})| {e}^{i{\phi }_{0}({\bf{r}})}$. **d** Purcell factors for a *z* − polarized emitter 5 nm above the bridge gap center, normalized by the free-space rate *γ*_0_(*ω*) = ∣**d**∣^2^*ω*^3^/(3*πϵ*_0_*ℏc*^3^). **e** Decay rate normalized by Lorentzian function *L*_*c*_(*ω*_0_). Here, we plot the full dipole decay rate with an approximate background 1.77*γ*_0_(*ω*_0_) subtracted out. The dominant QNM parameters are found in Table 1

We now consider dielectric resonators. We first consider a 3D dielectric bowtie cavity design, as shown in Fig. 3, which references the design shown in Refs. ^105,106^. The potential *z* − polarized dipole is placed 5 nm above the bridge. The constant permittivity of the cavity material (Indium phosphide: InP) is *ϵ*_InP_ = 3.1649^2^ ≈ 10.02. Here, the agreement between full dipole simulations and the QNM expansion in the dipole decay rate predictions is excellent within a few linewidths of the resonance, but deviates beyond that, potentially due to the influence of additional modes. Interestingly, the value of ${\tilde{\Omega }}_{{\rm{BB}}}=0.028$ suggests that the broadband dissipative regime, where corrections to phenomenological dissipation models become significant, can occur *well below* the usual threshold of USC. Here, ${\tilde{\Omega }}_{{\rm{BB}}}{Q}_{c}\approx 4.5$, and some deviations at the edge of the band of dynamics with scaled dynamical rate on the threshold of the broadband dissipative regime (${\tilde{\Omega }}_{{\rm{BB}}}$) can be observed in Fig. 3e, suggesting some limits to the efficacy of single-mode models, though note we have made no effort to optimize the cavity design to mitigate this effect. For USC, however, the bandwidth of dynamics would become larger, and the prediction of the single-mode QNM theory would not be more accurate in this regime than phenomenological models.

**Fig. 4:**
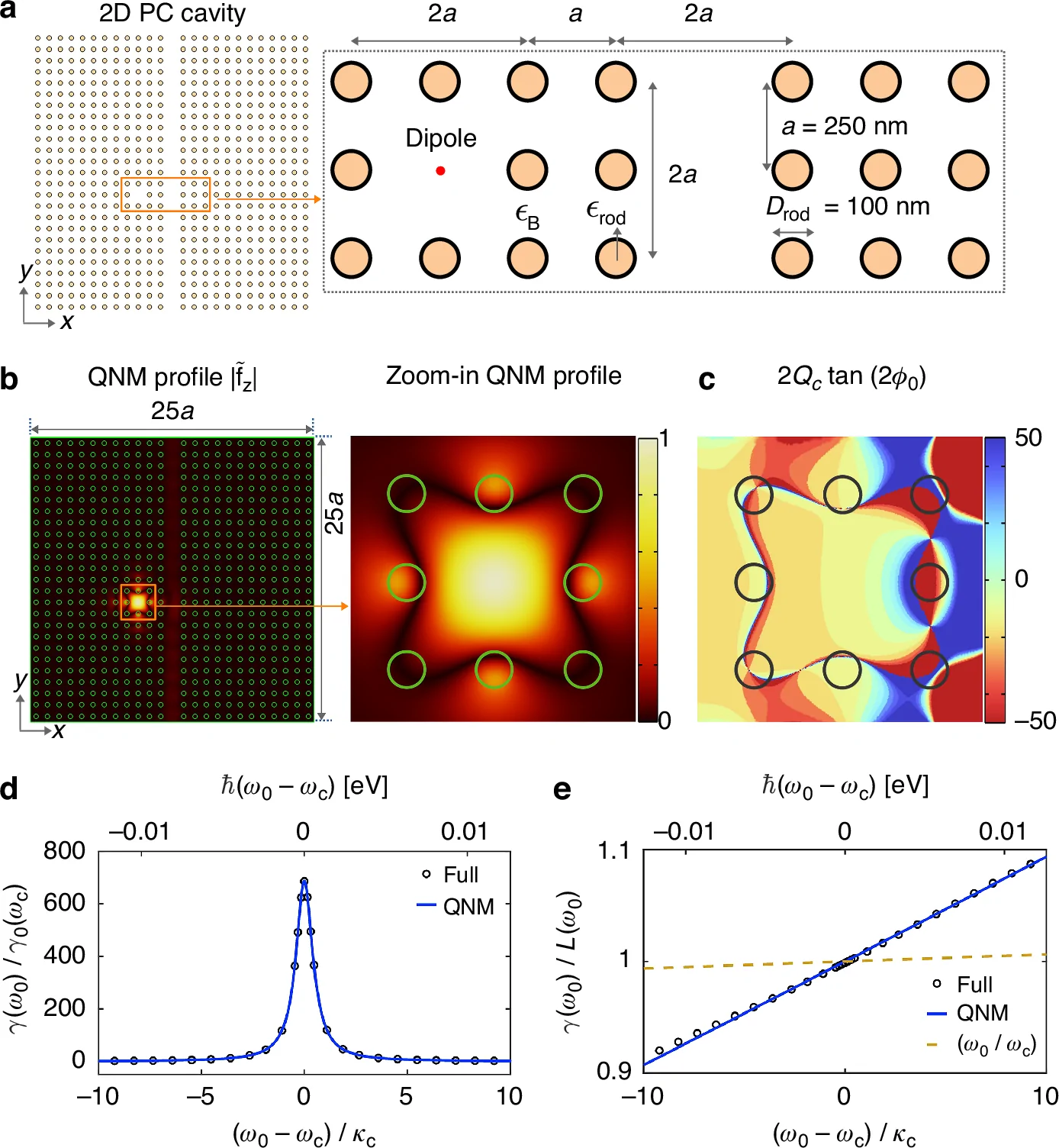
Results for 2D PC cavity. **a** Schematic of 2D PC cavity structure.**b** Mode profile for QNM $| {\tilde{{\bf{f}}}}_{z}|$ (arb. units; TM mode, the only component). **c** Phase distribution of $2{Q}_{{\rm{c}}}\tan (2{\phi }_{0})$ for dominant QNM, where the phase is defined from ${\tilde{f}}_{z}({\bf{r}})=| {\tilde{f}}_{z}({\bf{r}})| {e}^{i{\phi }_{0}({\bf{r}})}$. **d** Purcell factors for an emitter (line current) at the cavity center, normalized by the 2D free-space rate ${\gamma }_{0}^{{\rm{2D}}}(\omega )=| {{\bf{d}}}^{{\rm{2D}}}{| }^{2}{\omega }^{2}/(2{\epsilon }_{0}\hslash {c}^{2})$. **e** Decay rate normalized by Lorentzian function $L({\omega }_{0})={\gamma }_{c}^{{\rm{QNM}}}({\omega }_{c})\times \frac{{\kappa }_{c}^{2}/4}{{\kappa }_{c}^{2}/4+{({\omega }_{0}-{\omega }_{c})}^{2}}$. Here, we plot the full dipole decay rate with an approximate background $0.05{\gamma }_{0}^{{\rm{2D}}}({\omega }_{0})$ subtracted out. The dominant QNM parameters are found in Table 1

Next, we consider a 2D photonic crystal (PC) design with very low material loss, consisting of a point defect (cavity) coupled to a line defect (waveguide), which we show in Fig. 4. The potential dipole (line current) is placed at the cavity center. The dielectric function *ϵ*_rod_(*ω*) of the nanorods is described by a single Lorentz oscillator model, ${\epsilon }_{{\rm{rod}}}(\omega )={\epsilon }_{\infty }-\frac{({\epsilon }_{{\rm{s}}}-{\epsilon }_{\infty }){\omega }_{{\rm{t}}}^{2}}{{\omega }^{2}-{\omega }_{{\rm{t}}}^{2}+i\omega {\gamma }_{{\rm{L}}}},$ with ${\epsilon }_{\infty }={n}_{{\rm{B}}}^{2}=1.0$, *ϵ*_s_ = 8.9, *ℏω*_t_ = 24.12 eV, and *ℏγ*_L_ = 0.131 eV. Here, the projected QNM phase plot in Fig. 4c shows rich and asymmetric features, highlighting the possibility of engineering broadband dissipative behavior by spatial positioning of the dipole. The full calculation of the dipole decay rate again shows good agreement with a single QNM expansion, and the value ${\tilde{\Omega }}_{{\rm{BB}}}=0.0036$ reveals that the broadband regime can be reached far below the threshold for USC. However, here, ∣*η*^(1)^∣ takes the value 0.074, which, being less than the threshold for USC, indicates that single-mode models of USC will fail to capture accurate corrections to dynamics beyond phenomenological models of dissipation. Here we obtain a radiative decay fraction of ${S}_{c}^{{\rm{rad}}}/{S}_{c}\approx 79$%, where we calculate ${S}_{c}^{{\rm{nrad}}}$ and approximate ${S}_{c}^{{\rm{rad}}}={S}_{c}-{S}_{c}^{{\rm{nrad}}}$ and *S*_*c*_ ≈ 1.

In the Supplementary Information we show an additional example for a 3D PC cavity mode, similar to the one studied in Ref. ^17^. We also consider a 2D lossy microdisk with a dominant whispering-gallery mode, for which we find single-mode corrections in the broadband dissipative regime to be inaccurate, which we attribute to the quasi-harmonic mode distribution associated with quasi-1D resonators leading to an intrinsically multimoded broadband dissipative regime.

In summary, these results suggest that low-Q plasmonic resonators generally have a broadband dissipation regime that coincides with the USC regime (in terms of resonant light-matter coupling strength), and dynamical corrections to the predictions of phenomenological theories of their photon dissipation can be captured by our corrected and ab initio approach well into the USC regime. In contrast, the dielectric systems we study have larger quality factors and will likely require multimode models for accurate description in the USC regime (even when other modes are very spectrally isolated from the dominant one). However, these systems also enter the broadband dissipative regime with (resonant) coupling strengths far below the threshold of USC, where our single-mode ab initio approach can accurately predict broadband dissipative corrections for certain cavity designs. Based on these results, we pick the plasmonic dimer systems as our main example for our study of USC using the ab initio single-mode ME, which we shall use going forward as rough guides for our parameter sets to study quantum dynamics in the next section.

### Quantum ME simulations in the USC regime

In this section we numerically solve the Coulomb gauge ME in the single-mode limit, using the QNM frequency-dependent dissipation rates from Eq. (10). The system level Hamiltonian is given by Eq. (7). Explicitly, the ME is

17 $$\begin{array}{ll}\dot{\rho }\,=\,-\frac{i}{\hslash }[{\hat{{\mathcal{H}}}}_{{\rm{C}}}^{{\rm{S}}},\rho ]+2\pi {\sum }_{\alpha ,{\alpha }^{{\prime} }}\left({c}_{\alpha }^{\Pi }{c}_{{\alpha }^{{\prime} }}^{\Pi * }{\Lambda }^{2}({\omega }_{\alpha })\left[{\hat{\sigma }}_{\alpha }\rho {\hat{\sigma }}_{{\alpha }^{{\prime} }}^{\dagger }-{\hat{\sigma }}_{\alpha {\prime} }^{\dagger }{\hat{\sigma }}_{\alpha }\rho \right]+\,\mathrm{H.c.}\,\right)\end{array}$$

and here we have not made the secular approximation from “Ab initio ME for broadband dissipative regime” for greater accuracy of simulations. We also made two generalizations to be able to compare with more phenomenological theories of cavity-reservoir coupling: (i) we have written the spectral density in general terms as Λ^2^(*ω*_*α*_), where the correct ab initio result is given by ${\Lambda }_{c}(\omega )=\sqrt{\frac{{\kappa }_{c}}{2\pi }\frac{{\omega }_{c}}{\omega }{\zeta }_{c}({\phi }_{0},\omega )}$^17^, which can be deduced from Eq. (10), and (ii) we have expressed the matrix element ${c}_{\alpha }^{\Pi }=\left\langle j\right\vert \hat{\Pi }\left\vert k\right\rangle$ in terms of a general system operator $\hat{\Pi }$ which couples to the reservoir. This allows us to compare with a common phenomenological model of cavity-reservoir coupling which can be expressed in terms of an effective system-reservoir Hamiltonian of the form ${\hat{H}}_{{\rm{phen}}}=\hslash \int\,d\omega \Lambda (\omega )\left[\hat{\Pi }{\hat{d}}_{\omega }+\,\mathrm{H.c.}\,\right],$ where $[{\hat{d}}_{\omega },{\hat{d}}_{\omega {\prime} }^{\dagger }]=\delta (\omega -\omega {\prime} )$.

To model excitation, we consider an incoherent drive which can be modeled by adding to the ME in Eq. (9) the term

18 $$\dot{\rho}\,\to \dot{\rho}\,+\,2\pi \sum _{\alpha ,{\alpha}^{{\prime}}}({c}_{\alpha}{c}_{{\alpha }^{{\prime}}}^{\ast }{{\Lambda}}_{\mathrm{inc}}^{2}({\omega}_{\alpha})[{\hat{\sigma}}_{\alpha}^{\dagger}\rho {\hat{\sigma}}_{{\alpha}^{{\prime}}}-{\hat{\sigma}}_{\alpha {{\prime}}}{\hat{\sigma}}_{\alpha}^{\dagger}\rho ]+\,{\mathrm{H}}.{\mathrm{c}}.\,)$$

where we have chosen to model cavity driving with matrix elements *c*_*α*_ calculated from ${\hat{a}}_{c}$, and we choose the incoherent excitation spectral density to take the form ${\Lambda }_{{\rm{inc}}}^{2}({\omega }_{\alpha })={\gamma }_{{\rm{inc}}}{\omega }_{c}/(2{\rm{\pi}} {\omega }_{\alpha })$. Other forms of incoherent (or coherent^33^) excitation are possible, and will generally depend on the physical model of excitation; we choose this simple form as it recovers results from a simple classical heuristic model of incoherent excitation when we compare with the bosonic Hopfield model (which we will show in future work), which has been previously shown to have a quantum-classical correspondence in a phenomenological dissipation model^40^. Additionally, other noise sources may also be present in realistic systems, especially at room temperature, as arising from (e.g.) pure dephasing^107^, phonon scattering in solid-state systems^108^, or intramolecular vibrations. For the sake of analyzing the underlying *fundamental* quantum optical characteristics of photon loss, we neglect these here.

From the ME solution, we can calculate the steady-state intracavity (specifically, near-field) spectrum, which should be calculated in terms of matrix elements of the transverse electric field operator^67^ (see Supplementary Information for further discussion). This is accomplished by defining ${\hat{x}}_{\det }={\sum }_{\alpha }{c}_{\alpha }^{\det }{\hat{\sigma }}_{\alpha }$, where

19 $${c}_{\alpha }^{\det }=\left\langle j\right\vert i{\eta }_{c}{\hat{a}}_{c}-i{\eta }_{c}^{* }{\hat{a}}_{c}^{\dagger }\left\vert k\right\rangle$$

and the intracavity spectrum is

20 $${S}_{c}(\omega )={\mathop{\lim }\limits_{t\to \infty }}\,\mathrm{Re}\,\left\{\int_{0}^{\infty }d\tau {e}^{i\omega \tau }\langle {\hat{x}}_{\det }^{\dagger }(t){\hat{x}}_{\det }(t+\tau )\rangle \right\}$$

Here we have assumed the projected QNM phase at the detector location to be approximately the same as at the emitter location, such that the matrix elements can be calculated from Eq. (19). Other observables, such as the second order degree of coherence *g*^(2)^(*τ*) could also be calculated if desired^33^.

**Fig. 5:**
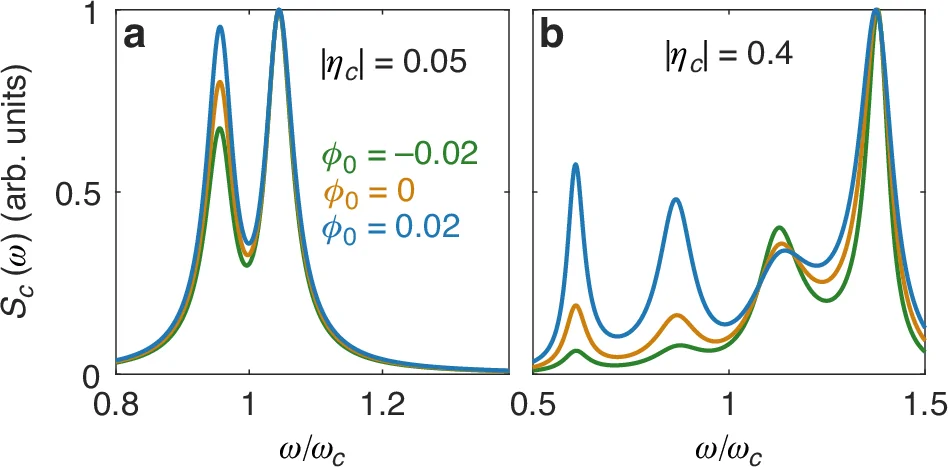
Cavity spectrum for varying QNM phases. Normalized intracavity spectrum for a single cavity mode with *Q*_*c*_ = 13 for a TLS coupled with **a** ∣*η*_*c*_∣ = 0.05 and **b** ∣*η*_*c*_∣ = 0.4. Spectra are shown for values of the projected QNM phase at the dipole location of *ϕ*_0_ = − 0.02 (green), *ϕ*_0_ = 0 (orange), and *ϕ*_0_ = 0.02 (blue; most similar to the dimer value of ~0.014 in Ref. ^17^). Here we use *γ*_inc_ = *κ*_*c*_/100, which can probe beyond the weak-excitation regime

**Fig. 6:**
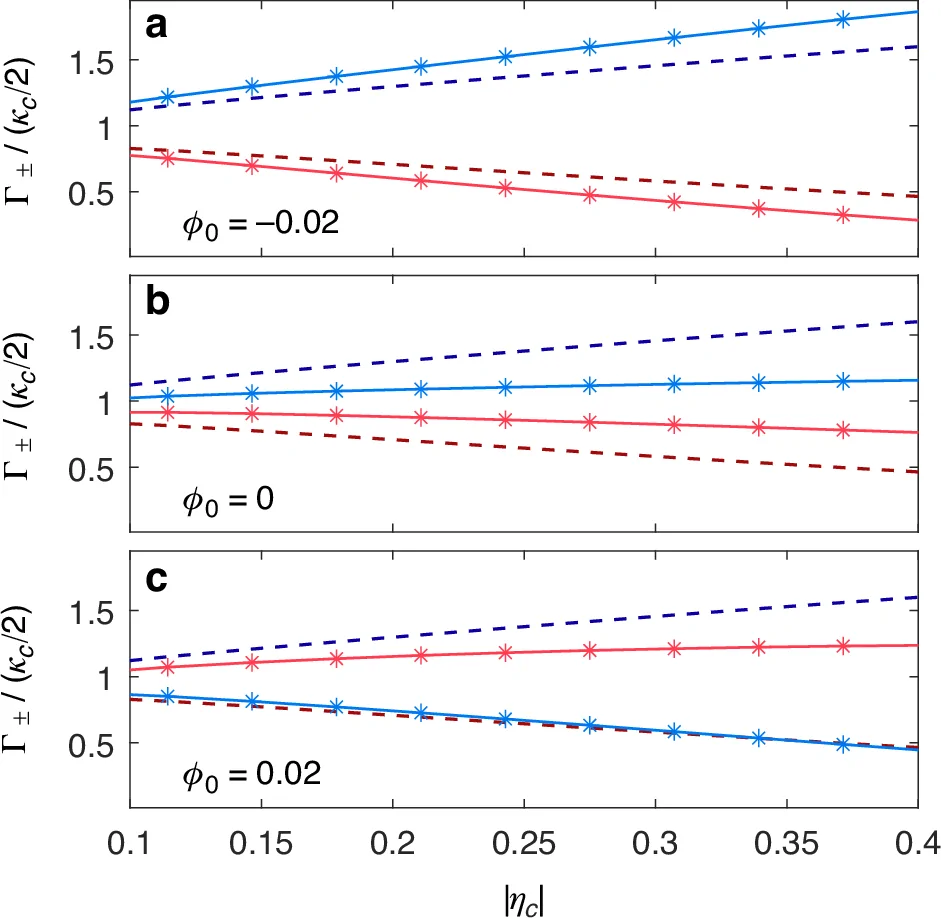
Cavity spectrum linewidths. Linewidths of dominant two peaks in the intracavity spectrum (red-shifted peak in red, blue-shifted peak in blue) for a single cavity mode with *Q*_*c*_ = 20 for a TLS coupled to the mode with a projected QNM phase at the TLS location of **a** *ϕ*_0_ = − 0.02, **b** *ϕ*_0_ = 0, and **c** *ϕ*_0_ = 0.02. Here we let the incoherent drive be sufficiently weak such that the trends converge (*γ*_inc_ = *κ*_*c*_/10^4^ is sufficient). The dark red and blue dashed lines give the result for a naive flat spectral density ${\Lambda }_{{\rm{flat}}}(\omega )=\sqrt{{\kappa }_{c}/(2\pi )}$

We first simulate intracavity spectra using our ab initio quantum ME model in the spatially specified representation using a value of *Q*_*c*_ = 13. In Fig. 5 we show the highly significant effect (even below the traditional USC threshold ∣*η*_*c*_∣ > 0.1) of varying the QNM phase at the dipole location on the emission spectra. Here we use the correct single-mode spectral density Λ_*c*_(*ω*). We stress again that the broadband dissipative regime criteria $| {\eta }_{c}| \ge {\tilde{\Omega }}_{{\rm{BB}}}$ (for the *ω*_0_ = *ω*_*c*_ case studied here) is only a rough metric; indeed, here for ∣*η*_*c*_∣ = 0.05 significant deviations from symmetric spectra (i.e., broadband dissipative effects) can be observed even for the *ϕ*_0_ ≥ 0 case, where ${\tilde{\Omega }}_{{\rm{BB}}}\approx 0.1$,

Next, we investigate the effect of using the correct spectral density and QNM phase on the linewidths (full width at half-maximum) of the emission spectrum in the weak-excitation limit. In Fig. 6 we fix *Q*_*c*_ = 20 and vary the coupling strength ∣*η*_*c*_∣ for a variety of different projected QNM phases at the dipole location, and fit the resulting peaks to a two-Lorentzian function, plotting the linewidths of the resulting dominant two spectral peaks. Note that the Quantum Rabi model and its Coulomb gauge equivalent, ${\hat{{\mathcal{H}}}}_{{\rm{C}}}^{{\rm{S}}}$, do not have a classical correspondence in the weak-excitation regime due to virtual excitations in the ground state giving rise to fundamental anharmonicity, which manifests as a multi-peak structure in the emission spectra for sufficiently large ∣*η*_*c*_∣ ^40^; here we show the linewidths of the most dominant peaks which correspond to the Jaynes-Cummings peaks for sufficiently small cavity-TLS coupling.

These trends agree well with the perturbative analytical solution given by Eq. (13). Interestingly, this equation predicts the existence of a certain ${\phi }_{0}^{* }\approx 1/(8{Q}_{c})$ such that the linewidths remain symmetrical to leading order in ∣*η*_*c*_∣, even in the USC regime, which we have verified numerically (not shown). The spectrum itself is not necessarily symmetric as well in this case, as this depends on the specific excitation model. The persistent asymmetries in spectral linewidths (observable even below the USC regime) have also been predicted using purely classical ab initio models^109^.

**Fig. 7:**
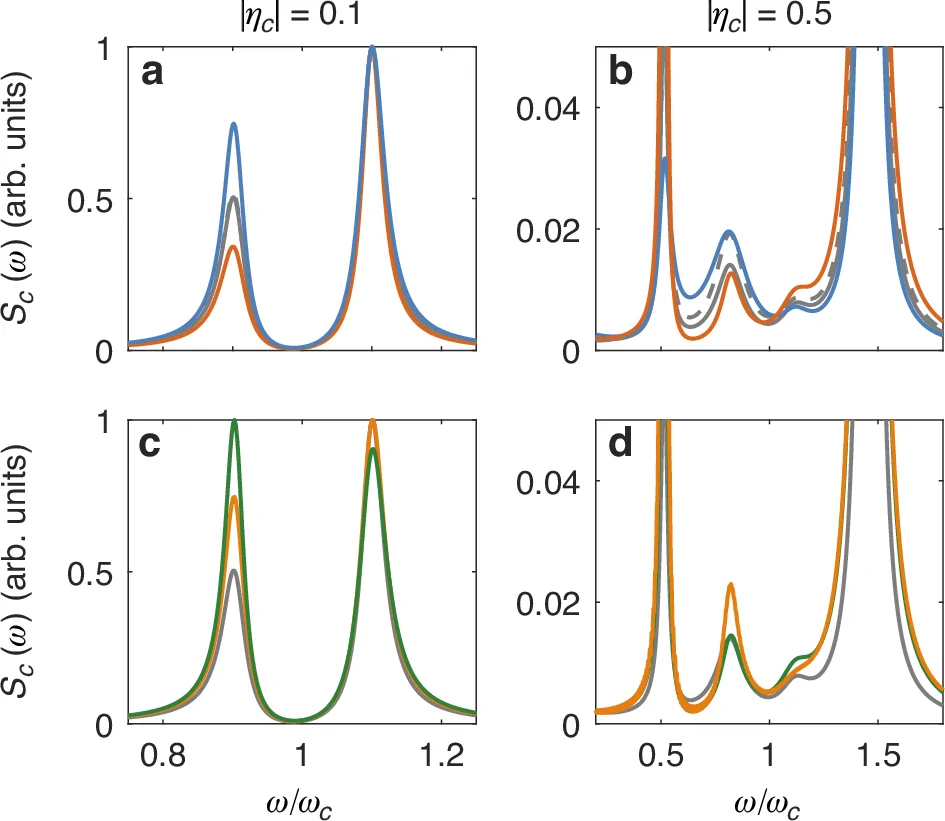
Cavity spectrum for varying system-reservoir coupling forms. Normalized cavity spectrum for gold dimer-like cavity with *Q*_*c*_ = 13, *ϕ*_0_ = 0.0137, *γ*_inc_ = *κ*_*c*_/100, and **a**, **b** variable system-reservoir coupling operator $\Pi ={\hat{a}}_{c}$ (grey, solid), $\Pi =(\hat{Q}+\hat{P})/\sqrt{2}$ (grey, dashed), $\hat{\Pi }=\hat{Q}$ (red, solid), and $\hat{\Pi }=\hat{P}$ (blue, solid). **c**, **d** cavity spectra with fixed (correct) $\hat{\Pi }={\hat{a}}_{c}$ and ${\Lambda }_{n}^{2}(\omega )=\frac{\kappa }{2\pi }{\left(\omega /{\omega }_{c}\right)}^{n}{\zeta }_{c}({\phi }_{0},\omega )$ with *n* = − 1 (grey) *n* = 0 (orange), and *n* = 1 (green)

Next, we study the effect of the cavity-reservoir coupling operator $\hat{\Pi }$ (with $\hat{\Pi }={\hat{a}}_{c}$ being the correct choice in our ab initio quantized QNM model). Often in the literature a phenomenological choice of $\hat{Q}={\hat{a}}_{c}+{\hat{a}}_{c}^{\dagger }$ or $\hat{P}=i({\hat{a}}_{c}^{\dagger }-{\hat{a}}_{c})$ is assumed^110^. In Fig. 7, we show the cavity spectrum for different choices of $\hat{\Pi }$, showing the significant effect of the choice of reservoir-coupling operator. Interestingly, the choice of $\hat{\Pi }=(\hat{Q}+\hat{P})/\sqrt{2}$ (and $(\hat{Q}-\hat{P})/\sqrt{2}$ not shown, but gives equivalent results) gives very similar results to the ab initio $\hat{\Pi }={\hat{a}}_{c}$. Recently, both of these forms were shown to give a classical-quantum correspondence in models of ultrastrong coupling to bosonic matter with a phenomenological dissipative normal mode approach^40^. Also in Fig.  7, we show the effect of using different spectral densities on the cavity spectra.

Overall, our results highlight important dissipative effects that are not captured by the typical phenomenological light-matter theories. Such effects are not restricted to USC, but more generally apply in broadband regimes where the ratio of a characteristic dynamical rate of the system to the cavity frequency exceeds ${\tilde{\Omega }}_{{\rm{BB}}}$; i.e., when it approaches ${\left\vert 1-4{Q}_{c}\tan (2{\phi }_{0})\right\vert }^{-1}$. As we have shown with our dielectric cavity examples, this can occur in realistic systems with (e.g.) TLS-cavity coupling constants orders of magnitude lower than those required to enter the USC regime.

## Discussion

### Experimental prospects

One of the key advantages of our ab initio quantized QNM approach to the broadband dissipative regime of cavity-QED is its applicability to emerging optical experiments in USC and near-USC. Advances in both plasmonic and subdiffraction limit dielectric resonator design and fabrication^111–114^ are rapidly approaching the USC and near-USC regimes, and indeed, our finding that the broadband dissipative regime can be reached for dielectric systems with coupling strengths orders of magnitude below the usual threshold for USC indicates that the corrections we report in this work may be observable in a variety of currently accessible platforms. For example, experimental detection of asymmetries in the spectrum or polariton branch decay rates could serve as signatures of broadband dissipative effects. Though spectral effects may manifest differently in the far-field (for which a full input-output theory remains to be developed), near-field detection can be implemented with an auxilliary weakly-coupled TLS “detector”, and far-field time-resolved measurements could potentially resolve differential decay rates. The sharp dependence of broadband dissipative effects on QNM phase also suggests that position-dependent measurements could prove useful to this end. Some of these features may be difficult to observe due to spectral filtering requirements, or competing decoherence channels (including pure dephasing^107^, phonon coupling in quantum dots^108^, or intramolecular vibrations), particularly at high temperature. Nonetheless, at least at low temperatures with quantum dots, phonon effects are well-understood, and their competing influence can easily be analyzed by adapting our master equation formalism^108,115,116^, and moreover, plasmonic cavities have such high decay rates that the competing influence of such channels may be relatively negligible^117^.

In the remainder of this section, we give a brief estimate of achievable normalized single-mode coupling strengths ∣*η*_*c*_∣ for reasonable dipole moments for the cavity designs we study in this work, followed by a comparison with some experimentally demonstrated subdiffraction dielectric and plasmonic cavities.

Using the definition of *η*_*c*_ from Eq. (8), and letting ${\tilde{{\bf{f}}}}_{c}^{{\rm{s}}}\approx {\tilde{{\bf{f}}}}_{c}$, we can express the normalized single-mode coupling strength for a given resonator structure as

21 $$| {\eta }_{c}| \approx 9.5\times 1{0}^{-14}{\mathrm{eV}}^{-\frac{1}{2}}{\mathrm{m}}^{\frac{3}{2}}\left[\frac{d}{{d}_{0}}\right]\left[\frac{| {\tilde{{\bf{f}}}}_{c}| }{\sqrt{\hslash {\omega }_{c}}}\right]$$

where we assume the field amplitude to be dominated by a single direction, which the dipole moment is oriented parallel to, and *d*_0_ = 1 e ⋅ nm is a reference dipole moment. With *d* = *d*_0_, the predicted ∣*η*_*c*_∣ value is given in Table 2. For the 2D PC cavity and the microdisk modes, we estimate an effective 3D QNM amplitude by scaling the 2D QNM mode profiles by ${l}_{{\rm{eff}}}^{-1/2}$; here we use a value of *l*_eff_ = 100 nm, which is half of the actual thickness of the 3D PC example. Although the predicted ∣*η*_*c*_∣ for dipole moment *d*_0_ is below the broadband dissipative threshold ${\tilde{\Omega }}_{{\rm{BB}}}$ for all examples, it is important to note that we have made no particular effort to optimize for ultrasmall mode volume in our cavity designs. Inverse design techniques in nanophotonics could likely easily increase all of these coupling rates significantly, which is another advantage of our arbitrary cavity mode approach. This has been shown, for example, with waveguide mode Purcell factors, including the design of chiral emitter coupling rates^118^. Similar methods can be used to optimize QNM properties, which will be a topic of future work.

**Table 2:** ∣*η*_*c*_∣ for example cavity structures studied in “Results and Simulations for Various Cavity Designs Under Weak-Coupling” and Supplementary Information) with dipole moment *d* = 1 e ⋅ nm

	∣*η*_*c*_∣
Ellipsoid Dimer	0.02
Cyl. Dimer	0.006
Bowtie	0.0002
2D PC	0.0007
3D PC Beam	0.0001
Microdisk	0.00003

Note for the first example, this coupling rate can easily be increased by decreasing the gap, which is discussed in more detail in the main text

Using a recently demonstrated topology-optimized dielectric cavity^105,112^, which far surpasses the (bulk-like cavity) diffraction limit, and *d* = *d*_0_, we find ∣*η*_*c*_∣ = 0.003. Assuming this structure, similar to our bowtie cavity design, has a similar QNM phase above the bridge center ($\tan (2{\phi }_{0})\approx 0.007$), using the quality factor *Q*_*c*_ ≈ 1100 from Ref. ^112^, we predict that broadband dissipative effects will become observable for ${\tilde{\Omega }}_{{\rm{BB}}}\approx 0.003$. The coinciding of this broadband dissipative region threshold parameter with our estimated ∣*η*_*c*_∣ for a dipole moment *d*_0_ (of the same order of magnitude as common dipole couplings including organic dye molecules^117^ and InAs/GaAs quantum dots; higher dipole moments can be achieved in, e.g., CdSe/CdS quantum dots^29^) indicates that observation of these effects may be possible in cavity designs fabricated using existing modern techniques.

Similarly, the PC beam cavity from Ref. ^119^ gives ∣*η*_*c*_∣ ≈ 0.007 for *d* = *d*_0_, assuming the dipole is placed in air with index of refraction *n* = 1. Using the same QNM phase as our PC beam cavity, and assuming a quality-factor *Q* ≈ 10^6^, this gives ${\tilde{\Omega }}_{{\rm{BB}}}=2\,{\times}1{0}^{-5}$.

In plasmonic systems, near-USC has already been demonstrated using single quantum dots coupled to plasmonic nanoparticles. For example, a USC threshold coupling of ∣*η*_*c*_∣ = 0.1 was recently observed at room temperature^29^, and similarly near-USC ∣*η*_*c*_∣ ≈ 0.06 was also achieved recently with gold nanorods^120^. Beyond quantum dots, organic dye molecules (with dipole moments on the order of ~*d*_0_) were previously observed with ∣*η*_*c*_∣ ≈ 0.04 using a few-molecule system^117^. Theoretical calculations using time-dependent density functional theory have also predicted the achievability of single-dipole USC coupling with dye molecules^121^. Beyond single (or few) dipole coupling, collective USC couplings between nanoparticle plasmons and cavity modes have already been reached^28,122^, and also near-USC dipole-photon coupling^123^.

Finally, we remark that by decreasing the gap size in our presented dimer simulations, significant decreases in mode volume (and thus enhanced light-matter coupling strength) can be predicted. For example, using an ellipsoidal gold dimer cavity with dimensions *W*_elli_ = 8 nm and *L*_elli_ = 40 nm (similar to the one shown in Fig. 3) but decreasing the gap size to *d*_elli_ = 0.93 nm, we find a quality factor *Q*_*c*_ ≈ 18.5 and corresponding ∣*η*_*c*_∣ ≈ 0.18 for dipole moment *d*_0_/2 = 0.5 e ⋅ nm. It should be noted, however, that with such small gaps the contribution of quasistatic couplings beyond our single transverse mode model presented in this work likely becomes significant^124^. Possible breakdown of the dipole approximation in such regimes can also be treated within our formalism, as shown in Ref. ^67^, with slight model modifications.

### Conclusions

In this paper, we have presented an ab initio approach to single-mode MEs for coupled cavity-dipole systems that remain valid in the USC regime, which is, to the best of our knowledge, the first time this has been achieved for realistic 3D cavity geometries. We introduced and defined the broadband dissipative regime of cavity-QED, where corrections to quantum observables begin to deviate substantially from predictions made with standard ME models, of which the USC regime is contained as a subset, but in fact can occur for system dynamical parameters (e.g., light-matter coupling strengths) orders of magnitude below the usual threshold for USC. We assessed the validity of our quantized QNM approach for a variety of plasmonic and dielectric cavity designs, and performed quantum simulations in the USC regime to compare significant differences between the predictions of the ab initio quantized QNM theory, when compared with standard phenomenological models. We also posited a set of potential heuristic rules regarding the ability and limitations of single-mode models to accurately model the broadband dissipative regime for dielectric and plasmonic structures in “Results and Simulations for Various Cavity Designs Under Weak-Coupling”. We finally showed how the effects we identify associated with broadband dissipative and USC regimes should be observable in a variety of optical platforms in the near future.

There are numerous ways our approach could be generalized and built upon, and we expect this work to spur further research in these directions. For one, we have restricted ourselves to a study of near-field observables, leaving the more subtle problem of connecting cavity dynamics to input-output relations of scattered quantum fields to future work. Additionally, we have neglected the influence of background dipole couplings of the TLS to non-cavity reservoir modes, which with some generalization could be self-consistently included in our formalism. An extension to far-field observables (input-output theory) would require both these background terms, as well as consideration of the multiple cavity-TLS resonances across a broad bandwidth, likely requiring a generalized Markov condition. Finally, our presentation has been limited to a single QNM and a single dipole (or many dipoles at locations with identical QNM phases) for now; while the system-reservoir Hamiltonian we derived can be valid for an arbitrary number of QNMs and dipoles, the generalization of how to transform to the spatially specified representation in a general system with multiple modes and/or multiple dipoles placed at locations with differing QNM phases will require further work. We anticipate that a more systematic treatment of the different representations of quantized QNMs (spatially specified vs unspecified) will shed light on this important question. Fully generalizing our approach to a multi-QNM system would be especially impactful, as our results indicate that many dielectric cavities can not be accurately modeled with just a single-mode in the broadband dissipative regime of cavity-QED. Finally, a comparison of our results with an HEOM or generalized pseudomode study using an ab initio description of the system QNMs would be an interesting evaluation of both methods.

## Materials and Methods

The classical QNM solutions are obtained from an inverse Greens function expansion approach using a dipole source in complex frequency space, with the use of COMSOL Multiphysics^125^. Full dipole numerical solutions (no mode approximations) are also performed within COMSOL Multiphysics. Further details can be found in Refs. ^54,103,126^ for similar single-mode resonators, including metal dimers, 3D PC beam cavities, and 2D microdisks. The quantum simulations were performed by numerical integration of the master equation.

## Supplementary information

Supplementary Information

## Data Availability

Data underlying the results presented in this paper are not publicly available at this time but may be obtained from the authors upon reasonable request.
