# Supplementary material for: Dissipation in the broadband and ultrastrong coupling regimes of cavity quantum electrodynamics: an ab initio quantized quasinormal mode approach
Source: Light Sci Appl. 2026 Aug 26;15:364. doi: 10.1038/s41377-026-02406-2 (PMC13518348; doi:10.1038/s41377-026-02406-2)
Supplement: Supplementary file 1 [file 41377_2026_2406_MOESM1_ESM.pdf]

Supplementary Information for "Dissipation in the Broadband and Ultrastrong Coupling Regimes of Cavity Quantum Electrodynamics: An *Ab Initio* Quantized Quasinormal Mode Approach"

Chris Gustin,[1,4,*] Juanjuan Ren,[2] Sebastian Franke,[2,3] and Stephen Hughes[2]

[1] *E. L. Ginzton Laboratory, Stanford University, Stanford, California 94305, USA*
[2] *Department of Physics, Engineering Physics, and Astronomy, Queen's University, Kingston, Ontario K7L 3N6, Canada*
[3] *Technische Universität Berlin, Institut für Theoretische Physik, Nichtlineare Optik und Quantenelektronik, Hardenbergstraße 36, 10623 Berlin, Germany*
[4] *Present Address: Center for Hybrid Quantum Networks (Hy-Q), Niels Bohr Institute, University of Copenhagen, Jagtvej 155A, Copenhagen DK-2200, Denmark*

In this supplementary information, we include (i) a detailed derivation of the quantized QNM ME used in the main text to model the broadband dissipative regime of cavity-QED, (ii) additional resonator examples including a cylindrical gold dimer, 3D PC beam cavity, and a 2D microdisk, (iii) a derivation of the vanishing of the background component of the transverse vector potential, (iv) a derivation of the system-reservoir Hamiltonian with the quantized QNMs defined with respect to an electric field expansion, (v) a justification of the approximation that the residual reservoir operators behave effectively locally under the Markov approximation, and (vi) a derivation of the approximate expression for the TLS-cavity system linewidths used in the main text.

CONTENTS

I. Details of theoretical formalism 2
A. Quantization in spatially-dependent absorptive and dispersing media 2
B. Classical field quasinormal mode theory 3
C. Quantized quasinormal modes and reservoir modes 5
1. Projection of discrete oscillator subspace from continuum polariton excitations of EM fields and the medium 5
2. Construction of quantized quasinormal modes 6
3. Spatially specified form in the single-mode approximation 8
D. Generalized minimal coupling replacement 9
E. System-reservoir Hamiltonian 11
F. Quasinormal mode master equation in the ultrastrong coupling regime 12
1. Dressed-state basis 12
2. Second-order Born-Markov master equation 13
3. Single-mode limit and spatially specified representation 14
G. Output observables and near-field detection 15

II. Additional resonator examples 16

III. Vanishing of background transverse vector potential operator 18

IV. Derivation of Hamiltonian with quantized quasinormal mode operators defined with respect to an electric field expansion 19

V. Justification of the local bosonic commutator assumption 21

VI. Perturbative analytic expression for linewidths under weak-excitation 21

References 23

* chris.gustin@nbi.ku.dk

I. DETAILS OF THEORETICAL FORMALISM

In this section, we give a detailed derivation of our *ab initio* quantized QNM ME, and outline the underlying formalisms.

Before doing so, for convenience, we briefly give a summary of the relevant approximations that enter into our final model: We assume a single quantized mode, single-dipole theory, where the emitter dipole is treated as a TLS. The transverse field is assumed to be described near the resonator entirely by a QNM Green function expansion, neglecting the role of any background (e.g., a homogeneous medium component) contributions. The role of the longitudinal field is (in the final master equation) assumed to only affect the energy levels of the bare TLS. A Markov approximation is assumed for the coupling of the cavity mode to its environment, which is valid for all but extremely small Q factors where the mode becomes poorly defined. We also assume the dipole approximation for the sake of simplicity. Finally, we exclude consideration of the so-called "deep-strong coupling regime", where the cavity-TLS coupling is the same magnitude as (or larger than) the bare system frequencies, as numerous technical issues arise in this regime relating to the treatment of unphysical negative frequencies (and this regime is also very difficult to reach with optical frequencies).

A. Quantization in spatially-dependent absorptive and dispersing media

First we briefly describe the theory of macroscopic QED, which is a quantization scheme for arbitrary spatial-inhomogeneous and absorptive media [1–4]. We concentrate on the EM part of the Hamiltonian in passive materials and do not introduce any active emitter contributions yet, as this is later added via gauge-invariant coupling to a TLS.

The Hamiltonian of the combined medium (e.g., cavity resonator + environment) and photon field can be written in the general form [1, 5],

$$\hat{H}_{\rm em} = \int_0^\infty d\omega \int d^3r \ \hbar\omega \hat{\mathbf{b}}^\dagger(\mathbf{r},\omega)\cdot\hat{\mathbf{b}}(\mathbf{r},\omega), \tag{1}$$

where the vector-valued and spatially-dependent bosonic operators $\hat{\mathbf{b}}(\mathbf{r},\omega)$ represent the fundamental variables of the polariton (medium-photon) degrees of freedom with mode indices ω and $\mathbf{r}$, where $[\hat{b}_i(\mathbf{r},\omega), \hat{b}_j^\dagger(\mathbf{r}',\omega'] = \delta_{ij}\delta(\mathbf{r}-\mathbf{r}')\delta(\omega-\omega')$. The associated medium-assisted electric field operator $\hat{\mathbf{E}}_{\rm F}(\mathbf{r},\omega)$ fulfills the (quantum) Helmholtz equation:

$$\left[\boldsymbol{\nabla}\times\boldsymbol{\nabla}\times -\frac{\omega^2}{c^2}\epsilon(\mathbf{r},\omega)\right]\hat{\mathbf{E}}_{\rm F}(\mathbf{r},\omega) = i\omega\mu_0\hat{\mathbf{j}}_{\rm N}(\mathbf{r},\omega), \tag{2}$$

where $\epsilon(\mathbf{r},\omega) = \epsilon_{\rm R}(\mathbf{r},\omega) + i\epsilon_{\rm I}(\mathbf{r},\omega)$ is the complex permittivity function (or dielectric constant), whose real and imaginary part describes the dispersion and absorption of the EM environment, respectively. Note that when coupling to matter degrees of freedom (the TLS) is introduced later, the *full* electric field operator $\hat{\mathbf{E}}(\mathbf{r},\omega)$ also contains a contribution from these material components; the subscript F refers to the portion of the field which can be expressed in terms of the bosonic polariton operators, which can be found from a Fano diagonalization of a model of the passive medium as an oscillator bath [3, 4, 6]. The current source operator,

$$\hat{\mathbf{j}}_{\rm N}(\mathbf{r},\omega) = \omega\sqrt{\frac{\hbar\epsilon_0\epsilon_{\rm I}(\mathbf{r},\omega)}{\pi}}\,\hat{\mathbf{b}}(\mathbf{r},\omega), \tag{3}$$

is a noise current density, which preserves the fundamental field commutation relations. In the following, we will concentrate on the case of a (potentially absorptive) scattering structure, that is embedded in a spatial-homogeneous and non-absorptive background medium with $\epsilon(\mathbf{r},\omega) = n_{\rm B}^2$. Note, however, that this formalism can also take into account more non-trivial and practical photonic background media, such as photonic crystals or layered structures involving different substrates and waveguides, while fully accounting for dispersive properties.

The quantum Helmholtz equation (2) has the formal solution:

$$\hat{\mathbf{E}}_{\rm F}(\mathbf{r},\omega) = \frac{i}{\epsilon_0\omega}\int d^3s\,\mathbf{G}(\mathbf{r},\mathbf{s},\omega)\cdot\hat{\mathbf{j}}_{\rm N}(\mathbf{s},\omega), \tag{4}$$

where $\mathbf{G}(\mathbf{r},\mathbf{s},\omega)$ is the (classical) photonic Green function (tensor), defined from

$$\left[\boldsymbol{\nabla}\times\boldsymbol{\nabla}\times -\frac{\omega^2}{c^2}\epsilon(\mathbf{r},\omega)\right]\mathbf{G}(\mathbf{r},\mathbf{s},\omega) = \frac{\omega^2}{c^2}\mathbf{I}\delta(\mathbf{r}-\mathbf{s}), \tag{5}$$

together with suitable radiation conditions, i.e., the Silver-Müller radiation conditions, valid in the far-field:

$$\frac{\mathbf{r}}{|\mathbf{r}|} \times \boldsymbol{\nabla} \times \mathbf{G}(\mathbf{r}, \mathbf{s}, \omega) \rightarrow i n_{\mathrm{B}} \frac{\omega}{c} \mathbf{G}(\mathbf{r}, \mathbf{s}, \omega). \tag{6}$$

The electric field operator, $\hat{\mathbf{E}}_{\mathrm{F}}(\mathbf{r})$ is the sum over all mode indices ω, so that

$$\hat{\mathbf{E}}_{\mathrm{F}}(\mathbf{r}) = \int_0^\infty d\omega \hat{\mathbf{E}}_{\mathrm{F}}(\mathbf{r}, \omega) + \mathrm{H.c.} \tag{7}$$

The transverse part of the vector potential (which is manifestly gauge-invariant) $\hat{\mathbf{A}}_{\perp}(\mathbf{r})$ plays a fundamental role in the introduction of gauge-invariant coupling to a TLS, and is given by

$$\hat{\mathbf{A}}_{\perp}(\mathbf{r}) = \frac{1}{\epsilon_0} \int \frac{d\omega}{\omega^2} \int d^3 s \, \mathbf{G}^{\perp}(\mathbf{r}, \mathbf{s}, \omega) \cdot \hat{\mathbf{j}}_{\mathrm{N}}(\mathbf{s}, \omega), \tag{8}$$

where

$$\mathbf{G}^{\perp}(\mathbf{r}, \mathbf{s}, \omega) = \int d^3 r' \boldsymbol{\delta}^{\perp}(\mathbf{r} - \mathbf{r}') \cdot \mathbf{G}(\mathbf{r}', \mathbf{s}, \omega) \tag{9}$$

is the (left-sided) transverse part of the Green tensor, with $\boldsymbol{\delta}^{\perp}(\mathbf{r} - \mathbf{r}')$ being the transverse part of the Dirac delta distribution. Thus, the EM field operators are determined by the classical Green function, while the quantum character is fully encoded in the bosonic polariton operators.

The macroscopic QED formalism allows for rigorous quantization in arbitrary media, but, as it expresses the EM fields in terms of continuous frequency and spatially-dependent operators, it alone does not yet take advantage of the discrete nature of photonic resonances that occur in (e.g.) cavity structures. However, using QNM theory [7], one can indeed construct an efficient and concise mode decomposition of the field operators, which will be explained in more detail below. In addition to being valid for arbitrary dispersive and absorptive media, the QNM approach also recovers the important and practical limit of a non-absorptive dielectric medium [8], and is far more practical than a continuum approach for resonant structures that are well defined in terms of a few transverse modes of interest.

B. Classical field quasinormal mode theory

Next we present some basic aspects of the classical theory of QNMs that are needed for their quantization, including the Green function expansion and mode regularization.

We first introduce the concept of open cavity modes, which we refer to as QNMs [9–12]. Formally, the vector-valued QNM eigenfunctions, $\tilde{\mathbf{f}}_\mu(\mathbf{r})$, of a dispersive and three-dimensional scattering structure are solutions to the classical Helmholtz equation, Eq. (1) of the main text, which is solved with the Silver-Müller radiation condition, enforced in the far-field:

$$\frac{\mathbf{r}}{|\mathbf{r}|} \times \boldsymbol{\nabla} \times \tilde{\mathbf{f}}_\mu(\mathbf{r}) \rightarrow i n_{\mathrm{B}} \frac{\tilde{\omega}_\mu}{c} \tilde{\mathbf{f}}_\mu(\mathbf{r}). \tag{10}$$

As a consequence of the radiation conditions, the QNM eigenfrequencies $\tilde{\omega}_\mu = \omega_\mu - i\kappa_\mu/2$ are complex numbers, where ω_μ and κ_μ describe the resonance frequency and full width at half maximum of the associated QNM resonance, respectively. In addition, $\epsilon(\mathbf{r}, \tilde{\omega}_\mu)$ is the analytical continuation of the permittivity function into the complex plane. Note, in the following, we will assume that the QNMs are purely transverse (thus, we neglect any static/longitudinal QNM parts), so that the modal part of the Green function expansion is fully encoded in its transverse part, which is found to completely dominate for a wide range of dielectric and plasmonic cavities [13–17], including dimer plasmonic gaps, with gaps less than 1-nm [13]; indeed, this is also found to be the case with non-local effects included [14, 17].

Assuming completeness for the QNMs, and also based on various rigorous numerical checks for accurate QNM expansions for a wide range of cavity modes, the transverse part of the Green function for positions $\mathbf{r}_0$, $\mathbf{r}$ inside or near the resonator can be expanded in terms of the (transverse) QNMs [12, 18–20],

$$\mathbf{G}^{\perp}(\mathbf{r}_0, \mathbf{r}, \omega) = \sum_\mu A_\mu(\omega) \, \tilde{\mathbf{f}}_\mu(\mathbf{r}_0) \, \tilde{\mathbf{f}}_\mu(\mathbf{r}), \tag{11}$$

where $A_\mu(\omega)$ is a frequency-dependent QNM expansion coefficient,

$$A_\mu(\omega) = \frac{\omega}{2(\tilde{\omega}_\mu - \omega)}, \tag{12}$$

and the QNMs are appropriately normalized. The QNM expansion uses an unconjugated product of vector fields,

which is essential to capture important QNM phase effects.

Formally speaking, the expansion in terms of QNMs in Eq. (11) should only be used *inside* of the scatterer (resonator) geometry. However, when the QNMs dominate the EM response for a given frequency bandwidth, the use of a strictly QNM expansion is also highly accurate at spatial locations in the near-field of the resonator (i.e., where a material dipole emitter could be placed in a cavity-QED setting). One consequence of this approximation is that "background" terms, which arise from the continuous background modes and are required to solve Maxwell's equations at locations far from the resonator geometry, are neglected [20]. These terms are necessary to capture background field couplings to material emitter excitations, which give rise to the usual spontaneous emission into non-cavity modes. In USC, such terms are often very small, and thus we neglect them going forward.

In the following, any additional longitudinal parts, $\mathbf{G}^{\parallel}(\mathbf{r}_0, \mathbf{r}, \omega)$, are formally included as weakly contributing parts to the total Green function. In many QNM calculations for resonant cavity systems including metallic dimers or hybrid dielectric-metal resonators, the response is typically completely described by one or just several QNMs [12, 13, 21–23] (excluding transverse background terms, which as mentioned above are often small in USC), which is one of the main advantages of using a *discrete* mode quantization scheme.

We emphasize that the expansion in Eq. (11) is assumed to be performed at spatial positions within or near the cavity region, which we refer to as the "system" region (cf. Fig. 1(a) of the main text). The actual bounds of the system region are not important, and do not enter into the theory (at least when the background media has no absorption), provided that coupling to active material emitters occurs within the system region, and that the far-field is contained in the background region.

To fully determine the EM field operators in the system region, such as the vector potential in Eq. (8), a Green function expansion for its second spatial entry outside the system is also required. At locations far outside the system, practically the QNMs must be *regularized* as they no longer directly form a good basis for the Green function [21, 24, 25]. The regularizations we use are based on fundamental Green identities and require either: (i) the QNMs *inside* the resonator through a form of the Dyson equation [21], or (ii) the QNMs at a boundary enclosing the resonator region through a near-field to far-field transformation [26], which exploits the field equivalence principle [27]. Utilizing these regularizations, one can formulate the transverse Green function for positions (i.e., the second spatial argument of the Green function) outside the system as

$$\mathbf{G}^{\perp}(\mathbf{r}, \mathbf{R}, \omega) = \sum_{\mu} A_{\mu}(\omega) \tilde{\mathbf{f}}_{\mu}(\mathbf{r}) \tilde{\mathbf{F}}_{\mu}(\mathbf{R}, \omega), \tag{13}$$

where $\tilde{\mathbf{F}}_{\mu}(\mathbf{R}, \omega)$ represents the *regularized QNM* functions, which can be obtained from the Dyson equation approach (which we use going forward) to yield [20]:

$$\tilde{\mathbf{F}}_{\mu}(\mathbf{R}, \omega) = \int d^3 s \Delta\epsilon(\mathbf{s}, \omega) \mathbf{G}_{\mathrm{B}}(\mathbf{R}, \mathbf{s}, \omega) \cdot \tilde{\mathbf{f}}_{\mu}(\mathbf{s}), \tag{14}$$

and $\Delta\epsilon(\mathbf{s}, \omega) = \epsilon(\mathbf{r}, \omega) - n_{\mathrm{B}}^2$ is the permittivity difference (defining the cavity) and $\mathbf{G}_{\mathrm{B}}(\mathbf{R}, \mathbf{s}, \omega)$ is the background Green function, which solves Eq. (5) with $\epsilon(\mathbf{r}, \omega) = n_{\mathrm{B}}^2$. Here, in line with our earlier discussion, we have again neglected non-modal contributions to the Green function coming from background contributions [20].

We remark that there are different forms of the QNM expansion coefficient $A_{\mu}(\omega)$ that one could choose, and the difference in choosing which form to use corresponds to a different set of ways to make a *few-mode approximation*; however, when all modes are included, either choice should lead to equivalent results. When considering the single-mode limit, by use of a Green function identity, either form leads to equivalent results [28]. More specifically, we will see that the use of this identity leads to two different representations of our theory: a *spatially unspecified* form, and a *spatially specified* form (defined later in the text) which picks out a specific location in space, and thus depends explicitly on the phase of the QNM at the specified location. For the spatially unspecified form, the choice of $A_{\mu}(\omega)$ does in fact lead to different results, but we show that the spatially specified form is generally needed to obtain the correct result. For very large light-matter couplings (i.e., the deep-strong coupling regime, where the dipole-cavity coupling is nearly equal to or larger than the bare frequencies of the system), different coefficient forms can lead to different results even in the spatially specified representation [28]. Here, other issues can occur (e.g., quasistatic effects) as well, so we preclude these extreme regimes from consideration in the (ultimately single-mode) quantized QNM model we present in this work.

For our considered case of a homogeneous background, there is an explicit analytical form for $\mathbf{G}_{\mathrm{B}}(\mathbf{r}, \mathbf{r}', \omega)$, which is given through [29]

$$\mathbf{G}_{\mathrm{B}}(\mathbf{r}, \mathbf{r}', \omega) = -\frac{\delta(\mathbf{X})}{3n_{\mathrm{B}}^2}\mathbf{I} + \frac{k_0^2 \exp(ik_{\mathrm{B}}X)}{4\pi X}\left[\left(1 + \frac{ik_{\mathrm{B}}X - 1}{k_{\mathrm{B}}^2 X^2}\right)\mathbf{I} + \left(\frac{3 - 3ik_{\mathrm{B}}X - k_{\mathrm{B}}^2 X^2}{k_{\mathrm{B}}^2 X^2}\right)\mathbf{n}_{\mathbf{X}}\mathbf{n}_{\mathbf{X}}\right], \tag{15}$$

with $\mathbf{X} = \mathbf{r} - \mathbf{r}' = \mathbf{n}_{\mathbf{X}} X$, $X = |\mathbf{X}|$, $k_0 = \omega/c$ and $k_{\mathrm{B}} = n_{\mathrm{B}} k_0$. The presented theoretical framework also covers other

background structures, e.g. a photonic crystal slab structure with an embedded waveguide. In that case, most of the output from the cavity (usually realized by missing holes in the photonic crystal) will be directed into the direction of the (quasi) one-dimensional waveguide, where the respective Green function $\mathbf{G}_{\rm wg}$ is known analytically [30].

Summarizing the different contributions to the total Green function, we have

$$\mathbf{G}(\mathbf{r}_0,\mathbf{r},\omega)=\sum_\mu A_\mu(\omega)\tilde{\mathbf{f}}_\mu(\mathbf{r}_0)\tilde{\mathbf{f}}_\mu(\mathbf{r})+\mathbf{G}^{\parallel}(\mathbf{r}_0,\mathbf{r},\omega), \tag{16}$$

for $\mathbf{r}$ (or $\mathbf{r}_0$) at positions *inside* the system region and

$$\mathbf{G}(\mathbf{r}_0,\mathbf{R},\omega)=\sum_\mu A_\mu(\omega)\tilde{\mathbf{f}}_\mu(\mathbf{r}_0)\tilde{\mathbf{F}}_\mu(\mathbf{R},\omega)+\mathbf{G}^{\parallel}(\mathbf{r}_0,\mathbf{R},\omega), \tag{17}$$

for $\mathbf{R}$ at positions *outside* the system region. These are combined into a single expression in the main text, in terms of $\tilde{\mathbf{F}}'_\mu$.

C. Quantized quasinormal modes and reservoir modes

We next summarize and extend previous theoretical work on the expansion of the EM field operators and Hamiltonian in terms of QNMs (see Refs. [4, 7, 8, 15]), which is an essential step for the derivation of the TLS-field interaction in the USC regime described in Sec. I D.

In particular, we will concentrate on the transverse part of the vector potential $\hat{\mathbf{A}}_\perp(\mathbf{r})$ as well as the longitudinal part of the electric field operator $\hat{\mathbf{E}}_{\rm F}^{\parallel}(\mathbf{r})$, as these operators are the ones which appear in the minimal coupling Hamiltonian which couples the fields to the TLS [4]. While one may naively expect the non-radiative processes (when absorption is present) to be entirely captured by the longitudinal part of the EM fields, there are also significant non-radiative decay processes associated with the (transverse) QNM part, since the coupling of the EM field and the medium is present in both the transverse and longitudinal fields. Indeed, all plasmonic QNMs have non-radiative contributions through Ohmic heating, which can be used to quantitatively describe the non-radiative beta factor (quenching), as well as the radiative beta factor (or quantum efficiency) [21]. This is sometimes poorly understood in the plasmonics community, but both modal contributions (radiative and nonradiative) scale precisely with the usual Purcell factor mode scaling, $\propto Q/V_{\rm eff}$.

In previous works involving quantized QNMs, a *spatially unspecified* scheme was used to construct the QNMs, where the quantization involves integration over all spatial regions, and does not pick out a specific location. Additionally in this work, by use of a Green function identity, we can move to a *spatially specified* representation by picking out a specific location of interest (e.g., the location of the dipole), which we will show is necessary to identify the correct frequency-dependence of the ME decay rates of the cavity in the USC and broadband dissipative regimes. This is discussed in Sec. I C 3.

1. *Projection of discrete oscillator subspace from continuum polariton excitations of EM fields and the medium*

,/jTo obtain a representation of the EM fields in terms of quantized QNMs and residual reservoir components, we employ a recently proposed projection approach [4, 31], adapted from previous QNM quantization schemes [7]. Specifically, we write the medium polariton operators as

$$\hat{\mathbf{b}}(\mathbf{r},\omega)=\sum_\mu \mathbf{L}^*_\mu(\mathbf{r},\omega)\hat{a}_\mu+\hat{\mathbf{c}}(\mathbf{r},\omega), \tag{18}$$

where we have defined quantized discrete operators

$$\hat{a}_\mu=\int d^3r\int d\omega_{\rm m}\mathbf{L}_\mu(\mathbf{r},\omega)\cdot\hat{\mathbf{b}}(\mathbf{r},\omega), \tag{19}$$

and $\hat{\mathbf{c}}(\mathbf{r},\omega)$ is a residual reservoir operator, defined implicitly by Eq. (18). For the discrete operators to correspond to distinct (quasi-)modes, we require $[\hat{a}_\mu,\hat{a}_\nu]=\delta_{\mu\nu}$, which implies

$$\int d^3r\int d\omega\mathbf{L}_\mu(\mathbf{r},\omega)\cdot\mathbf{L}^*_\nu(\mathbf{r},\omega)=\delta_{\mu\nu}. \tag{20}$$

Importantly, by construction, the discrete operators $\hat{a}_\mu^{(\dagger)}$ commute with the continuum operators $\hat{\mathbf{c}}^{(\dagger)}(\mathbf{r},\omega)$. As such, we can then decompose the field Hamiltonian $\hat{H}_{\rm em}$ into discrete, continuum, and interaction parts as follows:

$$\hat{H}_{\rm em}^{\rm discr} = \sum_{\mu\nu} \hbar\chi_{\mu\nu}\hat{a}_\mu^\dagger \hat{a}_\nu, \tag{21a}$$

$$\hat{H}_{\rm em}^{\rm cont} = \int d^3r \int d\omega \hbar\omega \hat{\mathbf{c}}^\dagger(\mathbf{r},\omega)\cdot\hat{\mathbf{c}}(\mathbf{r},\omega), \tag{21b}$$

$$\hat{H}_{\rm em}^{\rm int} = \sum_\mu \int d^3r \int d\omega\hbar\omega \mathbf{L}_\mu(\mathbf{r},\omega)\cdot\hat{\mathbf{c}}(\mathbf{r},\omega)\hat{a}_\mu^\dagger + {\rm H.c.}, \tag{21c}$$

where

$$\chi_{\mu\nu} = \int d^3r \int d\omega_{\rm m}\omega \mathbf{L}_\mu(\mathbf{r},\omega)\cdot\mathbf{L}_\nu^*(\mathbf{r},\omega). \tag{21d}$$

Note that the reservoir continuum operators in general have non-local commutation relations:

$$[\hat{\mathbf{c}}(\mathbf{r},\omega),\, \hat{\mathbf{c}}^\dagger(\mathbf{r}',\omega')] = \mathbf{I}\delta(\mathbf{r}-\mathbf{r}')\delta(\omega-\omega') - \sum_\mu \mathbf{L}_\mu^*(\mathbf{r},\omega)\mathbf{L}_\mu(\mathbf{r}',\omega'), \tag{22}$$

though for the case of quantized QNMs, we show in Sec. V that the second term of Eq. (22) can be neglected when deriving the quantum ME, and the reservoir operators are effectively local under the Markov approximation.

Up to now, this construction is completely general. Additionally, to obtain a mode expansion for the transverse vector potential of the form $\hat{\mathbf{A}}_\perp(\mathbf{r}) = \hat{\mathbf{A}}_{\rm discr}^\perp(\mathbf{r}) + \hat{\mathbf{A}}_{\rm R}^\perp(\mathbf{r})$, where

$$\hat{\mathbf{A}}_{\rm discr}^\perp = \sum_\mu \sqrt{\frac{\hbar}{2\epsilon_0\chi_{\mu\mu}}}\tilde{\mathbf{f}}_\mu^{\rm s}(\mathbf{r})\hat{a}_\mu + {\rm H.c.}, \tag{23}$$

and

$$\hat{\mathbf{A}}_{\rm R}^\perp(\mathbf{r}) = \int {\rm d}^3r' \int \frac{d\omega}{\omega}\sqrt{\frac{\hbar\epsilon_I(\mathbf{r}',\omega)}{\epsilon_0\pi}}\,\mathbf{G}^\perp(\mathbf{r},\mathbf{r}',\omega)\cdot\hat{\mathbf{c}}(\mathbf{r}',\omega) + {\rm H.c.}, \tag{24}$$

which, in terms of $\hat{\mathbf{A}}_{\rm discr}^\perp(\mathbf{r})$, is similar in form to more usual normal mode expansions, we define a corresponding (quasi-)mode profile:

$$\tilde{\mathbf{f}}_\mu^{\rm s}(\mathbf{r}) = \sqrt{\frac{2\chi_{\mu\mu}}{\pi}}\int d^3r' \int \frac{d\omega}{\omega}\sqrt{\epsilon_I(\mathbf{r}',\omega)}\mathbf{G}^\perp(\mathbf{r},\mathbf{r}',\omega)\cdot\mathbf{L}_\mu^*(\mathbf{r}',\omega). \tag{25}$$

2. *Construction of quantized quasinormal modes*

We now move from this general construction of a discrete oscillator basis to a description in terms of *quantized QNMs*. To do so, we choose the projection functions to satisfy [4]:

$$\mathbf{L}_\mu(\mathbf{r},\omega) = \sum_\nu \left[S^{-\frac{1}{2}}\right]_{\mu\nu}\sqrt{\frac{2\omega_\nu\epsilon_I(\mathbf{r},\omega)}{\pi}}\frac{A_\nu(\omega)}{\omega}\tilde{\mathbf{F}}'_\nu(\mathbf{r},\omega), \tag{26}$$

where $\tilde{\mathbf{F}}'_\nu(\mathbf{r},\omega) = \tilde{\mathbf{f}}_\nu(\mathbf{r})$ for $\mathbf{r}$ inside the system region (i.e., the QNM mode profile), and $\tilde{\mathbf{F}}'_\nu(\mathbf{R},\omega) = \tilde{\mathbf{F}}_\nu(\mathbf{R},\omega)$ for $\mathbf{R}$ outside the system region (i.e., the regularized QNM). The former $\tilde{\mathbf{f}}_\nu(\mathbf{r})$ are frequency independent and spatially divergent, and the latter $\tilde{\mathbf{F}}_\nu(\mathbf{R},\omega)$ are frequency-dependent and spatially convergent (i.e., well behaved in the far field).

The factor $S_{\mu\nu}$ is a symmetrizing matrix (closely connected to the radiative and non-radiative loss of the QNMs), which is found by enforcing Eq. (20),

$$S_{\mu\nu} = \frac{2\sqrt{\omega_\mu\omega_\nu}}{\pi}\int\frac{d\omega}{\omega^2}\int d^3r\epsilon_I(\mathbf{r},\omega)A_\mu(\omega)A_\nu^*(\omega)\tilde{\mathbf{F}}'_\mu(\mathbf{r},\omega)\cdot\tilde{\mathbf{F}}'^*_\nu(\mathbf{r},\omega). \tag{27}$$

We refer to this form of the symmetrizing matrix as the *spatially unspecified form*, as it does not pick out any specific position where the QNMs are evaluated, in contrast to the spatially specified form we identify in Sec. I C 3 for the

single-mode limit.

Then, inserting the definition of the projection functions in Eq. (26) into the mode expansion coefficients from Eq. (25), we see that the relationship between the field expansion functions $\tilde{\mathbf{f}}^{\rm s}_\mu(\mathbf{r})$ and the QNM mode profiles is

$$\tilde{\mathbf{f}}^{\rm s}_\mu(\mathbf{r}) = \sum_\nu \tilde{\mathbf{f}}_\nu(\mathbf{r}) \left[S^{\frac{1}{2}}\right]_{\nu\mu} \sqrt{\frac{\chi_{\mu\mu}}{\omega_\nu}}, \tag{28}$$

and they take the form of symmetrized QNM mode profile functions, recovering previous work [7]. As such, we can identify $\hat{\mathbf{A}}^\perp_{\rm discr} = \hat{\mathbf{A}}^\perp_{\rm QNM}$, in terms of quantized QNMs. Explicitly, for $\mathbf{r}$ within the system region,

$$\hat{\mathbf{A}}^\perp_{\rm QNM}(\mathbf{r}) = \sum_\mu \sqrt{\frac{\hbar}{2\epsilon_0 \chi_{\mu\mu}}} \tilde{\mathbf{f}}^{\rm s}_\mu(\mathbf{r}) \hat{a}_\mu + {\rm H.c.}, \tag{29}$$

and, in fact, due to our definition of the QNM projection functions $\mathbf{L}_\mu(\mathbf{r},\omega)$, we have simply $\hat{\mathbf{A}}^\perp_{\rm R}(\mathbf{r}) = 0$ for the case of QNMs (see Sec. III for a derivation). Note that the vanishing of the background component is unique to the transverse potential—for example, $\hat{\mathbf{E}}^\perp_{\rm F,B} \neq 0$. Moreover, it is a consequence of our approximation of neglecting non-modal transverse background contributions to the Green function expansion, which, as previously mentioned, would be necessary to include to model things like nonresonant background radiation loss of emitters to non-QNM modes on an *ab initio* basis, if in a regime where such terms are substantial.

The difference between the quantization of QNMs here and previous work [7, 8, 32, 33] is twofold: (i) We define the quantized QNM expansion in terms of the transverse vector potential (as opposed to the electric field operator), as the vector potential plays the more fundamental role in the gauge-invariant theory of macroscopic QED, as it is used to define the Hamiltonian which remains gauge-invariant upon truncation of material levels to a finite basis, which is especially important to ensure gauge-invariant results in the USC regime [4, 34]. This definition is more convenient, as, for this choice, $\hat{\mathbf{A}}^\perp_{\rm R} = 0$, which allows for straightforward transformations between gauges in terms of the quantized QNM operators. As certain gauges are more convenient in specific situations (for example, the Coulomb gauge is more convenient in the case of time-dependent couplings to matter [4, 35]), the ability to transform between gauges with ease is a substantial benefit of our scheme; (ii) We define the mode expansion in terms of the $\chi_{\mu\mu}$ parameters, as opposed to the bare QNM frequency ω_μ, as in the single-mode limit, $\chi_{\mu\mu}$ is the coefficient of the bare cavity QNM resonance that occurs in the Hamiltonian [see Eq. (21a)]. Note that for high-Q resonances (and in practice, even modest Q factors ~ 10 encountered in plasmonics), the poles in the projection functions $\mathbf{L}_\mu(\mathbf{r},\omega)$ centered around ω_μ allow for the ω factor in Eq. (21d) to be approximated as ω_μ, recovering $\chi_{\mu\mu} \approx \omega_\mu$. In practice, we do this for all our calculations and equations in the main text.

Despite these differences, the fundamental approach of our quantization scheme here and the associated numerical techniques for calculating the associated parameters, including the symmetrizing matrix $S_{\mu\nu}$ (i.e., in the spatially unspecified representation) and its positive definiteness, remain the same as in previous works. Additionally, we note that the choice of defining the QNM expansion in terms of the transverse vector potential is not essential, and one can also use a definition in terms of the transverse electric field (as in previous works), and we do this in Sec. IV. A formulation with respect to the electric field is more convenient in particular if using the dipole gauge, which can be sometimes be preferable when calculating TLS observables [36] and formulating input-output theories [37].

Note that, in the general case of lossy materials, and in the spatially unspecified form, we have separate contributions from radiative and non-radiative losses. The term

$$S^{\rm nrad}_{\mu\nu} = \frac{2}{\pi} \int d\omega \frac{\sqrt{\omega_\mu \omega_\nu}}{\omega^2} A_\mu(\omega) A^*_\nu(\omega) S^{\rm nrad}_{\mu\nu}(\omega), \tag{30}$$

where

$$S^{\rm nrad}_{\mu\nu}(\omega) = \int_{\rm V_{in}} d^3 r \epsilon_I(\mathbf{r},\omega) \tilde{\mathbf{f}}_\mu(\mathbf{r}) \cdot \tilde{\mathbf{f}}^*_\nu(\mathbf{r}), \tag{31}$$

reflects the non-radiative loss contribution through a QNM overlap integral over the absorptive region (the scattering geometry $\rm V_{in}$), and

$$S^{\rm rad}_{\mu\nu} = \frac{2}{\pi} \int d\omega \frac{\sqrt{\omega_\mu \omega_\nu}}{\omega^2} A_\mu(\omega) A^*_\nu(\omega) S^{\rm rad}_{\mu\nu}(\omega), \tag{32}$$

where

$$S^{\rm rad}_{\mu\nu}(\omega) = \frac{n_{\rm B} c}{\omega} \oint_{\mathcal{S}_\infty} dA_{\mathbf{s}} \tilde{\mathbf{F}}_\mu(\mathbf{s},\omega) \cdot \tilde{\mathbf{F}}^*_\nu(\mathbf{s},\omega) \tag{33}$$

describes the radiative loss processes through a power flow expression at a far-field surface S_∞; here the Silver-Müller radiation conditions are approximately valid (compared to the strict relation at $|\mathbf{s}| \to \infty$). As mentioned before, the QNM part contains both non-radiative and radiative loss processes. Obviously for a lossless material system, then we only have radiative contributions, a limit that is fully recovered for any finite-size real dielectric resonator [8]. From a practical viewpoint, one can approximate the radiative and non-radiative symmetrization factors through a resonant pole approximation, around the peaks of the appearing Lorentzian functions (due to the pole in the QNM expansion coefficients) [24].

Moving on to the longitudinal electric field operator, the QNM-basis contribution becomes

$$\hat{\mathbf{E}}^{\parallel}_{\rm F,QNM}(\mathbf{r}) = i\sum_\mu \sqrt{\frac{\hbar\chi_{\mu\mu}}{2\epsilon_0}}\tilde{\mathbf{f}}^{\parallel}_\mu(\mathbf{r})\hat{a}_\mu + {\rm H.c.}, \tag{34}$$

where we have defined the projected longitudinal "mode" profiles as

$$\tilde{\mathbf{f}}^{\parallel}_\mu(\mathbf{r}) = \int d^3r' \int d\omega \sqrt{\frac{2\epsilon_I(\mathbf{r}',\omega)}{\pi\chi_{\mu\mu}}}\mathbf{G}^{\parallel}(\mathbf{r},\mathbf{r}',\omega)\cdot\mathbf{L}^*_\mu(\mathbf{r}',\omega), \tag{35}$$

and the reservoir contribution is

$$\hat{\mathbf{E}}^{\parallel}_{\rm F,B}(\mathbf{r}) = i\sqrt{\frac{\hbar}{\pi\epsilon_0}}\int_0^\infty d\omega \int d^3s\sqrt{\epsilon_I(\mathbf{s},\omega)}\mathbf{G}^{\parallel}(\mathbf{r},\mathbf{s},\omega)\cdot\hat{\mathbf{c}}(\mathbf{s},\omega) + {\rm H.c.} \tag{36}$$

Note that $\tilde{\mathbf{f}}^{\parallel}_\mu(\mathbf{r})$ are not mode profiles in a typical sense, but rather spatially-dependent and longitudinal expansion coefficient associated with the quantized QNMs when the full longitudinal electric field is decomposed in the QNM-reservoir basis. Although the QNMs we use are transverse, the mode projection functions $\mathbf{L}_\mu(\mathbf{r},\omega_\mu)$ are not strictly transverse over all space, and as such, retain small longitudinal components. In a regime where the transverse QNMs dominate over the total EM fields, we expect the contribution of these longitudinal components to be negligible (as can easily be confirmed numerically).

In addition to the field decompositions, we also note that the discrete-continuum Hamiltonian in Eq. (21) can also be understood in terms of this QNM-reservoir separation, with $\hat{H}^{\rm em}_{\rm discr} = \hat{H}^{\rm em}_{\rm QNM}$, $\hat{H}^{\rm em}_{\rm cont} = \hat{H}^{\rm em}_{\rm R}$, and $\hat{H}^{\rm em}_{\rm int} = \hat{H}^{\rm em}_{\rm QNM-R}$. The last interaction term can also be written as (cf. Ref. [15] for details)

$$H^{\rm em}_{\rm QNM-R} = \hbar\sum_\mu \int_0^\infty d\omega \int d^3r\, \mathbf{g}_\mu(\mathbf{r},\omega)\cdot\hat{\mathbf{c}}(\mathbf{r},\omega)\hat{a}^\dagger_\mu + {\rm H.c.}, \tag{37}$$

where

$$\mathbf{g}_\mu(\mathbf{r},\omega) = \sum_\nu \left[S^{-\frac{1}{2}}\right]_{\mu\nu}\sqrt{\frac{2\omega_\nu\epsilon_I(\mathbf{r},\omega)}{\pi}}\frac{B_\nu(\omega)}{\omega}\tilde{\mathbf{F}}'_\nu(\mathbf{r},\omega); \tag{38}$$

this has the same form as the projection function $\mathbf{L}_\mu(\mathbf{r},\omega)$, but with

$$B_\nu(\omega) = -(\tilde{\omega}_\nu - \omega)A_\nu(\omega) \tag{39}$$

replacing $A_\nu(\omega)$; i.e., with the simple pole at the complex QNM frequency removed. For the choice of coefficient we use in this work, Eq. (12), one simply has $B_\nu(\omega) = -\omega/2$ for all ν.

Our model can be regarded as an *ab initio* system-reservoir theory, where the operators $\hat{\mathbf{c}}(\mathbf{r},\omega)$ and associated states take on the role of the (residual) reservoir modes and $\hat{a}_\mu$ are the system QNM modes. The inherent QNM decay rate κ_μ is now implicitly included in the more general coupling $\mathbf{g}_\mu(\mathbf{r},\omega)$ through evaluation for frequencies around ω_μ, i.e., for a single QNM μ and no active emitter coupling, $\int {\rm d}^3r\, |\mathbf{g}_\mu(\mathbf{r},\omega_\mu)|^2 \approx \kappa_\mu/(2\pi)$. This function, once transformed to the spatially-specified representation, allows us to calculate frequency-dependent decay rates corresponding to optically active transitions of the light-matter Hamiltonian in the USC regime, without any phenomenological assumptions on the form of the system-reservoir interaction. This will be discussed in more detail in Sec. I F 3.

3. *Spatially specified form in the single-mode approximation*

In the single-mode case (taking $\mu = c$), we can also move to a spatially specified form of the $S_c = S_{cc}$ quantization parameter, which, as we show later, is necessary to obtain the correct frequency-dependent modifications to the ME in the presence of coupling to active dipole TLS coupling [28]. This can be done by making use of the fundamental

Green function relation [1]:

$$\int d^3r\epsilon_I(\mathbf{r})\mathbf{G}^\perp(\mathbf{r}_1,\mathbf{r},\omega)\cdot\mathbf{G}^{\perp *}(\mathbf{r},\mathbf{r}_2,\omega)=\mathrm{Im}\{\mathbf{G}^\perp(\mathbf{r}_1,\mathbf{r}_2,\omega)\} \tag{40}$$

for any $\mathbf{r}_1$ and $\mathbf{r}_2$. Thus, taking Eq. (16) and Eq. (17) in the single-mode limit, letting $\mathbf{r}_1=\mathbf{r}_2$, and taking the dot product on the right and left with an arbitrary real unit vector $\mathbf{n}$, we obtain the relation

$$\int d^3r\epsilon_I(\mathbf{r},\omega)|A_c(\omega)\tilde{\mathbf{F}}_c'(\mathbf{r},\omega)|^2=\mathrm{Im}\{A_c(\omega)e^{i2\phi_1}\}, \tag{41}$$

which is clearly dependent on the QNM phase at the specified location projected onto the arbitrary vector, $\phi_1=\arg\{\mathbf{n}\cdot\tilde{\mathbf{f}}_c(\mathbf{r}_1)\}$. We remark that this expression could potentially be generalized by considering $\mathbf{r}_1\neq\mathbf{r}_2$, which may be a starting point for an extension to the case where one has many dipoles with substantially different QNM phases at their locations. We then have

$$\mathrm{Im}\{A_c(\omega)e^{i2\phi_1}\}=\frac{\omega\kappa_c\cos{(2\phi_1)}}{4\left[(\omega-\omega_c)^2+\kappa_c^2/4\right]}\zeta_c(\phi_1,\omega), \tag{42}$$

where

$$\zeta_c(\phi_1,\omega)=1-2Q_c\tan{(2\phi_1)}\left(\frac{\omega}{\omega_c}-1\right) \tag{43}$$

is a factor which depends on the intrinsic QNM phase at the chosen location, and determines the deviation from a Lorentzian lineshape (modulated by a linear frequency factor) of the spontaneous emission rate of a dipole with resonant frequency $\omega_0=\omega$ weakly coupled to a single cavity mode [28]. Now taking the single-mode limit of Eq. (27), we find, in the spatially specified representation,

$$S_c(\mathbf{n},\mathbf{r}_1)=\frac{\kappa_c\cos{(2\phi_1)}}{2\pi}\int\frac{d\omega}{\omega}\frac{\zeta_c(\phi_1,\omega)}{(\omega-\omega_c)^2+\kappa_c^2/4}. \tag{44}$$

Assuming the integral to be sharply peaked around the resonance $\omega=\omega_c$, we can make a pole approximation by letting $\omega\approx\omega_c$ in the factor that appears in the denominator (consistent with previous evaluations of the S quantization matrix [15]), and extend the limits of the frequency integral to $\pm\infty$, obtaining:

$$S_c(\mathbf{n},\mathbf{r}_1)\approx\cos{(2\phi_1)}. \tag{45}$$

We remark that this approximation breaks down when $|\tan{(2\phi_1)}|\gg 1$; thus, we should exclude this representation in any regions where $\phi_1\approx\pi/4$ (or more generally $\pm\pi/4(2m+1)$, with $m=0,1,2,\cdots$). Moreover, when considering broadband coupling to matter within the Markov approximation, the resultant ME can (as we will see later) have unphysical negative decay rates for any QNM phase that is not sufficiently small, so more broadly we restrict ourselves to $|\phi_1|\ll 1$.

It is important to note that, in this representation, $S_c(\mathbf{n},\mathbf{r}_1)$ *depends explicitly on the QNM phase at the position that has been specified*, and as such, will generally give different results that the spatially unspecified form given in the previous subsection. Additionally, this form does not allow one to identify radiative and non-radiative contributions separately as a function of position (i.e., of a placed dipole). These representations are different ways of making a single-mode approximation within the same underlying quantization of the QNMS, with the spatially-specified representation arising from imposing that the Green function identity in Eq. (40) hold for a Green function described approximately by just a single QNM.

D. Generalized minimal coupling replacement

In recent years, it has become well-established that the usual approach to truncate the minimal coupling Hamiltonian giving light-matter interactions can lead to inconsistent gauge-dependent predictions when in the USC regime, and that gauge-invariance can be restored by means of an appropriate generalization of the minimal coupling procedure [4, 38–42]. In Ref. [4] we have recently shown how to derive arbitrary-gauge Hamiltonians consistent with gauge-invariance in the truncated space for both material and mode truncation. Here we briefly show how to write down the correctly-truncated Coulomb and dipole gauge Hamiltonians under the case of material truncation; since our separation of QNM and reservoir parts of the EM fields preserves the entire spatio-frequency structure of their expansions, mode truncation concerns are not directly applicable here.

Before introducing interactions with the active matter degrees of freedom and the EM fields, we assume an initial

uncoupled (or bare) Hamiltonian of light and matter, which takes the form:

$$\hat{H}_{\rm bare} = \hat{H}_0 + \hat{H}_{\rm em}, \tag{46}$$

where $\hat{H}_{\rm em}$ is defined in Eq. (1) and $\hat{H}_0$ is an essentially arbitrary *effective matter* Hamiltonian, but ultimately should have an energy spectrum with an optical transition dipole moment between two states which are well-separated in energy from all other transitions of its spectrum to allow for a TLS approximation; H_0 thus contains any Coulomb interactions between the constituent particles of the matter system. However, we stress that interactions with the medium-assisted field, transverse or longitudinal, have not yet been included. It should be noted that medium-assisted longitudinal fields can play a role in modifying the bare ($\hat{H}_0$) material particle system energy, in a way not easily captured by our formalism [43]. Following the standard approach, we assume this effect to be already incorporated in the definition of $\hat{H}_0$, and we include the longitudinal medium-assisted terms later for completeness.

We then introduce the projector operator for a single TLS:

$$\hat{P} = |e\rangle\langle e| + |g\rangle\langle g|, \tag{47}$$

where $|e\rangle$ and $|g\rangle$ are the two states ("excited" and "ground", respectively) which constitute the two-level approximation for the matter Hamiltonian; these have energy separation $\hbar\omega_0$, such that

$$\hat{P}\hat{H}_0\hat{P} = \frac{\hbar\omega_0}{2}\hat{\sigma}_z. \tag{48}$$

The only information about the TLS which is required, in addition to its energy level separation, is its transition dipole moment, defined from:

$$\langle e|\hat{\mathbf{d}}|e\rangle = \langle g|\hat{\mathbf{d}}|g\rangle = 0, \tag{49a}$$

$$\langle e|\hat{\mathbf{d}}|g\rangle = \langle g|\hat{\mathbf{d}}|e\rangle = \mathbf{d}, \tag{49b}$$

where $\hat{\mathbf{d}} = \sum_\alpha q_\alpha \hat{\mathbf{r}}_\alpha = \mathbf{d}\hat{\sigma}_x$ is the dipole operator and $\mathbf{d}$ is the dipole moment (assumed real). Since Eqs. (49a) and (49b) are satisfied so long as the TLS has parity symmetry, our results are general, and thus apply to any dipole emitter system. Figure S1 shows a schematic of this setup.

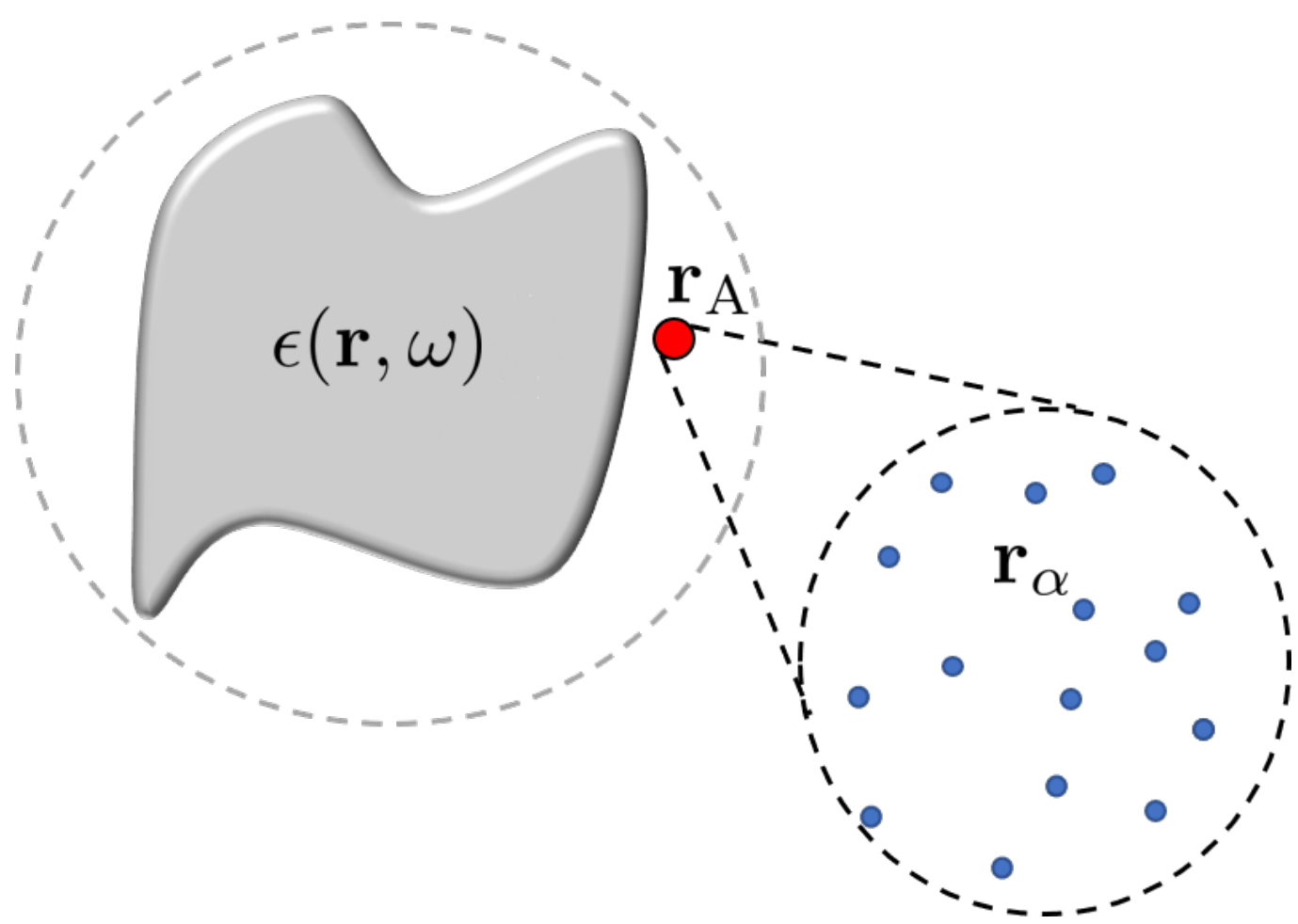

Figure S1. Schematic of an arbitrary shaped scattering structure with (complex) permittivity $\epsilon(\mathbf{r},\omega)$, which is located near a cloud of charged particles with positions $\mathbf{r}_\alpha$ (blue dots). The charged particles form an effective particle at the center of mass position $\mathbf{r}_{\rm A}$ (red dot). The grey dashed circle reflects the region of the "system", where the QNMs form a good basis for the field expansion (cf. Fig. 1 from the main text). Note that in this work we let $\mathbf{r}_{\rm A} = \mathbf{0}$.

Including dipole-field interactions, it has been shown recently that the Hamiltonian which preserves gauge-invariance under material truncation within the framework of macroscopic QED and quantized QNM theory is [4]:

$$\hat{\mathcal{H}} = \hat{\mathcal{V}}\hat{H}_{\rm em}\hat{\mathcal{V}}^\dagger + \hat{\mathcal{U}}\hat{\mathcal{H}}_0\hat{\mathcal{U}}^\dagger + \hat{\mathcal{H}}_\parallel, \tag{50}$$

where we use calligraphic letters to denote variables truncated with respect to the material degrees of freedom. The choice of gauge under which $\hat{\mathcal{H}}$ is to be realized is determined by the unitary operators $\hat{\mathcal{V}}^{(\dagger)}$ and $\hat{\mathcal{U}}^{(\dagger)}$. As $\hat{\mathcal{U}}^{(\dagger)}$ in particular is expressed directly as a function of the truncated dipole operator (proportional to the position operator),

the minimal-coupling replacement is self-consistently implemented in the two-level basis, preserving gauge-invariance.

Here, $\hat{\mathcal{H}}_{\parallel} = -\mathbf{d}\cdot\hat{\mathbf{E}}_{\rm F}^{\parallel}(\mathbf{0})\hat{\sigma}_x$ is the manifestly gauge-invariant term corresponding to longitudinal electric field coupling to the dipole. The coupling between the transverse EM fields and the dipole is given by the unitary operators $\hat{\mathcal{V}}^{(\dagger)}$ and $\hat{\mathcal{U}}^{(\dagger)}$, which are expressed as functions of the *truncated* dipole operator:

$$\hat{\mathcal{V}} = \exp\left[-\frac{i}{\hbar}\int d^3r\hat{\boldsymbol{\mathcal{P}}}_{\perp}(\mathbf{r})\cdot\mathbf{A}_{\perp}(\mathbf{r})\right] \tag{51}$$

$$\hat{\mathcal{U}} = \exp\left[\frac{i}{\hbar}\int d^3r\left(\hat{\boldsymbol{\mathcal{P}}}_{\perp}^{\rm d}(\mathbf{r}) - \hat{\boldsymbol{\mathcal{P}}}_{\perp}(\mathbf{r})\right)\cdot\mathbf{A}_{\perp}(\mathbf{r})\right], \tag{52}$$

where $\hat{\boldsymbol{\mathcal{P}}}_{\perp}$ is the arbitrary-gauge transverse polarization expressed in terms of the truncated dipole operator; note these functions can also be expressed in terms of the arbitrary-gauge vector potential through its longitudinal field component within the quantization scheme we employ here [4].

In this work we will focus on the two most common gauges in quantum optics[1]: the dipole (d) gauge and Coulomb (C) gauge. Within the quantization function scheme of Ref. [4], the transverse polarization corresponding to the Coulomb gauge is $\hat{\boldsymbol{\mathcal{P}}}_{\perp}^{\rm C}(\mathbf{r}) = 0$, and the dipole gauge $\hat{\boldsymbol{\mathcal{P}}}_{\perp}^{\rm d} = \hat{\mathbf{d}}\cdot\boldsymbol{\delta}^{\perp}(\mathbf{r})$ (that is, the multipolar gauge polarization within the dipole approximation). Considering these two gauges, we find that $\hat{\mathcal{U}}_{\rm d} = \hat{\mathcal{V}}_{\rm C} = 1$, and $\hat{\mathcal{U}}_{\rm C} = \hat{\mathcal{V}}_{\rm d}^{\dagger}$. Thus, we can define $\hat{\mathcal{W}} \equiv \hat{\mathcal{U}}_{\rm C}$, and write

$$\hat{\mathcal{H}}_{\rm C} = \hat{H}_{\rm em} + \hat{\mathcal{W}}\hat{\mathcal{H}}_0\hat{\mathcal{W}}^{\dagger} + \hat{\mathcal{H}}_{\parallel} \tag{53}$$

for the Coulomb gauge Hamiltonian, and

$$\hat{\mathcal{H}}_{\rm d} = \hat{\mathcal{W}}^{\dagger}\hat{H}_{\rm em}\hat{\mathcal{W}} + \hat{\mathcal{H}}_0 + \hat{\mathcal{H}}_{\parallel} \tag{54}$$

for the dipole gauge. Explicitly, we have

$$\hat{\mathcal{W}} = \exp\left[\frac{i}{\hbar}\mathbf{d}\cdot\hat{\mathbf{A}}_{\perp}(\mathbf{0})\hat{\sigma}_x\right], \tag{55}$$

and we see explicitly the advantage of having defined our quantized QNMs in terms of the vector potential; the operator used to transform between gauges $\hat{\mathcal{W}}$ (which preserves the correct gauge-invariant results) is naturally expressed in terms of purely quantized QNM operators. We also show a definition in terms of the transverse electric field operators in Sec. IV, which is what has been used in previous work, but is much less convenient when transforming between gauges, as one must take extra care in calculating the transformation $\hat{\mathcal{W}}$, which becomes expressed in this case in terms of both quantized QNM operators and residual reservoir operators. In particular, one must be careful to include the non-local commutators of the reservoir operators to obtain the correct transformation.

E. System-reservoir Hamiltonian

We now discuss how to split up the entire Coulomb and dipole gauge Hamiltonians into system (QNM), reservoir, and system-reservoir parts, in preparation for the derivation of an ME. By calculating the unitary transformation in Eq. (53), we find the general form of the full Coulomb gauge Hamiltonian,

$$\hat{\mathcal{H}}_{\rm C} = \hat{H}_{\rm em} + \frac{\hbar\omega_0}{2}\left[\cos(\hat{\Phi})\hat{\sigma}_z + \sin(\hat{\Phi})\hat{\sigma}_y\right] + \hat{\mathcal{H}}_{\parallel}, \tag{56}$$

where

$$\hat{\Phi} = \frac{2}{\hbar}\mathbf{d}\cdot\hat{\mathbf{A}}_{\rm QNM}^{\perp}(\mathbf{0}) = 2\sum_{\mu}\left[\eta_{\mu}\hat{a}_{\mu} + \eta_{\mu}^{*}\hat{a}_{\mu}^{\dagger}\right], \tag{57}$$

with the complex coupling constants

$$\eta_{\mu} = \frac{\mathbf{d}\cdot\ \tilde{\mathbf{f}}_{\mu}^{\rm s}(\mathbf{0})}{\sqrt{2\epsilon_0\hbar\chi_{\mu\mu}}}. \tag{58}$$

---

[1] Similar quantization schemes can be performed for superconducting QED systems, e.g., using the flux gauge and the charge gauge [35].

For the system component, we have, straightforwardly,

$$\hat{\mathcal{H}}_{\rm C}^{\rm S} = \hat{H}_{\rm QNM}^{\rm em} + \frac{\hbar\omega_0}{2}\Big[\cos{(\hat{\Phi})}\hat{\sigma}_z + \sin{(\hat{\Phi})}\hat{\sigma}_y\Big] + \hat{\mathcal{H}}_{\parallel}^{\rm S}, \tag{59}$$

and the longitudinal term is

$$\hat{\mathcal{H}}_{\parallel}^{\rm S} = -i\sum_{\mu}\hbar\chi_{\mu\mu}\left(\eta_{\mu}^{\parallel}\hat{a}_{\mu} - \eta_{\mu}^{\parallel *}\hat{a}_{\mu}^{\dagger}\right)\hat{\sigma}_x, \tag{60}$$

where $\eta_{\mu}^{\parallel} = \mathbf{d}\cdot\ \tilde{\mathbf{f}}_{\mu}^{\parallel}(\mathbf{0})/\sqrt{2\epsilon_0\hbar\chi_{\mu\mu}}$.

The system-reservoir coupling Hamiltonian is

$$\hat{\mathcal{H}}_{\rm C}^{\rm SR} = \hat{H}_{\rm QNM-R}^{\rm em} + \hat{B}_{\parallel}\hat{\sigma}_x, \tag{61}$$

where $\hat{B}_{\parallel} = -\mathbf{d}\cdot\hat{\mathbf{E}}_{\rm F,B}^{\parallel}(\mathbf{0})$. The total Coulomb gauge Hamiltonian is then $\hat{\mathcal{H}}_{\rm C} = \hat{\mathcal{H}}_{\rm C}^{\rm S} + \hat{\mathcal{H}}_{\rm C}^{\rm SR} + \hat{H}_{\rm R}^{\rm em}$.

In the dipole gauge, we have, performing a similar separation of $\hat{\mathcal{H}}_{\rm d} = \hat{\mathcal{H}}_{\rm d}^{\rm S} + \hat{\mathcal{H}}_{\rm d}^{\rm SR} + \hat{H}_{\rm R}^{\rm em}$,

$$\hat{\mathcal{H}}_{\rm d}^{\rm S} = \hat{\mathcal{H}}_{\rm QNM}^{\prime\rm em} + \frac{\hbar\omega_0}{2}\hat{\sigma}_z + \hat{\mathcal{H}}_{\parallel}^{\rm S}, \tag{62a}$$

$$\hat{\mathcal{H}}_{\rm d}^{\rm SR} = \hat{\mathcal{H}}_{\rm QNM-R}^{\prime\rm em} + \hat{B}_{\parallel}\hat{\sigma}_x, \tag{62b}$$

where we use primes to indicate that $\hat{\mathcal{H}}_{\rm QNM}^{\prime\rm em}$ and $\hat{\mathcal{H}}_{\rm QNM-R}^{\prime\rm em}$ are expressed in terms of $\hat{a}_{\mu}^{\prime}$ and $\hat{a}_{\mu}^{\dagger\prime}$, where $\hat{a}_{\mu} = \hat{W}_{\rm QNM}^{\dagger}\hat{a}_{\mu}\hat{W}_{\rm QNM} = \hat{a}_{\mu} + i\eta_{\mu}^{*}\hat{\sigma}_x$.

Dropping a term proportional to the TLS subspace identity operator, one can put the dipole gauge system Hamiltonian in the form of the canonical multimode quantum Rabi model:

$$\hat{\mathcal{H}}_{\rm d}^{\rm S} = \hat{H}_{\rm QNM} + \frac{\hbar\omega_0}{2}\hat{\sigma}_z + \sum_{\mu}\hbar\left[g_{\mu}^{\rm d}\hat{a}_{\mu} + g_{\mu}^{\rm d*}\hat{a}_{\mu}^{\dagger}\right]\hat{\sigma}_x, \tag{63}$$

where the dipole gauge QNM coupling constant is $g_{\mu}^{\rm d} = -i\sum_{\eta}\chi_{\eta\mu}\left(\eta_{\eta} + \eta_{\eta}^{\parallel}\right)$, which is generally complex.

We note that these Hamiltonians easily recover previously known results; in the single-mode limit, where $\chi_{cc} \approx \omega_{\rm c}$, and in the non-USC regime where we can expand η_{μ} to leading order (and neglecting $\eta_c^{\parallel}$), both gauges have the same system-reservoir and reservoir Hamiltonians, and the system Hamiltonians are $\hat{\mathcal{H}}_{\rm C}^{\rm C/d} \approx \omega_c a_c^{\dagger}a_c + \frac{\hbar\omega_0}{2}\hat{\sigma}_z + \hat{\mathcal{H}}_{\rm int}^{\rm C/d}$, where $\hat{\mathcal{H}}_{\rm int}^{\rm C/d} = \hbar\left[g_c^{\rm C/d}\hat{a}_c + g_c^{\rm C/d*}\hat{a}_c^{\dagger}\right]\hat{\sigma}_x$, and $g_c^{\rm C} = \eta_c\omega_0$, such that the ratio of Coulomb gauge to dipole gauge cavity coupling strengths is $|g_c^{\rm C}/g_c^{\rm d}| = \omega_0/\omega_c$, recovering well-known results (e.g., see Refs. 38 and 39).

F. Quasinormal mode master equation in the ultrastrong coupling regime

In the previous subsection, we derived the light-matter interaction Hamiltonian valid for arbitrary coupling regimes in the Coulomb as well as dipole gauge. In the following, we will use this theory to setup a QNM ME. We first derive these results in the spatially unspecified representation, and then show how to transform the resultant ME to the spatially specified representation, as required to obtain the correct QNM phase-dependent decay rates of the lossy dipole-cavity system [28].

1. *Dressed-state basis*

To start, we first introduce the dressed-state basis $\{|j\rangle\}$ in which the TLS-QNM system Hamiltonian $\hat{\mathcal{H}}_{\rm C}^{\rm S}$ takes on the diagonal form,

$$\hat{\mathcal{H}}_{\rm C}^{\rm S} = \hbar\sum_{j}\omega_j|j\rangle\langle j|, \tag{64}$$

where $\hbar\omega_j$ are the associated eigenenergies of the dressed states, and we write the system-reservoir Hamiltonian as

$$\hat{\mathcal{H}}_{\rm C}^{\rm SR} = \hat{B}_{\parallel}\hat{S}_{\parallel} + \left[\sum_{\mu}\hat{C}_{\mu}^{\dagger}\hat{a}_{\mu} + {\rm H.c.}\right], \tag{65}$$

where we have defined $\hat{S}_{\parallel} = \hat{\sigma}_x$, and $\hat{C}_\mu = \hbar \int_0^\infty d\omega \int d^3 r \mathbf{g}_\mu(\mathbf{r},\omega) \cdot \mathbf{c}(\mathbf{r},\omega)$. We work in the Coulomb gauge for this derivation, but the final ME we derive has a gauge-invariant form. The first term of Eq. (65) can be associated with the TLS coupling to background longitudinal modes, respectively (and we reiterate we have neglected background transverse couplings), and the term in square brackets gives the coupling of the QNM with the reservoir. Going forward from here, we will neglect the longitudinal contribution for simplicity, assuming the transverse QNM response to dominate, which is appropriate for realistic separations of the dipole to the plasmonic resonator. However, this term could be retained if desired, and the potential effect on the decay rates through $\mathbf{G}^{\parallel}(\mathbf{r_0},\mathbf{r},\omega)$ can be calculated using the same method as those we derive below.

Next, to facilitate a derivation in the USC regime, where light and matter degrees of freedom strongly hybridize, we move into the interaction picture. Denoting the interaction picture with a time argument, we find

$$\hat{C}_\mu(t) = \hbar \int_0^\infty d\omega \int d^3 r \mathbf{g}_\mu(\mathbf{r},\omega) \cdot \mathbf{c}(\mathbf{r},\omega) e^{-i\omega t}, \tag{66}$$

where we have assumed local bosonic commutation relations for the reservoir operators $\hat{\mathbf{c}}(\mathbf{r},\omega)$ and $\hat{\mathbf{c}}^\dagger(\mathbf{r},\omega)$, as justified in Sec. V. Similarly, we can decompose the system operators by writing them in the dressed-state basis

$$\hat{a}_\mu(t) = \sum_\alpha c_\alpha^\mu \hat{\sigma}_\alpha e^{-i\omega_\alpha t} + \text{H.c.} \tag{67}$$

Here, we have introduced the abbreviated notation in which α is an index that refers to the pair of combined photon-matter eigenstate indices (j,k), such that the energy of the state k is greater than that of the state j, and $c_\alpha^\perp = \langle j| \hat{S}^\perp |k\rangle$, $c_\alpha^\mu = \langle j| \hat{a}_\mu |k\rangle$, $\hat{\sigma}_\alpha = |j\rangle \langle k|$, and $\omega_\alpha = \omega_k - \omega_j$. In short, α runs over the transitions of the dressed states. Note that we are able to neglect diagonal matrix elements in Eq. (67), as $\hat{a}_\mu$ is odd under parity transformations, and parity is a symmetry of the system Hamiltonian $\hat{\mathcal{H}}_{\rm C}^{\rm S}$.

At this stage, we make a rotating-wave approximation (RWA) for the reservoir coupling, by dropping all terms in $\hat{\mathcal{H}}_{\rm SR}(t)$ that oscillate at a sum of a reservoir frequency ω and transition frequency ω_α [41, 44, 45]. These terms are always very rapidly-oscillating, and can be expected to contribute contributions to the ME only beyond the 2nd-order perturbative validity of the Born-Markov approximation [46] (and we have verified numerically they do not notably modify the spectra in our simulations). Making the reservoir-coupling RWA, we obtain

$$\hat{\mathcal{H}}_{\rm C}^{\rm SR}(t) = \sum_\alpha \sum_\mu \hat{C}_\mu^\dagger(t) c_\alpha^\mu \hat{\sigma}_\alpha e^{-i\omega_\alpha t} + \text{H.c.} \tag{68}$$

2. *Second-order Born-Markov master equation*

To derive the desired QNM ME, we perform a standard second-order Born-Markov approximation [46]:

$$\frac{d}{dt}\rho_I(t) = -\frac{1}{\hbar^2} \int_0^\infty \text{tr}_{\rm R} \left[\hat{\mathcal{H}}_{\rm C}^{\rm SR}(t), [\hat{\mathcal{H}}_{\rm C}^{\rm SR}(t-\tau), \rho_I(t)\rho_{\rm R}] \right] \text{d}\tau, \tag{69}$$

where we have traced over the reservoir degrees of freedom, and used ρ_I to denote the reduced system interaction picture density matrix. We let $\rho_{\rm R}$ correspond to the reservoir density matrix, which we choose to be the vacuum state, although other choices are possible (e.g., thermal, coherent states for coherent driving [41], or a squeezed vacuum [47, 48]).

Under the assumption of a vacuum state reservoir (zero temperature), and bosonic commutator relations, we have the result that $\text{tr}_{\rm R}[\hat{\mathbf{c}}(\mathbf{r},\omega)\hat{\mathbf{c}}^\dagger(\mathbf{r}',\omega')] = \mathbf{I}\delta(\mathbf{r}-\mathbf{r}')\delta(\omega-\omega')$, and all other combinations of two reservoir operators vanish.

Under these assumptions, we move back to the Schrödinger picture and simplify Eq. (69) to find

$$\frac{d}{dt}\rho = -\frac{i}{\hbar}[\hat{\mathcal{H}}_{\rm S},\rho] + \sum_{\alpha\alpha} F_{\alpha'\alpha} \left[\hat{\sigma}_\alpha \rho \hat{\sigma}_{\alpha'}^\dagger - \hat{\sigma}_{\alpha'}^\dagger \hat{\sigma}_\alpha \rho \right] + \text{H.c.}, \tag{70}$$

where

$$F_{\alpha'\alpha}^{\rm QNM} = \frac{1}{\hbar^2} \sum_{\mu\eta} c_{\alpha'}^{\mu *} c_\alpha^\eta \int_0^\infty d\tau \langle \hat{C}_\mu \hat{C}_\eta^\dagger(-\tau) \rangle e^{i\omega_\alpha \tau}. \tag{71}$$

At this stage, one could make a secular approximation by dropping all terms where $\alpha \neq \alpha'$. The justification for this is that, upon moving back to the interaction picture, the second line of Eq. (70) would pick up an oscillatory factor of $e^{-i(\omega_\alpha - \omega_{\alpha'})t}$. If $\omega_\alpha - \omega_{\alpha'}$ were much greater than the greatest values of $F_{\alpha\alpha'}$, these terms would oscillate fast in the interaction picture and average out to provide no contribution to the system dynamics. In this case, the secular

criterion is typically satisfied for strong coupling of the dipole-cavity system (coupling strength exceeding the decay rates), such that the anharmonic quantum Rabi model spectrum gives well-separated emission lines for all driven transitions [49]. Moving forward, we will assume strong coupling, and thus make the secular approximation for the sake of simplifying the ME presentation. In our numerical results of the main text, we keep the full non-secular ME for potentially more precise results.

By integrating over τ in Eq. (71), we find $F_{\alpha\alpha} = \frac{1}{2}\Gamma_\alpha^{\rm R} + i\delta_\alpha^{\rm V}$, where $\Gamma_\alpha^{\rm R}$ and $\delta_\alpha^{\rm V}$ are real parameters, and we can write the QNM ME as

$$\dot{\rho} = -\frac{i}{\hbar}[\hat{\mathcal{H}}_{\rm S}, \rho] + \sum_\alpha \frac{\Gamma_\alpha}{2}\mathcal{L}[\hat{\sigma}_\alpha]\rho, \tag{72}$$

where Γ_α is the decay rate corresponding to loss of polariton excitations from the dipole-cavity system for the transition between eigenstates of the system Hamiltonian indexed by α. In general, there should also be a Hamiltonian term $\hat{\mathcal{H}}_{\rm V} = \sum_\alpha \hbar\delta_\alpha^{\rm V}\hat{\sigma}_\alpha^\dagger\hat{\sigma}_\alpha$ present in Eq. (72). This is the (reservoir-enabled) Lamb shift Hamiltonian, which is responsible for small reservoir-induced shifts of the system energy levels due to virtual polariton-polariton exchange with the reservoir. We shall neglect these shifts here and focus solely on the dissipator terms, as is the common approach in cavity-QED.

The total decay rate in the spatially unspecified representation for transition indexed by α is given by Γ_α, where

$$\Gamma_\alpha = 2\pi\sum_{\mu\eta} c_\alpha^{\mu *} c_\alpha^\eta g_{\mu\eta}(\omega_\alpha), \tag{73}$$

where

$$g_{\mu\eta}(\omega) = \int d^3r\,\mathbf{g}_\mu(\mathbf{r},\omega)\cdot\mathbf{g}_\eta^*(\mathbf{r},\omega) = \frac{2}{\pi}\sum_{\nu\nu'}\left[S^{-\frac{1}{2}}\right]_{\mu\nu}\left[S^{-\frac{1}{2}}\right]_{\nu'\eta}\frac{\sqrt{\omega_\nu\omega_{\nu'}}}{\omega^2}B_\nu(\omega)B_{\nu'}^*(\omega)\left[S_{\nu\nu'}^{\rm nrad}(\omega) + S_{\nu\nu'}^{\rm rad}(\omega)\right]. \tag{74}$$

As we will discuss in the following subsection, the decay rates in Eq. (73) are in the spatially unspecified representation, and do not accurately reflect frequency-dependent corrections beyond phenomenological models in the single-mode approximation, due to their resulting independence of the QNM phase at the dipole location.

Note that although we have derived the QNM ME [Eq. (72)] in the Coulomb gauge, as it is expressed entirely in terms of operators of the dressed-state basis of the system, it is clearly gauge-invariant. To calculate the decay rates Γ_α, the matrix elements $c_\alpha^\mu = \langle j|\,\hat{a}_\mu\,|k\rangle$ were defined using their Coulomb gauge representations, but one could as easily calculate these using eigenstates calculated from the dipole gauge representation by instead considering matrix elements of the operators $\hat{W}^\dagger\hat{a}_\mu\hat{W} = \hat{a}'_\mu = \hat{a}_\mu + i\eta_\mu^*\hat{\sigma}_x$. This *manifest* gauge invariance is a consequence of our formulation of the quantized QNMs in terms of the transverse vector potential (and neglect of background terms), which leads to an entirely system-level gauge transformation. Nonetheless, even with a gauge transformation which involves the reservoir, we have shown recently that one can expect gauge invariance of the Born-Markov ME to be retained generally wherever the Markov approximation holds [50]. To be clear, the eigenstates, photon-matter entanglement and other state properties are gauge-dependent (especially in the USC regime), but eigenenergies and physical observables obtained from either ME are not.

3. *Single-mode limit and spatially specified representation*

We now consider the single-mode limit of the quantized QNM ME derived in the previous subsection. To check the accuracy of our approach, which calculates frequency-dependent cavity decay rates (i.e., using an ab initio formulation of the spectral density), we compare with the result we recently derived for the spectral density of a lossy quantized cavity where a single dipole is weakly coupled to a single cavity mode with index $\mu = c$ [28] (within the secular approximation considered here):

$$\Lambda_c(\omega_\alpha) = \sqrt{\frac{\kappa_c}{2\pi}\frac{\omega_c}{\omega_\alpha}\zeta_c(\phi_0,\omega_\alpha)}\,, \tag{75}$$

where κ_c is the empty-cavity photon decay rate, and $\phi_0 = \arg\{\mathbf{n_d}\cdot\tilde{\mathbf{f}}_c(\mathbf{0})\}$ is the QNM phase at the dipole location projected onto the dipole moment unit vector $\mathbf{n_d}$. Crucially, frequency-dependent corrections to system decay rates beyond those of phenomenological (e.g., flat spectral density) models of lossy quantized cavities are highly dependent on the QNM phase at the dipole location for realistic cavities [28].

However, the decay rates calculated in Eq. (73) *do not*, in the spatially unspecified form written, depend directly on the QNM phase at the dipole location. As such, when making a single (or few) mode approximation, they are not fully accurate in accounting for corrections beyond phenomenological lossy cavity models. Quasi-static plasmon-based

approaches which lead similarly to phase-independent frequency-dependent decay rates [51, 52] also fail to capture the accurate frequency dependence for the dominant transverse QNMs studied here. We stress that the spatially unspecified representation has previously been used to successfully explain many important quantum optical effects beyond phenomenological models, such as large Fano-like resonances, and coupled gain-loss systems, but in that case the dominant quantized QNM effects were captured from inter-QNM interference effects, beyond the single QNM approximation [15, 33, 53]. By moving to the spatially specified representation in the single QNM approximation, we can correctly identify additional phase effects that were less important in previous works, where the dominant effects were captured by the quantization approach in the spatially unspecified representation, and USC, and more generally, the broadband dissipative regime, was not considered.

To fix this situation, we move to the spatially specified form discussed in Sec. I C 3 in the context of the quantization parameter S_c. By employing Eq. (40) in the single-mode limit, letting $\mathbf{r}_1 = \mathbf{r}_2 = \mathbf{0}$, and taking the dot product on the right and left with $\mathbf{n_d}$, we obtain the result from Eq. (41), with ϕ_0.

Next, we note that the QNM decay rate Γ_α in the single-mode approximation can be written as

$$\begin{aligned}\Gamma_\alpha &= 4\omega_c|c_\alpha^c|^2\frac{(\omega_\alpha-\omega_c)^2+\kappa_c^2/4}{S_c\omega_\alpha^2}\int d^3r\epsilon_I(\mathbf{r},\omega_\alpha)|A_c\tilde{\mathbf{F}}'_c(\mathbf{r},\omega_\alpha)|^2\\ &= 4\omega_c|c_\alpha^c|^2\frac{(\omega_\alpha-\omega_c)^2+\kappa_c^2/4}{S_c\omega_\alpha^2}\mathrm{Im}\{A_c(\omega_\alpha)e^{i2\phi_0}\}\\ &= |c_\alpha^c|^2\frac{\kappa_c\cos{(2\phi_0)}\omega_c}{S_c\omega_\alpha}\zeta_c(\phi_0,\omega_\alpha),\end{aligned} \tag{76}$$

where in the second line we have used Eq. (41), and $\zeta_c(\phi_0, \omega_\alpha)$ was defined in Eq. (43)

Clearly, this form of the quantized cavity decay rate as a function of the hybridized TLS-cavity resonances ω_α, now depends explicitly on the QNM phase at the dipole location ϕ_0, which is known to have a significant impact for realistic cavities. By identifying the empty-cavity decay rate as κ_c (consistent with previous work on QNMs [7, 15]), and noting that, within the dipole spatial location representation, $S_c \approx \cos 2\phi_0$, we see that the empty-cavity result $\Gamma = \kappa_c$ is recovered. In summary, we have

$$\Gamma_\alpha = \kappa_c|c_\alpha^c|^2\frac{\omega_c}{\omega_\alpha}\zeta_c(\phi_0,\omega_\alpha). \tag{77}$$

Finally, identifying $\Gamma_\alpha = 2\pi|c_\alpha^c|^2\Lambda_c^2(\omega_\alpha)$, we see that the *ab initio* quantized QNM theory within the spatially specified representation fully recovers the correct form of the spectral density [Eq. (75)] needed to see frequency-dependent corrections beyond phenomenological models. We also see explicitly that for a model of cavity-reservoir coupling where the cavity operators $\hat{a}_c$, $\hat{a}_c^\dagger$ couple directly to the reservoir *in the Coulomb gauge*, the correct spectral density for this coupling has an overall $1/\omega$ scaling, confirming our findings in Ref. [28] from an *ab initio* construction.

G. Output observables and near-field detection

To relate the dynamics of the ultrastrongly-coupled cavity-QED system to observables which can be probed in photodetection experiments (that is, normally-ordered correlation functions of the EM fields), we will assume near-field detection, where the field can be described by the QNM expansion of the field. The more common scenario of far-field detection requires an input-output model, of which formulating a self-consistent theory in the USC regime using QNMs is a subtle and more involved process than the usual formulation (and can involve further gauge considerations [37, 50]), and so this problem will be addressed in future work.

In Ref. [4], we showed that the correct operator to use to model photodetection near the resonator (where the field can be well represented as a sum of QNM functions) is $-\mathbf{d}_{\rm det}\cdot\hat{\boldsymbol{\mathcal{E}}}_\perp(\mathbf{r}_{\rm det})$, where $\mathbf{d}_{\rm det}$ is an effective dipole moment of the detector system located at $\mathbf{r}_{\rm det}$, and, in the Coulomb gauge,

$$\hat{\boldsymbol{\mathcal{E}}}_\perp(\mathbf{r}_{\rm det}) = i\sum_\mu\sqrt{\frac{\hbar\omega_\mu}{2\epsilon_0}}\tilde{\mathbf{f}}_\mu^{\rm s(\mathbf{E})}(\mathbf{r}_{\rm det})\hat{a}_\mu + \text{H.a.}, \tag{78}$$

where

$$\tilde{\mathbf{f}}_\mu^{\rm s(\mathbf{E})}(\mathbf{r}_{\rm det}) = \sum_\nu\frac{\chi_{\nu\mu}}{\sqrt{\omega_\mu\omega_\nu}}\tilde{\mathbf{f}}_\nu^{\rm s}(\mathbf{r}_{\rm det}), \tag{79}$$

and $\hat{\boldsymbol{\mathcal{E}}}_\perp$ is the "correct" (in terms of photodetection) form of the part of the transverse electric field that can be expressed in terms of QNM operators, when the QNM operators are defined with respect to a modal expansion in terms of the transverse vector potential, as we show in Ref. [4]. Note in Ref. [4], Eq. (78) is referred to as the

"correctly-truncated form of the electric field" in the sense of a mode truncation of the field expansion. In this case, it is more correct to say this is the correct form of the electric field operator when decomposing the total field into QNM and reservoir subspaces, but the procedure and result for the QNM (system) part is the same. As an aside, and in contrast, it can be shown that directly substituting the projection functions $\mathbf{L}_\mu(\mathbf{r},\omega)$ into the definition of $\hat{\mathbf{E}}_{\rm F}^{\perp}$ gives $\hat{\mathbf{E}}_{\rm F,QNM}^{\perp}(\mathbf{0}) = -i\sum_\mu \hbar g_\mu^{\rm d}\hat{a}_\mu + {\rm H.c.}$, where $g_\mu^{\rm d}$ is defined in Sec. I F. Under the approximation $\chi_{\mu\mu} \approx \omega_\mu$, we recover $\hat{\mathbf{E}}_{\rm F,QNM}^{\perp}(\mathbf{0}) = \hat{\boldsymbol{\mathcal{E}}}_\perp(\mathbf{0})$.

In the dipole gauge, the only difference is that $\hat{\boldsymbol{\mathcal{E}}}_\perp$ should be expanded in terms of the $\hat{a}'_\mu$ and $\hat{a}'^\dagger_\mu$ operators. Note that this procedure is consistent with previous calculations using phenomenological loss models [41, 49].

In the single-mode approximation, we have

$$\tilde{\mathbf{f}}_c^{\rm s(\mathbf{E})}(\mathbf{r}_{\rm det}) = \frac{\chi_c S_c}{\omega_c}\tilde{\mathbf{f}}_c(\mathbf{r}_{\rm det}), \tag{80}$$

or, using the spatially specified representation and approximating $\chi_c \approx \omega_c$,

$$\tilde{\mathbf{f}}_c^{\rm s(\mathbf{E})}(\mathbf{r}_{\rm det}) \approx \sqrt{\cos{(2\phi_0)}}\tilde{\mathbf{f}}_c(\mathbf{r}_{\rm det}). \tag{81}$$

In either case, the detected observables can be calculated by using the $\hat{a}_c$ and $\hat{a}_c^\dagger$ operators (or with primes if using the dipole gauge), up to a proportionality constant. In the USC regime, we further must move to the dressed state basis to ensure observables correspond to real (and not virtual) excitations [44], which is done in the main text.

II. ADDITIONAL RESONATOR EXAMPLES

In this section, we show three more examples of resonator structures from which we can identify dominant QNMs and compare the weak-coupling dipole decay rate calculated under a QNM expansion with full Maxwell simulations. We first consider a cylindrical gold dimer setup similar to the one studied in Ref. [28], but with slightly different dimensions, where the dielectric function of the gold nanoparticle is also governed by the Drude model given in the main text; this is shown in Fig. S2. The bare cavity mode has a radiative photon loss fraction of (using the spatially unspecified representation of the quantized QNMs) $S_c^{\rm rad}/S_c \approx 68\%$, with $S_c \approx 1$.

We then show a 3D PC beam cavity example in Fig. S3, also similar to a cavity shown in Ref. [8, 24, 28, 54]. The finite length of the PC beam cavity is 6.052 μm, and the height/width of the beam are $h_{\rm PC} = 200$ nm and $W_{\rm PC} = 376$ nm. In the taper region, there are 7 air holes (a_1, a_2, a_3, a_4, a_5, a_6, a_7), where the radius/center-to-center distance are increasing linearly from 68/264 nm to 86/299 nm. In addition, 3 identical air holes with a radius of 86 nm and center-to-center spacing of 306 nm are added in the mirror region. The surface-to-surface distance between two middle holes (two a_1) is $s_{\rm cavity} = 126$ nm. The potential $z-$polarized dipole is placed at $d = 5$ nm above the cavity center. The dielectric function $\epsilon_{\rm PC}(\omega)$ of the PC beam is also modeled by a single Lorentz oscillator model, $\epsilon_{\rm PC}(\omega) = \epsilon_\infty - \frac{(\epsilon_{\rm s}-\epsilon_\infty)\omega_{\rm t}^2}{\omega^2-\omega_{\rm t}^2+i\omega\gamma_{\rm L}}$, with $\epsilon_{\rm s} = 2.04^2 = 4.1616$, $\omega_{\rm t} = 12$ eV and $\gamma_{\rm L} = 0.0131$ eV.

For both examples, the parameters associated with the dominant QNMs are shown in Table S1. In both cases, our observations are similar to the ones made in both the main text and Ref. [28]. The plasmonic dimer example, with its large $|\eta_c^{(1)}|$ should be compatible with our *ab initio* quantized single-mode QNM theory in the USC regime, and its broadband regime coincides with USC for resonant coupling. The PC cavity, in contrast, we predict requires a multimode model to accurately predict spectral quantities in the USC regime. Moreover, as $\tilde{\Omega}_{\rm BB}Q_c \approx 50.2$, but deviations from the single-QNM theory occur beyond ~ 10 linewidths from the resonance, single-mode models are also insufficient to calculate accurate observables beyond phenomenological approaches for this dominant QNM.

As a final additional example, we also consider a 2D lossy microdisk with a diameter of 10 μm, where the constant refractive index of the disk is $n_{\rm disk} = 2.0 + 10^{-5}i$. We find a dominant whispering-gallery mode (WGM), and we plot the mode and phase distribution of this dominant QNM in Fig. S4 (a-c). We consider a dipole (line current) location 10 nm away from the disk. Here a non-monotonic frequency dependence is observed [when normalized to $L_c(\omega_0)$], and values of $|\eta^{(1)}| = 0.088$ and $\tilde{\Omega}_{\rm BB} = 0.0042$ again suggest limitations to single-mode dissipative models of USC and broadband dynamics for this mode. Here, in fact, these limitations are *extremely* significant; with a characteristic scaled dynamical rate $\tilde{\Omega}_{\rm BB}$, the bandwidth of the system dynamics spans a number of bare cavity linewidths κ_c equal to $\sim \tilde{\Omega}_{\rm BB}Q_c \approx 425$, but from Fig. S4 (e), our *ab initio* QNM correction to the dissipative dynamics remains accurate only around a single linewidth of the cavity mode. Such a result can be likely be attributed to the quasi-1D nature of such ring resonators, leading to a quasi-harmonic distribution of longitudinal modes, and a strong reduction of the potential for broadband dynamics to be constrained to a single-mode subspace. We expect this feature to be a general property of 1D and quasi-1D cavity designs, which also highlights the importance of our *ab initio* approach which is appropriate for full 3D cavity geometries. The emission here is almost entirely nonradiative, with $S_c^{\rm nrad}/S_c \approx 1$;

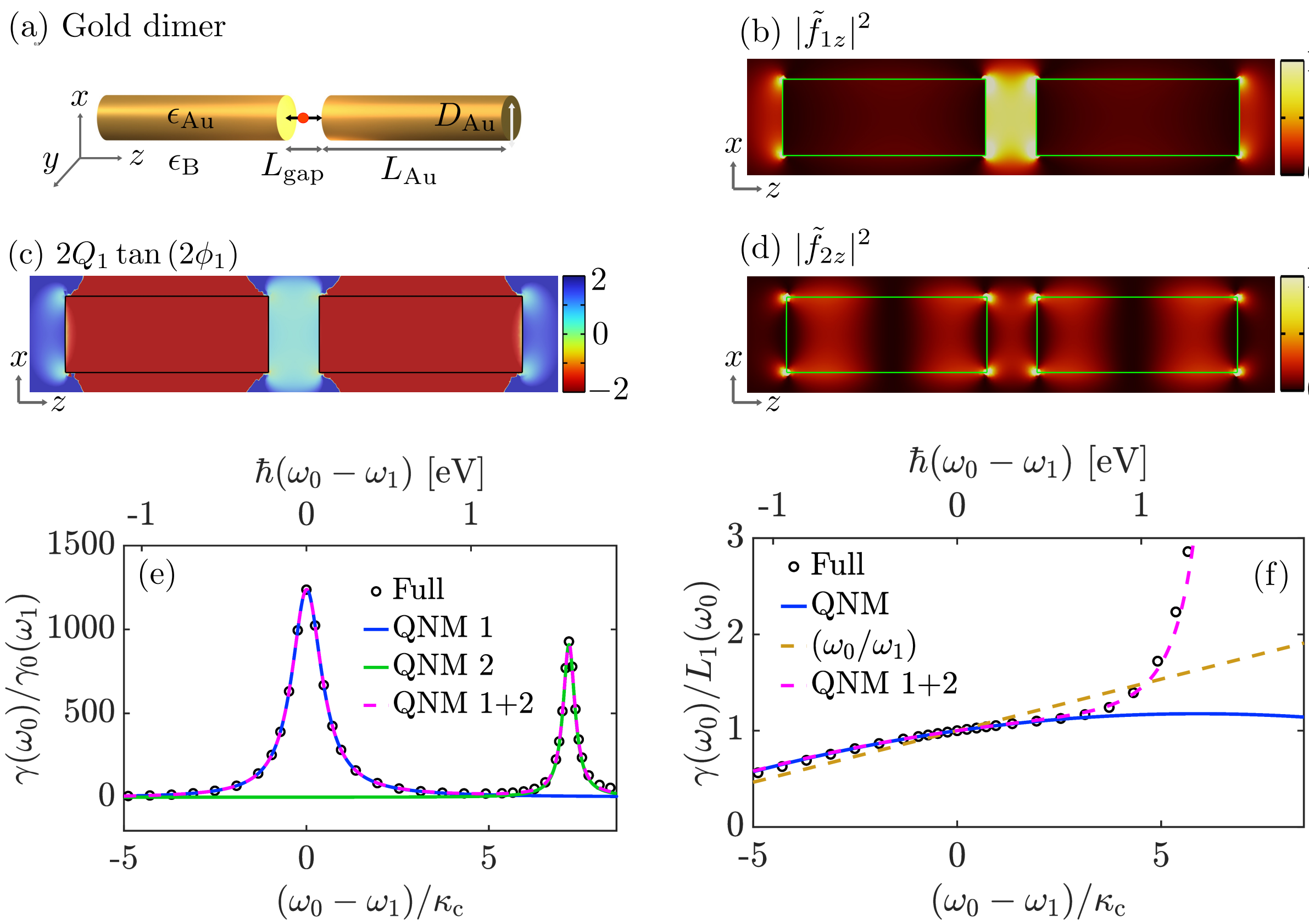

Figure S2. (a) Schematic of gold cylinder dimer in free space ($\epsilon_{\rm B} = 1.0$) with dimensions $D_{\rm Au} = 30$ nm, $L_{\rm Au} = 80$ nm, and $L_{\rm gap} = 20$ nm. QNM profile (b) dominant QNM (1) and (d) second QNM (2) (arb. units). (c) Phase distribution of $2Q_1 \tan(2\phi_1)$ for dominant QNM, where the phase is defined from $\tilde{f}_{1z}(\mathbf{r}) = |\tilde{f}_{1z}(\mathbf{r})|e^{i\phi_1(\mathbf{r})}$. (e) Purcell factors for a $z-$polarized emitter at the gap center, normalized by the free-space rate $\gamma_0(\omega) = |\mathbf{d}|^2\omega^3/(3\pi\epsilon_0\hbar c^3)$. (f) The decay rate normalized by Lorentzian function $L_c(\omega_0)$. Here, we plot the full dipole decay rate with the background $\gamma_0(\omega_0)$ subtracted out. The dominant QNM (1) parameters are found in Table S1, and QNM 2 has (complex) eigenfrequency $\hbar\tilde{\omega}_2 = (3.665 - 0.0438i)$ eV and projected phase at the dipole location $\phi_2(\mathbf{r}_0) = 0.000612$.

	Cyl. Dimer	PC Beam	WGM
$\hbar\tilde{\omega}_c$ (eV)	$2.070 - 0.1108i$	$1.598 - 0.0003662i$	$0.8337 - 4.120 \times 10^{-6}i$
Q_c	9.34	2182	1.012×10^5
$\tilde{f}_z$	9.009×10^{10} $+1.066 \times 10^9 i$ m$^{-\frac{3}{2}}$	1.557×10^9 $+4.039 \times 10^6 i$ m$^{-\frac{3}{2}}$	86750 $-2.440i$ m^{-1}
$\tan(2\phi_0) \approx 2\phi_0$	0.0237	0.00519	-5.63×10^{-5}
$\lvert\eta_c^{(1)}\rvert$	2.3	0.044	0.088
$\tilde{\Omega}_{\rm BB}$	0.1	0.023	0.0042

Table S1. Parameters of dominant QNM for additional cavity structures.

however, this is really just a consequence of modeling the disk with a complex refractive index and having a 2D WGM. In more practical examples, the disk would be 3D and would thus yield vertical decay emission, and also such resonators are often coupled to an output waveguide mode, such as through an evanescently coupled fiber.

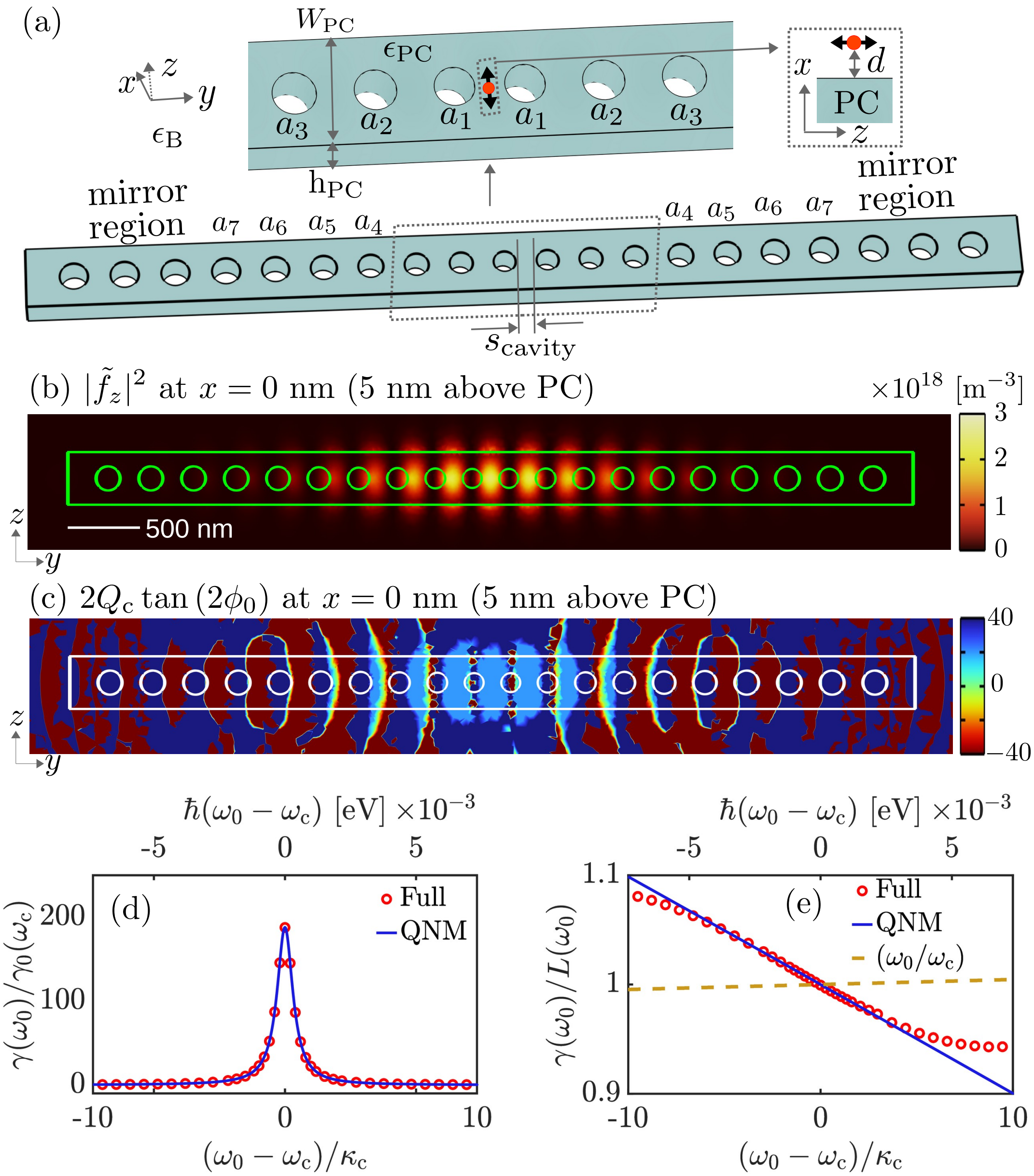

Figure S3. (a) Schematic of PC beam cavity in free space ($\epsilon_B = 1$). The permittivity ϵ_{PC} is governed by a single Lorentz oscillator model. (b) Mode profile of dominant QNM. (c) Phase distribution of $2Q_c \tan(2\phi_0)$. (d) Purcell factors for a $z-$polarized emitter 5 nm above the cavity center, normalized by the free-space rate $\gamma_0(\omega) = \mathbf{d}^2\omega^3/(3\pi\epsilon_0\hbar c^3)$. (e) Decay rate normalized by Lorentzian function $L_c(\omega_0)$. Here, we plot the full dipole decay rate with an approximate background $1.78\gamma_0(\omega_0)$ subtracted out.

III. VANISHING OF BACKGROUND TRANSVERSE VECTOR POTENTIAL OPERATOR

Here we show that when using the quantized QNM projection functions $\mathbf{L}_\mu(\mathbf{r},\omega)$, the background contribution to the transverse vector potential (the part expressed in terms of the residual reservoir operators $\hat{\mathbf{c}}$, $\hat{\mathbf{c}}^\dagger$), $\hat{\mathbf{A}}_{\rm R}^{\perp}(\mathbf{r})$, vanishes. To do so, we first make use of the following relation [55], valid for all μ:

$$\int d^3r \int d\omega \mathbf{L}_\mu(\mathbf{r},\omega)\cdot\hat{\mathbf{c}}(\mathbf{r},\omega) = 0$$
$$\sum_\nu \left[S^{-\frac{1}{2}}\right]_{\mu\nu} \hat{x}_\nu = 0, \tag{82}$$

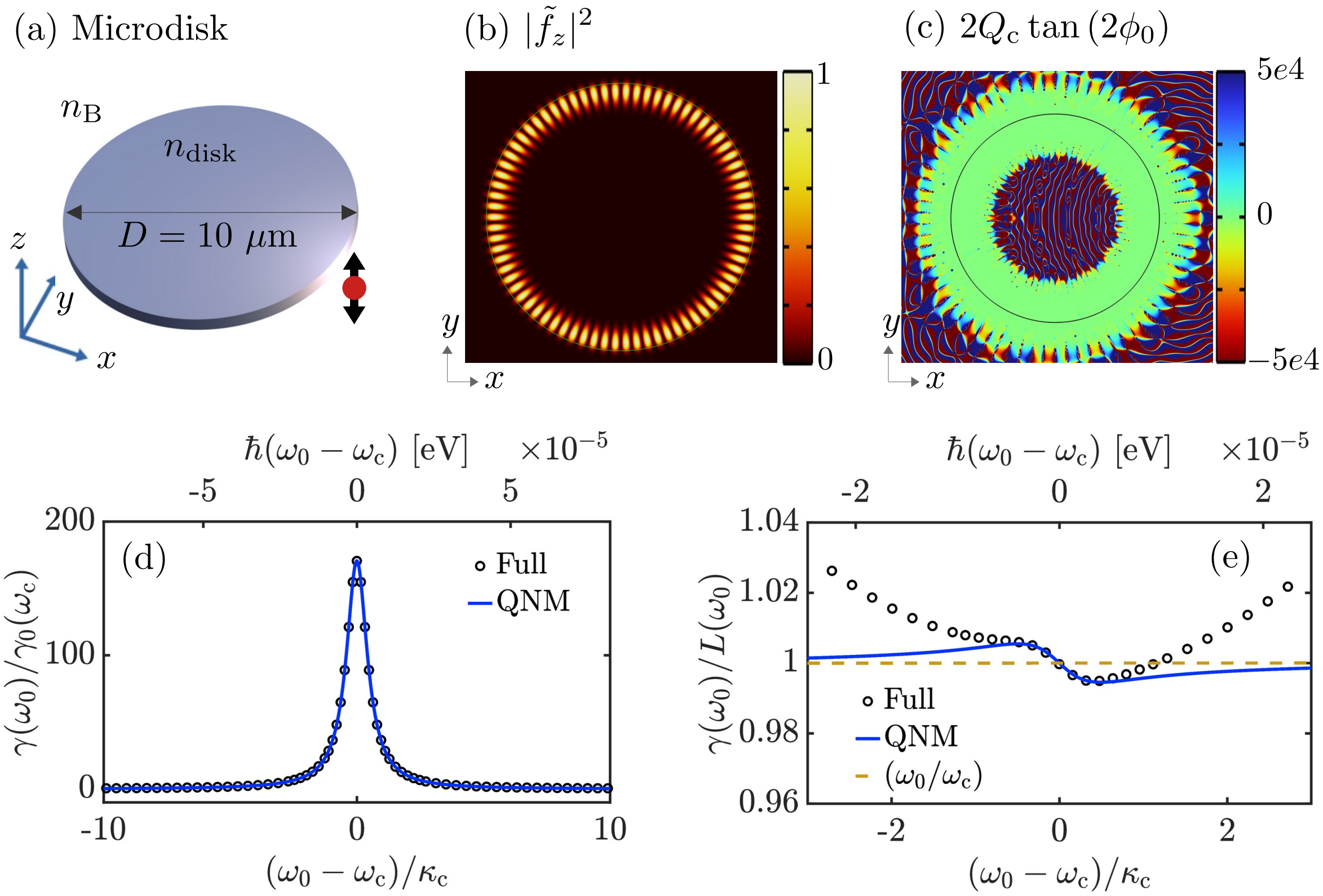

Figure S4. (a) 3D schematic of microdisk (however, note our simulation is in 2D for this structure). $n_{\rm disk} = 2.0+10^{-5}i$. $n_{\rm B} = 1$. (b) Mode profile for QNM $|\tilde{f}_z|^2$ (arb. units; TM mode—this is the only component). (c) Phase distribution of $2Q_{\rm c}\tan(2\phi_0)$ for dominant QNM, where the phase is defined from $\tilde{f}_z(\mathbf{r}) = |\tilde{f}_z(\mathbf{r})|e^{i\phi_0(\mathbf{r})}$. (d) Purcell factors for an emitter (line current) 10 nm away from the disk, normalized by the 2D free-space rate $\gamma_0^{\rm 2D}(\omega) = |\mathbf{d}^{\rm 2D}|^2\omega^2/(2\epsilon_0\hbar c^2)$. (e) Decay rate normalized by Lorentzian function $L(\omega_0) = \gamma_{\rm c}^{\rm QNM}(\omega_c) \times \frac{\kappa_{\rm c}^2/4}{\kappa_{\rm c}^2/4+(\omega_0-\omega_c)^2}$. Here, we plot the full dipole decay rate with an approximate background $0.5\gamma_0^{\rm 2D}(\omega_0)$ subtracted out. The dominant QNM parameters are found in Table S1.

where $\hat{x}_\nu = \sqrt{2\omega_\nu/\pi}\,\hat{y}_\nu$, and

$$\hat{y}_\nu = \int d^3r' \int \frac{d\omega}{\omega}\sqrt{\epsilon_I(\mathbf{r}',\omega)}A_\nu(\omega)\tilde{\mathbf{F}}'(\mathbf{r}',\omega)\cdot\hat{\mathbf{c}}(\mathbf{r}',\omega). \tag{83}$$

Now, S, and thus $S^{-\frac{1}{2}}$, are positive definite matrices [55], which implies $\sum_{\mu\nu} x_\mu^* \left[S^{-\frac{1}{2}}\right]_{\mu\nu} x_\nu > 0$ for any complex non-zero vector x_μ. We can thus consider *any* given matrix element x_ν^j (indexed by j) of $\hat{x}_\nu$, and find as a consequence of Eq. (82) that

$$\sum_{\mu\nu} x_\mu^{j*} \left[S^{-\frac{1}{2}}\right]_{\mu\nu} x_\nu^j = 0, \tag{84}$$

which, as it is true for any arbitrary matrix element, implies $\hat{x}_\mu = 0$, and thus $\hat{y}_\mu = 0$. Next, we note that $\hat{\mathbf{A}}_{\rm R}^\perp$ can be written as $\sqrt{\frac{\hbar}{\pi\epsilon_0}}\sum_\mu \tilde{\mathbf{f}}_\mu(\mathbf{r})\hat{y}_\mu$, which thus vanishes.

IV. DERIVATION OF HAMILTONIAN WITH QUANTIZED QUASINORMAL MODE OPERATORS DEFINED WITH RESPECT TO AN ELECTRIC FIELD EXPANSION

In the main text, we worked with a projection function for the quantum QNMs defined such that the vector potential can be expanded directly in a basis of quantized QNMs. Alternatively, and as has been done in previous work on quantized QNMs, one could define them such that the transverse electric field (more precisely, $\hat{\mathbf{E}}_{\rm F}^\perp$, the component of

the transverse field which can be expressed in terms of bosonic field + medium polariton operators) can be expanded directly. In this case, the projection operator is

$$\mathbf{L}_{\mu}^{(\mathbf{E})}(\mathbf{r},\omega)=\sum_{\nu}\left[S^{(\mathbf{E})}\right]_{\mu\nu}^{-\frac{1}{2}}\sqrt{\frac{2\epsilon_I(\mathbf{r},\omega)}{\pi\omega_\nu}}A_\nu(\omega)\tilde{\mathbf{F}}'_\nu(\mathbf{r},\omega), \tag{85}$$

which is equivalent to that in the expansion for the transverse vector potential (Eq. (26)) aside from an additional factor of ω/ω_ν. Additionally, the S quantization matrix is modified:

$$S_{\mu\nu}^{(\mathbf{E})}=\frac{2}{\pi\sqrt{\omega_\mu\omega_\nu}}\int d^3r\int d\omega\epsilon_I(\mathbf{r},\omega)A_\mu(\omega)A_\nu^*(\omega)\tilde{\mathbf{F}}'_\mu(\mathbf{r},\omega)\cdot\tilde{\mathbf{F}}'^*_\nu(\mathbf{r},\omega), \tag{86}$$

which is the same as $S_{\mu\nu}$ in Eq. (27) but with an additional factor of $\omega^2/(\omega_\mu\omega_\nu)$. This leads to an expansion of the transverse field $\hat{\mathbf{E}}_{\rm F}^{\perp}$ of (for $\mathbf{r}$ in the *system* region)

$$\hat{\mathbf{E}}_{\rm F}^{\perp}(\mathbf{r})=i\sum_{\mu}\sqrt{\frac{\hbar\chi_{\mu\mu}}{2\epsilon_0}}\tilde{\mathbf{f}}_{\mu}^{s(\mathbf{E})}(\mathbf{r})\hat{a}_\mu+\text{H.c.}, \tag{87}$$

where

$$\tilde{\mathbf{f}}^{s(\mathbf{E})}(\mathbf{r})=\sum_{\nu}\left[S^{(\mathbf{E})}\right]_{\nu\mu}^{\frac{1}{2}}\tilde{\mathbf{f}}_\nu(\mathbf{r})\sqrt{\frac{\omega_\nu}{\chi_{\mu\mu}}}. \tag{88}$$

Under this definition, the dipole gauge Hamiltonian becomes

$$\hat{\mathcal{H}}_{\rm d}^{\rm S}=\hat{\mathcal{H}}_{\rm QNM,(\mathbf{E})}^{\rm em}+\frac{\hbar\omega_0}{2}\hat{\sigma}_z+\hat{\mathcal{H}}_{\parallel}^{\rm S}, \tag{89}$$

$$\hat{\mathcal{H}}_{\rm d}^{\rm SR}=\hat{\mathcal{H}}_{\rm QNM-R,(\mathbf{E})}^{\rm em}+\hat{B}_{\parallel}\hat{\sigma}_x, \tag{90}$$

where in $\hat{\mathcal{H}}_{\rm QNM,(\mathbf{E})}^{\rm em}=\sum_{\mu\nu}\hbar\chi_{\mu\nu}\hat{a}_\mu^\dagger\hat{a}_\nu$ the χ matrix is now computed with $\mathbf{L}^{(\mathbf{E})}$, and $\hat{\mathcal{H}}_{\rm QNM-R,(\mathbf{E})}^{\rm em}=\sum_\mu\hat{C}_\mu^{(\mathbf{E})}\hat{a}_\mu^\dagger+$H.c., with

$$\hat{C}_\mu^{(\mathbf{E})}=\hbar\int d^3r\int d\omega\mathbf{g}_\mu^{(\mathbf{E})}(\mathbf{r},\omega)\cdot\hat{\mathbf{c}}(\mathbf{r},\omega), \tag{91}$$

where

$$\mathbf{g}_{\mu}^{(\mathbf{E})}(\mathbf{r},\omega)=\sum_{\nu}\left[S^{(\mathbf{E})}\right]_{\mu\nu}^{-\frac{1}{2}}\sqrt{\frac{2\epsilon_I(\mathbf{r},\omega)}{\pi\omega_\nu}}B_\nu(\omega)\tilde{\mathbf{F}}'_\nu(\mathbf{r},\omega), \tag{92}$$

which is the same as Eq. (38), but with an additional factor of ω/ω_ν. Following this change through to the calculation of the QNM decay rate, we find

$$\Gamma_\alpha^{(\mathbf{E})}=\kappa_c|c_\alpha^c|^2\frac{\omega_\alpha}{\omega_c}\zeta_c(\phi_0,\omega_\alpha), \tag{93}$$

which is the same as Γ_α in Eq. (77), but with an additional factor of $\omega_\alpha^2/\omega_c^2$. This gives a spectral density of $\Lambda_{(\mathbf{E})}^2(\omega_\alpha)=\kappa_c\omega_\alpha/(2\pi\omega_c)$, in agreement with the finding in Ref. [28] that when the bosonic cavity operators $\hat{a}_c$, $\hat{a}_c^\dagger$ couple directly to the reservoir field in the system-reservoir Hamiltonian, the resulting spectral density should scale with $\sim\omega$.

Using these results, one could transform to the Coulomb gauge using the $\hat{\mathcal{W}}$ operators, with the vector potential expressed in terms of the $\mathbf{L}^{(\mathbf{E})}$ projector functions. This is however, much less convenient than using the vector potential definition of the quantized QNMs from the main text, as in that case the transformation $\hat{\mathcal{W}}$ is expressed in terms of the transverse vector potential. Using $\mathbf{L}^{(\mathbf{E})}$, the transverse vector potential must be expressed in terms of both quantized QNM operators and residual reservoir operators, the latter of which have a non-local commutation relation which must be carefully accounted for, and additional parameters similar to the χ matrix would have to be defined.

V. JUSTIFICATION OF THE LOCAL BOSONIC COMMUTATOR ASSUMPTION

In this part, we give justification for the local bosonic commutator approximation of the reservoir operators $\hat{\mathbf{c}}(\mathbf{r},\omega)$, which have the exact commutator, given by Eq. (22). See also Ref. [31] for a more general proof. The time derivative of $\hat{c}_i(\mathbf{r},\omega)$ with respect to the bare reservoir Hamiltonian (interaction picture evolution) is given by

$$-\frac{i}{\hbar}[\hat{c}_i(\mathbf{r},\omega),H_{\rm R}] = -i\omega\hat{c}_i(\mathbf{r},\omega) + i\sum_\nu L^*_{\nu,i}(\mathbf{r},\omega)\sum_j\int_0^\infty d\omega'\int d^3r'\ \omega' L_{\nu,j}(\mathbf{r}',\omega')\hat{c}_j(\mathbf{r}',\omega'). \tag{94}$$

The first term is the usual bosonic contribution (free evolution), while the second term comes from the non-local commutator nature of $\hat{\mathbf{c}}(\mathbf{r},\omega)$. Using the projection rule $\int_0^\infty d\omega\int d^3r\mathbf{L}_\nu(\mathbf{r},\omega)\cdot\hat{\mathbf{c}}(\mathbf{r},\omega)=0$ (valid for all ν), the time evolution of the (exact) reservoir operators in the interaction picture can be calculated as

$$\hat{c}_i(\mathbf{r},\omega,t) = e^{-i\omega(t-t_0)}\hat{c}_i(\mathbf{r},\omega) + i\sum_\nu L^*_{\nu,i}(\mathbf{r},\omega)\sum_j\int d^3r'\int_0^\infty d\omega'\int_{t_0}^t dt' g_{\nu,j}(\mathbf{r}',\omega')\hat{c}_j(\mathbf{r}',\omega',t')e^{-i\omega(t-t')}, \tag{95}$$

and so

$$\begin{aligned}\hat{C}_\mu(t) =&\hbar\int d^3r\int_0^\infty d\omega\mathbf{g}_\mu(\mathbf{r},\omega)\cdot\hat{\mathbf{c}}(\mathbf{r},\omega)e^{-i\omega(t-t_0)}\\ &+ i\sum_{\nu\nu'\mu'}[S^{-1/2}]_{\mu\mu'}[S^{-1/2}]_{\nu'\nu}\int_{t_0}^t dt'\hat{C}_\nu(t')\int_0^\infty d\omega\frac{\tilde{g}_{\mu'\nu'}(\omega)}{\omega-\tilde{\omega}^*_{\nu'}}e^{-i\omega(t-t')},\end{aligned} \tag{96}$$

where $\hat{C}_\mu(t)=\hbar\int d^3r\int_0^\infty d\omega\mathbf{g}_\mu(\mathbf{r},\omega)\cdot\hat{\mathbf{c}}(\mathbf{r},\omega,t)$, and $g_{\mu\eta}(\omega)=\sum_{\mu'\nu'}\left[S^{-1/2}\right]_{\mu\mu'}\left[S^{-1/2}\right]_{\nu'\nu}\tilde{g}_{\mu'\nu'}(\omega)$.

Next, if we can make a Markov approximation $\tilde{g}_{\mu'\nu'}(\omega)\approx\tilde{g}_{\mu'\nu'}$ (assuming $\tilde{g}_{\mu'\nu'}(\omega)$ to vary slowly over the scale of κ_μ,κ_ν, equivalent to a relatively high Q approximation), and extend the frequency integral to $-\infty$, we can use the residue theorem to see

$$\int_{-\infty}^\infty d\omega\frac{e^{-i\omega(t-t')}}{\omega-\tilde{\omega}^*_{\nu'}}=0, \tag{97}$$

since $t-t'>0$, and $\tilde{\omega}^*_\nu$ is always located in the upper complex half plane. Thus, within the bounds of the Markov approximation,

$$\hat{C}_\mu(t)=\hbar\int d^3r\int_0^\infty d\omega\mathbf{g}_\mu(\mathbf{r},\omega)\cdot\hat{\mathbf{c}}(\mathbf{r},\omega)e^{-i\omega(t-t_0)}. \tag{98}$$

Subsequently, we show that the trace over the reservoir subsystem in the ME can also be evaluated as if the reservoir operators had local bosonic commutation relations. We assume that the state of the reservoir is the vacuum state [which is implicitly defined from the modes of the universe operators $\hat{\mathbf{b}}(\mathbf{r},\omega)$], and evaluate:

$$\begin{aligned}\frac{1}{\hbar^2}{\rm Tr}_R\left[\hat{C}_\mu(t)\hat{C}^\dagger_\nu(t-\tau)\rho_R\right] =& \int_0^\infty d\omega g_{\mu\eta}(\omega)e^{-i\omega\tau}\\ &-\sum_{\mu'\nu'\mu''\nu''}\left[[S^{-1/2}]_{\mu\mu'}\int_0^\infty d\omega\frac{\tilde{g}_{\mu'\nu'}(\omega)e^{-i\omega(t-t_0)}}{\omega-\tilde{\omega}^*_{\nu'}}\right]\\ &\times\left[[S^{-1/2}]_{\mu''\nu}[S^{-1}]_{\nu'\nu''}\int_0^\infty d\omega\frac{\tilde{g}_{\nu''\mu''}(\omega)e^{i\omega(t-\tau-t_0)}}{\omega-\tilde{\omega}_{\nu''}}\right].\end{aligned} \tag{99}$$

Making the Markov approximation again and extending the frequency integral bounds, the second term vanishes (within the same argumentation as further above), and we are left with the same result as if we assumed bosonic commutation relations.

The arguments developed in this section should also be applicable to the case where longitudinal interactions are included via $\hat{B}_\parallel$, although in this case some more assumptions on the specific form of $\mathbf{G}^\parallel(\mathbf{0},\mathbf{r},\omega)$ may be needed, in principle.

VI. PERTURBATIVE ANALYTIC EXPRESSION FOR LINEWIDTHS UNDER WEAK-EXCITATION

In this section, we derive a perturbative solution for the linewidths of the intracavity spectrum under weak excitation. Our starting point is the Coulomb gauge system Hamiltonian of Eq. (7) in the main text, expanded to order $|\eta_c|^2$

(here we let $\omega_0 = \omega_c$ for simplicity, though this is not necessary),

$$\hat{\mathcal{H}}_{\mathrm{C}}^{\mathrm{S}} = \hbar\omega_0 \hat{a}_c^\dagger \hat{a}_c + \frac{\hbar\omega_0}{2}\left[1 - |\eta_c|^2 \left(\hat{a}_c + \hat{a}_c^\dagger\right)^2\right]\hat{\sigma}_z + \hbar\omega_0|\eta_c|(\hat{a}_c + \hat{a}_c^\dagger)\hat{\sigma}_x + \mathcal{O}(|\eta_c|^3), \tag{100}$$

and here we have performed a unitary transformation on the cavity and TLS operators to eliminate the phase of η_c, which does not change any of our results due to the phase-insensitivity of the cavity-reservoir coupling. We can perturbatively diagonalize this Hamiltonian by means of the Bloch-Siegert (BS) transformation [41]

$$\hat{U}_{\mathrm{BS}} = \exp\left[\frac{|\eta_c|}{2}\left(\hat{a}_c\hat{\sigma}^- - \hat{a}_c^\dagger\hat{\sigma}^+\right)\right], \tag{101}$$

and we find

$$\begin{aligned}\hat{\mathcal{H}}_{\mathrm{BS}} &= \hat{U}_{\mathrm{BS}}^\dagger \hat{\mathcal{H}}_{\mathrm{C}}^{\mathrm{S}} \hat{U}_{\mathrm{BS}} \\ &= \hbar\omega_0\left[1 + \frac{3}{2}|\eta_c|^2\right]\hat{a}_c^\dagger\hat{a}_c + \hbar\omega_0\left[1 - \frac{3}{2}|\eta_c|^2\right]\hat{\sigma}^+\hat{\sigma}^- \\ &+ \hbar\omega_0|\eta_c|\left(\hat{a}_c\hat{\sigma}^+ + \hat{a}_c^\dagger\hat{\sigma}^-\right),\end{aligned} \tag{102}$$

and we have dropped both terms proportional to $\hat{a}_c^2$ and $\hat{a}_c^{\dagger 2}$, which can be removed by another transformation without any change in the Hamiltonian to order $|\eta_c|^2$, as well as terms diagonal in higher-order states beyond the lowest 2 excited energy levels [41, 56]. We have also added a constant energy term to the Hamiltonian to set the ground state $|G\rangle$ energy at zero. By diagonalizing Eq. (102), we obtain, for the two lowest-energy excited states,

$$E_\pm = \hbar\omega_0 \pm \hbar|\eta_c|\sqrt{1 + \frac{9}{4}|\eta_c|^2}, \tag{103a}$$

$$|\pm\rangle = \frac{1}{\sqrt{2}}\left(1 \pm \frac{3}{4}|\eta_c|\right)|1, g\rangle + \frac{1}{\sqrt{2}}\left(\pm 1 - \frac{3}{4}|\eta_c|\right)|0, g\rangle, \tag{103b}$$

and the state vectors have been expanded to order $|\eta_c|$. In the limit of well-separated spectral peaks (strong coupling), the full width at half maxima are [41]

$$\Gamma_\pm = \frac{\Lambda^2(E_\pm/\hbar)}{2\pi}\langle G|\hat{U}_{\mathrm{BS}}^\dagger\hat{a}_c\hat{U}_{\mathrm{BS}}|\pm\rangle, \tag{104}$$

which, upon expanding to leading order in $|\eta_c|$, gives the result from Eq. (13) in the main text.

[1] H. T. Dung, L. Knöll, and D.-G. Welsch, Three-dimensional quantization of the electromagnetic field in dispersive and absorbing inhomogeneous dielectrics, Physical Review A **57**, 3931 (1998).
[2] T. Gruner and D.-G. Welsch, Green-function approach to the radiation-field quantization for homogeneous and inhomogeneous Kramers-Kronig dielectrics, Physical Review A **53**, 1818 (1996).
[3] L. G. Suttorp and A. J. van Wonderen, Fano diagonalization of a polariton model for an inhomogeneous absorptive dielectric, Europhysics Letters **67**, 766 (2004).
[4] C. Gustin, S. Franke, and S. Hughes, Gauge-invariant theory of truncated quantum light-matter interactions in arbitrary media, Physical Review A **107**, 013722 (2023).
[5] W. Vogel and D.-G. Welsch, *Quantum Optics. 3rd edn.* (Weinheim: Wiley-VCH Verlag GmbH Co. KGaA, 2006).
[6] T. G. Philbin, Canonical quantization of macroscopic electromagnetism, New Journal of Physics **12**, 123008 (2010).
[7] S. Franke, S. Hughes, M. K. Dezfouli, P. T. Kristensen, K. Busch, A. Knorr, and M. Richter, Quantization of quasinormal modes for open cavities and plasmonic cavity quantum electrodynamics, Physical Review Letters **122**, 213901 (2019).
[8] S. Franke, J. Ren, S. Hughes, and M. Richter, Fluctuation-dissipation theorem and fundamental photon commutation relations in lossy nanostructures using quasinormal modes, Physical Review Research **2**, 033332 (2020).
[9] E. S. C. Ching, P. T. Leung, A. Maassen van den Brink, W. M. Suen, S. S. Tong, and K. Young, Quasinormal-mode expansion for waves in open systems, Reviews of Modern Physics **70**, 1545 (1998).
[10] P. T. Kristensen and S. Hughes, Modes and mode volumes of leaky optical cavities and plasmonic nanoresonators, ACS Photonics **1**, 2 (2014).
[11] P. Lalanne, W. Yan, K. Vynck, C. Sauvan, and J.-P. Hugonin, Light interaction with photonic and plasmonic resonances, Laser & Photonics Reviews **12**, 1700113 (2018).
[12] P. T. Kristensen, K. Herrmann, F. Intravaia, and K. Busch, Modeling electromagnetic resonators using quasinormal modes, Advances in Optics and Photonics **12**, 612 (2020).
[13] M. K. Dezfouli, R. Gordon, and S. Hughes, Molecular optomechanics in the anharmonic cavity-QED regime using hybrid metal-dielectric cavity modes, ACS Photonics **6**, 1400 (2019).
[14] M. K. Dezfouli, C. Tserkezis, N. A. Mortensen, and S. Hughes, Nonlocal quasinormal modes for arbitrarily shaped three-dimensional plasmonic resonators, Optica **4**, 1503 (2017).
[15] S. Franke, M. Richter, J. Ren, A. Knorr, and S. Hughes, Quantized quasinormal-mode description of nonlinear cavity-QED effects from coupled resonators with a Fano-like resonance, Physical Review Research **2**, 033456 (2020).
[16] L. Zschiedrich, F. Binkowski, N. Nikolay, O. Benson, G. Kewes, and S. Burger, Riesz-projection-based theory of light-matter interaction in dispersive nanoresonators, Physical Review A **98**, 043806 (2018).
[17] W. Li, Q. Zhou, P. Zhang, and X.-W. Chen, Bright optical eigenmode of 1 nm^3 mode volume, Physical Review Letters **126**, 257401 (2021).
[18] K. M. Lee, P. T. Leung, and K. M. Pang, Dyadic formulation of morphology-dependent resonances. I. completeness relation, Journal of the Optical Society of America B **16**, 1409 (1999).
[19] P. T. Leung, S. Y. Liu, and K. Young, Completeness and orthogonality of quasinormal modes in leaky optical cavities, Physical Review A **49**, 3057 (1994).
[20] R.-C. Ge, P. T. Kristensen, J. F. Young, and S. Hughes, Quasinormal mode approach to modelling light-emission and propagation in nanoplasmonics, New Journal of Physics **16**, 113048 (2014).
[21] R.-C. Ge and S. Hughes, Design of an efficient single photon source from a metallic nanorod dimer: a quasi-normal mode finite-difference time-domain approach, Optics Letters **39**, 4235 (2014).
[22] M. Kamandar Dezfouli, R. Gordon, and S. Hughes, Modal theory of modified spontaneous emission for a hybrid plasmonic photonic-crystal cavity system, Physical Review A **95**, 013846 (2017).
[23] C. Carlson and S. Hughes, Dissipative modes, purcell factors, and directional beta factors in gold bowtie nanoantenna structures, Physical Review B **102**, 155301 (2020).
[24] J. Ren, S. Franke, A. Knorr, M. Richter, and S. Hughes, Near-field to far-field transformations of optical quasinormal modes and efficient calculation of quantized quasinormal modes for open cavities and plasmonic resonators, Physical Review B **101**, 205402 (2020).
[25] S. Franke, J. Ren, and S. Hughes, Impact of mode regularization for quasinormal-mode perturbation theories, Physical Review A **108**, 043502 (2023).
[26] J. Ren, S. Franke, A. Knorr, M. Richter, and S. Hughes, Near-field to far-field transformations of optical quasinormal modes and efficient calculation of quantized quasinormal modes for open cavities and plasmonic resonators, Physical Review B **101**, 205402 (2020).
[27] S. Schelkunoff, Some equivalence theorems of electromagnetics and their application to radiation problems, The Bell System Technical Journal **15**, 92 (1936).
[28] C. Gustin, J. Ren, and S. Hughes, What is the spectral density of the reservoir for a lossy quantized cavity?, Physical Review Letters **134**, 123601 (2025).
[29] S. Scheel, L. Knöll, and D.-G. Welsch, Spontaneous decay of an excited atom in an absorbing dielectric, Physical Review A **60**, 4094 (1999).
[30] V. S. C. Manga Rao and S. Hughes, Single quantum-dot Purcell factor and β factor in a photonic crystal waveguide, Physical Review B **75**, 205437 (2007).

[31] C. Gustin, Open system techniques for broadband quantum optics: Beyond phenomenology, PhD thesis, Stanford University, Stanford (2025).
[32] S. Franke, J. Ren, M. Richter, A. Knorr, and S. Hughes, Fermi's golden rule for spontaneous emission in absorptive and amplifying media, Physical Review Letters **127**, 013602 (2021).
[33] S. Franke, J. Ren, and S. Hughes, Quantized quasinormal-mode theory of coupled lossy and amplifying resonators, Physical Review A **105**, 023702 (2022).
[34] S. Savasta, O. Di Stefano, A. Settineri, D. Zueco, S. Hughes, and F. Nori, Gauge principle and gauge invariance in two-level systems, Physical Review A **103**, 053703 (2021).
[35] A. Settineri, O. Di Stefano, D. Zueco, S. Hughes, S. Savasta, and F. Nori, Gauge freedom, quantum measurements, and time-dependent interactions in cavity QED, Physical Review Research **3**, 023079 (2021).
[36] W. E. Lamb, R. R. Schlicher, and M. O. Scully, Matter-field interaction in atomic physics and quantum optics, Physical Review A **36**, 2763 (1987).
[37] P. W. Milonni, R. J. Cook, and J. R. Ackerhalt, Natural line shape, Physical Review A **40**, 3764 (1989).
[38] D. De Bernardis, P. Pilar, T. Jaako, S. De Liberato, and P. Rabl, Breakdown of gauge invariance in ultrastrong-coupling cavity QED, Physical Review A **98**, 053819 (2018).
[39] O. Di Stefano, A. Settineri, V. Macrì, L. Garziano, R. Stassi, S. Savasta, and F. Nori, Resolution of gauge ambiguities in ultrastrong-coupling cavity quantum electrodynamics, Nature Physics **15**, 803 (2019).
[40] M. A. D. Taylor, A. Mandal, W. Zhou, and P. Huo, Resolution of gauge ambiguities in molecular cavity quantum electrodynamics, Physical Review Letters **125**, 123602 (2020).
[41] W. Salmon, C. Gustin, A. Settineri, O. Di Stefano, D. Zueco, S. Savasta, F. Nori, and S. Hughes, Gauge-independent emission spectra and quantum correlations in the ultrastrong coupling regime of open system cavity-QED, Nanophotonics **11**, 1573 (2022).
[42] M. A. D. Taylor, A. Mandal, P. Huo, and P. Huo, Resolving ambiguities of the mode truncation in cavity quantum electrodynamics, Optics Letters **47**, 1446 (2022).
[43] M. Wubs, L. G. Suttorp, and A. Lagendijk, Multipole interaction between atoms and their photonic environment, Physical Review A **68**, 013822 (2003).
[44] A. Settineri, V. Macrí, A. Ridolfo, O. Di Stefano, A. F. Kockum, F. Nori, and S. Savasta, Dissipation and thermal noise in hybrid quantum systems in the ultrastrong-coupling regime, Physical Review A **98**, 053834 (2018).
[45] M. Bamba and T. Ogawa, Recipe for the Hamiltonian of system-environment coupling applicable to the ultrastrong-light-matter-interaction regime, Physical Review A **89**, 023817 (2014).
[46] H.-P. Breuer and F. Petruccione, *The Theory of Open Quantum Systems.* (Oxford: Oxford University Press, 2002).
[47] D. M. Toyli, A. W. Eddins, S. Boutin, S. Puri, D. Hover, V. Bolkhovsky, W. D. Oliver, A. Blais, and I. Siddiqi, Resonance fluorescence from an artificial atom in squeezed vacuum, Physical Review X **6**, 031004 (2016).
[48] H. J. Carmichael, A. S. Lane, and D. F. Walls, Resonance fluorescence from an atom in a squeezed vacuum, Physical Review Letters **58**, 2539 (1987).
[49] A. Mercurio, V. Macrì, C. Gustin, S. Hughes, S. Savasta, and F. Nori, Regimes of cavity QED under incoherent excitation: from weak to deep strong coupling, Physical Review Research **4**, 023048 (2022).
[50] C. Gustin, Gauge invariance of the natural lineshape and dissipative dynamics of a two-level system, Physical Review A **112**, 053709 (2025).
[51] T. V. Shahbazyan, Interacting quantum plasmons in metal-dielectric structures, Physical Review B **103**, 045421 (2021).
[52] T. V. Shahbazyan, Non-Markovian effects for hybrid plasmonic systems in the strong coupling regime, Physical Review B **105**, 245411 (2022).
[53] J. Ren, S. Franke, and S. Hughes, Connecting classical and quantum mode theories for coupled lossy cavity resonators using quasinormal modes, ACS Photonics **9**, 138 (2022).
[54] M. Kamandar Dezfouli, R. Gordon, and S. Hughes, Modal theory of modified spontaneous emission of a quantum emitter in a hybrid plasmonic photonic-crystal cavity system, Physical Review A **95**, 013846 (2017).
[55] S. R. Franke, Quantization of quasinormal modes in dissipative media, PhD Thesis, Technical University of Berlin, Berlin (2020).
[56] S. Hughes, C. Gustin, and F. Nori, Reconciling quantum and classical spectral theories of ultrastrong coupling: role of cavity bath coupling and gauge corrections, Optica Quantum **2**, 133 (2024).
